# SAGES guidelines for the use of laparoscopy during pregnancy

**DOI:** 10.1007/s00464-024-10810-1

**Published:** 2024-05-03

**Authors:** Sunjay S. Kumar, Amelia T. Collings, Claire Wunker, Dimitrios I. Athanasiadis, Colin G. DeLong, Julie S. Hong, Mohammed T. Ansari, Ahmed Abou-Setta, Emily Oliver, Vincenzo Berghella, Vamsi Alli, Imran Hassan, Celeste Hollands, Patricia Sylla, Bethany J. Slater, Francesco Palazzo

**Affiliations:** 1 Department of Surgery, Thomas Jefferson University Hospital, Philadelphia, PA USA; 2 Hiram C. Polk, Jr Department of Surgery, University of Louisville, Louisville, KY USA; 3 Department of Surgery, Saint Louis University, Saint Louis, MO USA; 4 Department of Surgery, Indiana University School of Medicine, Indianapolis, IN USA; 5 Department of Surgery, Pennsylvania State University College of Medicine, Hershey, PA USA; 6 Department of Surgery, NewYork-Presbyterian Queens, New York, NY USA; 7 School of Epidemiology and Public Health, Faculty of Medicine, University of Ottawa, Ottawa, ON Canada; 8 Centre for Healthcare Innovation, University of Manitoba, Winnipeg, MB Canada; 9 Department of Obstetrics, Gynecology and Reproductive Sciences, Lewis Katz School of Medicine at Temple University, Philadelphia, PA USA; 10 Department of Obstetrics & Gynecology, Thomas Jefferson University Hospital, Philadelphia, PA USA; 11 Department of Surgery, Mercy Medical Center Cedar Rapids, Cedar Rapids, IA USA; 12 Department of Surgery, Texas Tech University Health Sciences Center, Lubbock, TX USA; 13 Division of Colon and Rectal Surgery, Mount Sinai Health System, New York, NY USA; 14 Department of Surgery, University of Chicago Medicine, Chicago, IL USA; 15 Thomas Jefferson University, 1100 Walnut Street, Suite 500, Philadelphia, PA 19107 USA

**Keywords:** Guidelines, Surgery in pregnancy, Appendicitis, Cholecystitis, Biliary disease, Inflammatory bowel disease, ERCP

## Abstract

**Background:**

When pregnant patients present with nonobstetric pathology, the physicians caring for them may be uncertain about the optimal management strategy. The aim of this guideline is to develop evidence-based recommendations for pregnant patients presenting with common surgical pathologies including appendicitis, biliary disease, and inflammatory bowel disease (IBD).

**Methods:**

The Society of American Gastrointestinal and Endoscopic Surgeons (SAGES) Guidelines Committee convened a working group to address these issues. The group generated five key questions and completed a systematic review and meta-analysis of the literature. An expert panel then met to form evidence-based recommendations according to the Grading of Recommendations Assessment, Development, and Evaluation approach. Expert opinion was utilized when the available evidence was deemed insufficient.

**Results:**

The expert panel agreed on ten recommendations addressing the management of appendicitis, biliary disease, and IBD during pregnancy.

**Conclusions:**

Conditional recommendations were made in favor of appendectomy over nonoperative treatment of appendicitis, laparoscopic appendectomy over open appendectomy, and laparoscopic cholecystectomy over nonoperative treatment of biliary disease and acute cholecystitis specifically. Based on expert opinion, the panel also suggested either operative or nonoperative treatment of biliary diseases other than acute cholecystitis in the third trimester, endoscopic retrograde cholangiopancreatography rather than common bile duct exploration for symptomatic choledocholithiasis, applying the same criteria for emergent surgical intervention in pregnant and non-pregnant IBD patients, utilizing an open rather than minimally invasive approach for pregnant patients requiring emergent surgical treatment of IBD, and managing pregnant patients with active IBD flares in a multidisciplinary fashion at centers with IBD expertise.

**Graphical abstract:**

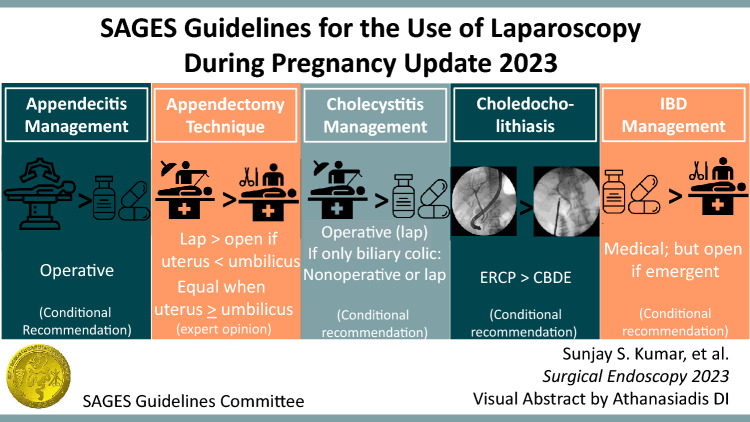

**Supplementary Information:**

The online version contains supplementary material available at 10.1007/s00464-024-10810-1.

## Aim of these guidelines and specific objectives

The purpose of these guidelines is to provide evidence-based recommendations for the management of common surgical problems during pregnancy from a surgeon and patient perspective. We assessed surgical and obstetric outcomes after management of appendicitis, biliary disease, and inflammatory bowel disease (IBD) during pregnancy. The review evaluated operative versus nonoperative management and laparoscopic (LA) versus open appendectomy (OA) in acute appendicitis; operative versus nonoperative management for biliary disease; common bile duct exploration versus endoscopic retrograde cholangiopancreatography (ERCP) for choledocholithiasis; and operative versus nonoperative management of IBD. The target audience for these guidelines includes patients, obstetricians, maternal–fetal medicine physicians, surgeons, and physicians who care for pregnant patients in clinical practice. A patient–physician perspective was taken so cost-effectiveness, resource requirements, and availability of said resources were not evaluated.

## Description of the health problems

Intra-abdominal pathologies during pregnancy present a challenging problem for physicians and patients when weighing the risks and benefits of surgical treatment. When operative management is mandated, the decision is easier but when nonoperative management is a viable option, the decision becomes more complicated. Patients and physicians with less experience treating pregnant patients may wonder how the treatment may affect the pregnancy. Appendicitis and cholecystitis are the most common nonobstetric emergencies encountered, with appendicitis occurring in 0.05–0.15% of pregnancies [1, 2]. While appendectomy is the historical gold-standard treatment, recent randomized controlled trials have demonstrated the efficacy of antibiotic therapy alone [3–8]. However, pregnant women were excluded from these trials.

Hormonal changes during pregnancy may predispose patients to gallstone formation [9]. The literature estimates a 0.01–0.06% incidence of cholecystitis during pregnancy, though modern data are lacking [10]. While biliary colic may at times be managed expectantly with postpartum cholecystectomy, acute diseases such as cholecystitis and choledocholithiasis must be managed more expeditiously.

While less common than acute appendicitis, inflammatory bowel disease (IBD) is often diagnosed during reproductive age making this population vulnerable during pregnancy. Surgical emergencies may occur due to bowel perforation or persistent bowel obstruction. However, chronic symptoms of IBD can also significantly impact quality of life and nutrition. Nonoperative management in these patients is the mainstay treatment but surgical resection is sometimes necessary. IBD has been associated with prematurity, low birth weight, congenital anomalies, and a higher rate of cesarean section [11].

## How to use these guidelines

The aim of these guidelines is to assist surgeons, obstetricians, and other physicians involved in the care of pregnant patients to make decisions about the management of certain disease processes. These guidelines are also intended to provide education, inform advocacy, and describe future areas for research. While these are meant to highlight the optimal approach in a generalized patient population, distinct patient needs, comorbidities, and specific situations may require adjustments to determine the ideal treatment for each individual. In addition, these guidelines can serve as a resource for patients to promote discussion with their physicians.

## Methods

A systematic review of the literature informed the guideline recommendations. The panel developed and graded the recommendations employing the Grading of Recommendations Assessment, Development and Evaluation (GRADE) approach, the GRADE guideline development tool, and the Essential Reporting Items for Practice Guidelines in Healthcare (RIGHT) checklist [12–14].

### Guideline panel organization

An expert panel was selected from within the SAGES Guidelines committee to create a systematic review for this laparoscopy in pregnancy guideline. The systematic review was overseen by a methodologist with systematic review expertise (A.A.). The panel was composed of practicing surgeons from the Society of American Gastrointestinal and Endoscopic Surgeons and maternal–fetal medicine physicians (E.O. and V.B.). A methodologist (M.T.A.) with guideline development expertise and the guideline committee fellow (S.S.K.) facilitated guideline panel meetings as non-voting members of the panel. The panel used the GRADE methodology to assess the evidence from the systematic review and judge certainty of evidence and the strength of guideline recommendation [13]. Full author roles are listed in Online Appendix A.

### Guideline funding & declaration and management of competing interests

SAGES provided funding for the librarian who assisted with the systematic review, the methodologists, and the Guidelines Committee Fellow. Industry did not provide any financial support nor any input into the conception or development of this guideline. A standard SAGES conflict of interest form was collected from all guideline contributors by the guideline lead. A full list of declarations is listed in Online Appendix G.

### Selection of questions and outcomes of interest

The guideline panelists formulated key questions relevant to the use of laparoscopy in pregnancy according to the Population, Intervention, Comparison, Outcome (PICO) format, in consultation with the methodologist, guideline lead (F.P.), and committee Chair (B.S.). Key questions were approved by a SAGES Guidelines committee working group.

The panel members used their extensive clinical experience to identify patient-centered outcomes they believed most surgeon–patient dyads would consider important to decision-making. These outcomes were chosen based on panel consensus by simple majority and then further designated as critical or important to decision-making on the basis of their relative importance to patients. This designation was confirmed by panel members during the formulation of recommendations after reviewing the evidence from the systematic review.

### Evidence review and synthesis

A systematic review addressing the KQs was conducted according to the SAGES Guidelines Committee’s standard operating procedure [15]. The Cochrane Library, Clinicaltrials.gov, Embase, PubMed, and the International Clinical Trials Registry Platform databases were searched from 1990 to 2021 for evidence, ultimately only yielding observational studies. When no direct comparative studies were available, non-comparative evidence was utilized. Search strategies can be found in Online Appendix B.

Each record was screened by two independent reviewers at both the abstract and full text review phases. Screening criteria and the Preferred Reporting Items for Systematic Reviews and Meta-Analyses (PRISMA) can be found in Online Appendix C. As there were no RCT data found for any question, study quality was assessed using the Newcastle Ottawa scale. Random effects meta-analysis was performed on the extracted data. Forest plots can be found in Online Appendix D.

### Determining the certainty of evidence

As per the Guidelines Committee’s standard operating procedure, the GRADE approach was utilized to judge the certainty of evidence available for each outcome. The highest level of evidence identified was imported into GRADEPro evidence tables. The certainty of this evidence was evaluated on the basis of its risk of bias, inconsistency, indirectness, and imprecision. The certainty was downgraded based on the number of domains across which there were concerns. These data were then imported into an Evidence to Decision table for each KQ which provided the framework through which the expert panel developed its recommendations. Evidence tables and Evidence to Decision tables can be found in Online Appendices E and F, respectively.

### Assumed values and preferences

The panel members used clinical experience to inform judgment on the valuation of different outcomes on behalf of patients. This expertise was deemed likely sufficient to anticipate the variation in values by patients informed by the same evidence. Empiric evidence of how patients value these outcomes was not searched, so the panel’s was used as a proxy for patient values and preferences.

### Development of recommendations

The panel convened virtually in the autumn of 2022 to review the evidence and make recommendations. The results of the systematic review and the articles utilized were available for independent review prior to the meetings. During the meetings, the panel members reviewed the evidence and completed the Evidence to Decision tables to generate recommendations. This process entailed deliberating the magnitude of desirable and undesirable effects, the certainty of evidence, and variation in how patients may value outcomes. After this, the panel voted on whether the overall balance of these considerations favored the intervention or comparison. The panel then discussed the acceptability and feasibility of this judgment. For each decision, the panel took into account the results of the systematic review and pertinent considerations from panel member’s clinical experience or interpretation of the evidence. Based on the balance of effects and the acceptability and feasibility of a favored option, the panel voted on the final recommendation for that key question. While serial voting was used to come to a consensus on individual components of the EtD, 80% agreement was mandatory for all final recommendations.

Subgroups, such as trimester, were discussed in the justification for each recommendation and are specified for each KQ where relevant. Full evidence to decision tables are presented in Online Appendix F and summarized in the following recommendations.

### Guideline document review

This guideline was drafted based on the evidence to decision tables and panel discussion and was edited by all panel members. In accordance with SAGES Guidelines Committee policies, the final draft was distributed to the committee for approval or suggested changes. After incorporating these edits, the Guideline was then submitted to SAGES Executive board for approval and published online for public comment for 4 weeks.

### Recommendation for future research

The authors have provided a concise and comprehensive review of potential avenues for future research for each KQ discussed. These were made based on existing gaps in the literature identified during the review process. To minimize repetition, these recommendations are listed in the discussion.

#### Key questions

**KQ1** Should appendectomy (laparoscopic or open) versus nonoperative treatment (antibiotics) be used for acute appendicitis during pregnancy (any trimester)?

**KQ2** Should laparoscopic appendectomy versus open appendectomy be used for acute appendicitis during pregnancy (any trimester)?

**KQ3** Should laparoscopic cholecystectomy versus nonoperative treatment be used for the management of biliary disease during pregnancy (any trimester)?

**KQ4** Should common bile duct exploration (CBDE) versus ERCP be used for symptomatic choledocholithiasis during pregnancy (any trimester)?

**KQ5** Should laparoscopic intestinal resection versus observation/deferred intervention be used for inflammatory bowel disease during pregnancy (any trimester)?

## Recommendations

### KQ1 should appendectomy (laparoscopic or open) versus nonoperative treatment (antibiotics) be used for acute appendicitis during pregnancy (any trimester)?

The panel suggests that appendectomy rather than nonoperative treatment should be used for acute appendicitis during pregnancy (*conditional recommendation, very low certainty of evidence*).

#### Introduction

The incidence of acute appendicitis during pregnancy is between 0.05 and 0.15%, making it the most common nonobstetric surgical disease process in pregnancy [1, 2]. While the health of the mother must always be prioritized, the potential for adverse fetal outcomes is often an important consideration to these patients. A large population-based study found that appendicitis during pregnancy increases the risks of preterm birth (OR 2.68), intra-amniotic infection (OR 2.25), and intrauterine death (OR 1.45) [16]. These risks are even higher in cases of perforated appendicitis [17].

Based on recent national and international [3–8] data, antibiotics are an effective option for the treatment of uncomplicated appendicitis in adults. However, this has not been demonstrated in the pregnant population.

The previously published SAGES guideline on the use of laparoscopy in pregnancy stated that “Laparoscopic appendectomy is the treatment of choice for pregnant patients with acute appendicitis (++; Weak)” [18]. The panel added that “there is no role for nonoperative management of uncomplicated acute appendicitis in pregnant women because of a higher rate of peritonitis, fetal demise, shock, and venous thromboembolism as compared to operative management.”

#### Summary of the evidence

The panel’s recommendation was informed by data from four observational studies [16, 19–21]. The panel noted that one study included a statistically significantly higher number of patients with complicated appendicitis in the operative arm [21]. All studies included in the analysis were retrospective in nature and likely subject to significant selection bias. The main limitation was that a large portion of studies ultimately included in panel decision-making were deemed high risk of bias due to concerns over the comparability of the two groups, leading to the overall very low certainty of evidence.

Preterm birth, pregnancy loss, and sepsis were designated critical while cesarean section, delivery during admission, and readmission were deemed important to clinical decision-making. The certainty of evidence for all outcomes was very low, primarily based on small sample sizes, low event rates, and differences between the surgical and medical groups that limited their comparability.

#### Benefits

There were three outcomes with desirable effects for appendectomy: rate of delivery via cesarean section (at any time), readmission, and sepsis. The combined magnitude of these favorable effects was determined to be small by the panel.

*Cesarean section* estimated 66 fewer per 1000 patients (95% CI 204 fewer to 223 more) based on 1 study with 54 patients [20].

*Readmission* estimated 67 fewer per 1000 patients (95% CI 87 fewer to 214 more) based on 1 study with 54 patients [20].

*Sepsis* estimated 8 fewer per 1000 patients (95% CI 9 fewer to 5 fewer) based on 1 study with 7114 patients [16].

#### Harms and burdens

Of the outcomes deemed critical or important for decision-making, there were two with undesirable effects for appendectomy: pregnancy loss (total, at any gestational age) and preterm birth. Overall, the panel felt the combined magnitude for these undesirable effects for appendectomy was trivial based on the small absolute event rates.

*Pregnancy loss* estimated 11 more per 1000 patients (95% CI 23 fewer to 119 more) based on 3 studies with 243 patients [19–21].

*Preterm birth* estimated 8 more per 1000 patients (95% CI 48 fewer to 261 more) based on 2 observational studies with 74 patients [19, 20].

#### Decision criteria and additional considerations

The panel agreed laparoscopic appendectomy during pregnancy would probably be acceptable to key stakeholders but noted potential concerns regarding feasibility. The uncertainty surrounding feasibility hinged primarily on the availability of obstetrics support, especially for patients at more advanced gestational ages. Patients presenting to facilities with limited resources or minimal obstetric, anesthetic, or neonatal expertise would require transfer to another facility.

The panel acknowledged the existing literature on nonoperative treatment in the adult non-pregnant population but agreed that those results cannot be extrapolated to the pregnant population [3–8].

The panel discussed how gestational age and fetal viability may influence the discussion with the patient. There was no evidence that a patient’s gestational age would alter this recommendation nor could the panel identify plausible reasons that it would; therefore, this conditional recommendation applies across all trimesters but the panel acknowledges the decision to pursue surgical treatment must be a shared decision based on patient-specific factors.

The expert panel also noted that based on ACOG recommendations, the obstetrics team should be consulted for all pregnant patients undergoing nonobstetric operations to determine any peri- or intraoperative monitoring needs during the surgical course.

#### Conclusions

The panel agreed that the balance of effects favored appendectomy over nonoperative treatment and therefore suggests appendectomy for pregnant patients with acute appendicitis regardless of trimester (*conditional recommendation, very low certainty of evidence*).

#### What others are saying

The European Association for Endoscopic Surgery (EAES) recently published their own guidelines aimed at this question [22]. They recommended operative treatment when pregnant patients have complicated appendicitis or an appendicolith (strong recommendation) and suggested operative treatment in uncomplicated cases (conditional recommendation). The concern over adverse fetal outcomes in cases of failed nonoperative management seems to have been the driver behind these recommendations.

The American College of Obstetricians and Gynecologists (ACOG) published a clinical practice guideline on Nonobstetric Surgery during Pregnancy in 2017 which was reaffirmed in 2021 [23]. Their most relevant recommendation is “A pregnant woman should never be denied medically necessary surgery or have that surgery delayed regardless of trimester because this can adversely affect the pregnant woman and her fetus.”

### KQ2 should laparoscopic appendectomy versus open appendectomy be used for acute appendicitis during pregnancy (any trimester)?

The panel suggests that laparoscopic appendectomy rather than open appendectomy should be used for acute appendicitis when the fundus of the uterus is below the umbilicus (*expert opinion due to low quality of evidence*).

The panel suggests that laparoscopic or open appendectomy should be used for acute appendicitis when the fundus of the uterus is above the umbilicus, at the surgeon’s discretion. The panel additionally suggests the open establishment of pneumoperitoneum when the laparoscopic approach is utilized (*expert opinion due to low quality of evidence*).

#### Introduction

The use of laparoscopic or open appendectomy during pregnancy has been the subject of numerous systematic reviews with variable results [24]. The use of laparoscopy for other disease processes during pregnancy appears to be safe, without evidence of an increase in adverse maternal or fetal outcomes [25]. However, there are additional considerations in late gestation, such as the safety of laparoscopy when an enlarged uterus may prevent usual port site placement, limit working space, and be in harm’s way during the case.

#### Summary of the evidence

Data from 28 observational studies were used to inform the panel’s decision [17, 21, 26–51]. The panel noted that one study heavily impacted the outcome of pregnancy loss in favor of open appendectomy [17]. This study was carefully reviewed for its methodology and found to be at significant risk of bias. All studies included in the analysis were retrospective observational studies. The main limitation was that a large portion of studies ultimately included in panel decision-making were deemed high risk of bias due to the lack of comparability of the two groups. The studies also generally had small sample sizes and wide confidence intervals, leading to an overall very low certainty of evidence.

#### Benefits

There were four outcomes with desirable effects for LA: rate of delivery during the same admission, preterm delivery, readmission, and sepsis. The combined magnitude of these favorable effects was determined to be small by the panel.

*Delivery during admission* estimated 2 fewer per 1000 patients (95% CI 33 fewer to 453 more) based on 2 studies with 52 patients [26, 28].

*Preterm birth* 12 fewer per 1000 patients (95% CI 38 fewer to 28 more) based on 21 studies with 5983 patients [17, 27, 28, 30, 32, 33, 35–39, 41–45, 47–51].

*Readmission* 8 fewer per 1000 patients (95% CI 23 fewer to 19 more) based on 3 studies with 1094 patients [28, 36, 49].

*Sepsis* 3 fewer per 1000 patients (95% CI 6 fewer to 5 more) based on 2 studies with 2341 patients [34, 49].

#### Harms and burden

There were five outcomes with undesirable effects for LA: cesarean section, NICU admission, pregnancy loss (total, < 20 weeks, and > 20 weeks). The panel noted that obstetrics data collection is not optimal in the majority of the studies included in the analysis and discussed the national rates of pregnancy loss during the first trimester (20%) and third trimester (0.6%) [52, 53]. Additionally, the panel noted that one study weighted heavily on the pregnancy loss outcome; this study was considered at high risk of bias [17]. Overall, the panel felt the combined magnitude for these undesirable effects for surgery was trivial.

*Cesarean section *23 more per 1000 patients (95% CI 22 fewer to 69 more) based on 11 studies with 2266 patients [28, 30–33, 38, 40, 45, 47, 49, 51].

*Pregnancy loss* 27 more per 1000 patients (95% CI 11 more to 48 more) based on 27 studies with 6188 patients [17, 21, 26–33, 35–51].

#### Decision criteria and additional considerations

The panel agreed that there was probably no important uncertainty or variability in how patients value the main outcomes. Similarly, the panel agreed that the intervention is feasible to implement.

Similar to KQ1, the panel discussed several subgroups. Regarding the impact of advanced gestational age on the surgical approach, the expert panel suggested that past approximately 20 weeks of gestation or approximately when the gravid uterus reaches the umbilicus, the decision for LA or OA should be based on the expertise of the surgeon. However, if the laparoscopic approach is chosen, the panel felt closed establishment of pneumoperitoneum likely poses unnecessary risk to the patient and fetus. The severity of disease, complexity of the infectious process, patient’s hemodynamic stability, and prior surgical history may also affect the consideration for an open or laparoscopic approach.

#### Conclusions

The desirable anticipated effects for LA compared to OA were judged to be small and the undesirable effects were judged to be trivial. While the panel agreed that the balance of effects appeared to not significantly favor either, it voted to conditionally recommend in favor of LA when the fundus of the uterus is below the umbilicus given the evidence supporting superior outcomes in the non-pregnant population (*conditional recommendation, expert opinion*) [54, 55]. When the uterus is above the umbilicus, LA becomes more technically difficult and therefore the panel left the decision for LA or OA to the surgeon’s discretion (*conditional recommendation, expert opinion*).

#### What others are saying

The recently published EAES guidelines addressed the question of LA vs OA during pregnancy as well [22]. The authors made conditional recommendations for LA until 20 weeks of gestation or while the uterine fundus is below the umbilicus and laparoscopic appendectomy with open entry or open appendectomy otherwise. Their literature review also found a higher rate of pregnancy loss with LA but they acknowledge this is likely due to confounding factors with LA more likely to be utilized in early pregnancy when there is a higher baseline rate of pregnancy loss. They acknowledge the potential difficulty of LA in late gestation when the uterus occupies a greater portion of the peritoneal cavity and therefore leave this decision to the surgeon but do advocate against establishing pneumoperitoneum by Veress needle.

### KQ3 should laparoscopic cholecystectomy versus nonoperative treatment be used for the management of biliary disease during pregnancy (any trimester)?

The panel suggests that laparoscopic cholecystectomy should be used rather than nonoperative treatment for the management of biliary disease during pregnancy (*conditional recommendation, very low certainty evidence*).

For acute cholecystitis during pregnancy, the panel suggests laparoscopic cholecystectomy rather than nonoperative treatment (*conditional recommendation, low certainty of evidence*).

For biliary colic during the third trimester of pregnancy, the panel suggests either nonoperative treatment or laparoscopic cholecystectomy may be considered (*conditional recommendation, expert opinion due to low quality of evidence*).

#### Introduction

Cholecystectomy is the second most commonly performed nonobstetric abdominal operation during pregnancy [56]. The hormonal changes associated with pregnancy lead to higher concentrations of biliary cholesterol and biliary stasis, ultimately leading to formation of biliary sludge and stones [57, 58]. The treatment options vary depending on what clinical scenario along the spectrum of biliary disease the patient presents with. Biliary colic is often managed nonoperatively but has a high recurrence rate, especially when initially diagnosed early in pregnancy [59, 60].

For both biliary colic and acute cholecystitis, there has been debate around operative or nonoperative management during pregnancy. Nonoperative management is associated with a high recurrence rate and risks progression to a more severe form of biliary disease and complications, such as gallstone pancreatitis, which is associated with worse outcomes [61].

#### Summary of the evidence

A total of 16 retrospective, observational studies comparing laparoscopic cholecystectomy versus nonoperative treatment of biliary disease during pregnancy were identified during the literature search [60, 62–76]. These studies generally fell into one of two categories: single institution reviews with relatively small patient populations or reviews of large, national, administrative databases. Regardless of type, all included studies were found to be at high risk of bias due to concerns over the comparability of the two groups. Therefore, the overall certainty of the evidence was very low. The outcomes deemed critical to decision-making were sepsis, preterm birth, cesarean section, neonatal death, and pregnancy loss. The outcomes deemed important were delivery during admission, IUGR, NICU admission, preeclampsia, readmission, and bile leak.

#### Benefits

The benefits of laparoscopic cholecystectomy for biliary disease during pregnancy were found to include lower rates of cesarean section, delivery during admission, neonatal death, NICU admission, pregnancy loss, and readmission. Many of these differences were very small with confidence intervals which crossed the threshold of significance. However, the overall magnitude of the effect size was deemed moderate, with the outcomes of cesarean section and NICU admission noted as being particularly important in this vote.

*Cesarean section* estimated 32 fewer events per 1000 patients (95% CI 198 fewer to 183 more) based on 9 observational studies with 31,616 patients [65, 67–70, 72–75].

*Delivery during admission* estimated 77 fewer events per 1000 patients (95% CI 165 fewer to 102 more) based on 3 observational studies with 180 patients [65, 73, 74].

*Neonatal death* estimated 1 fewer event per 1000 patients (95% CI 14 fewer to 216 more) based on 3 observational studies with 227 patients [64, 65, 74].

*NICU admission* estimated 139 fewer events per 1000 patients (95% CI 177 fewer to 97 more) based on 2 observational studies with 120 patients [65, 74].

*Pregnancy loss* estimated 3 fewer events per 1000 patients (95% CI 6 fewer to 2 more) based on 7 observational studies with 6756 patients [62, 64, 65, 71, 73–75].

*Readmission* estimated 42 fewer events per 1000 patients (95% CI 59 fewer to 1 fewer) based on 7 observational studies with 31,446 patients [62, 65, 67–70, 75].

#### Harms and burdens

The harms of laparoscopic cholecystectomy for biliary disease during pregnancy were found to include higher rates of bile leak, IUGR, preeclampsia, preterm birth, and sepsis. The overall magnitude of the effect size was deemed small, and the outcome of preterm birth was noted as being particularly important in this vote. However, the studies included in this question did not differentiate between early and late gestation preterm birth nor spontaneous and indicated preterm birth; therefore, the panel determined that neonatal death and NICU admission were more important outcomes than the heterogeneous preterm birth outcome.

*Bile leak* estimated 1 more event per 1000 patients (95% CI 11 fewer to 66 more) based on 6 observational studies with 23,301 patients [60, 64, 65, 67–69, 76].

*IUGR* estimated 7 more events per 1000 patients (95% CI 23 fewer to 239 more) based on 4 observational studies with 6587 patients [65, 68, 71, 75].

*Preeclampsia* estimated 26 more events per 1000 patients (95% CI 16 fewer to 168 more) based on 4 observational studies with 29,447 patients [65, 67, 69, 75].

*Preterm birth* estimated 58 more events per 1000 patients (95% CI 22 fewer to 207 more) based on 10 observational studies with 39,108 patients [62, 63, 65, 67, 69–71, 73–75].

*Sepsis* estimated 11 more events per 1000 patients (95% CI 2 more to 25 more) based on 3 observational studies with 7677 patients [65, 66, 75].

#### Decision criteria and additional considerations

The panel voted that the balance of these effects probably favors laparoscopic cholecystectomy. It acknowledged that the design of these existing studies meant that patients undergoing cholecystectomy were often sicker than the patients undergoing nonoperative treatment. The panel voted that laparoscopic cholecystectomy during pregnancy would probably be acceptable to key stakeholders and should be feasible to implement. However, the panel also noted that the spectrum of biliary disease is broad. As this guideline will likely be consulted by non-surgeon physicians, it is useful to clearly define these diagnoses: Biliary colic is abdominal pain due to intermittent obstruction of the cystic duct or common bile duct of the biliary tree [77]. Acute cholecystitis is inflammation of the gallbladder that occurs due to occlusion of the cystic duct or impaired emptying of the gallbladder [78].

#### Subgroup: acute cholecystitis only

Recognizing that the severity of biliary disease is a major factor when counseling the patient, the panel completed a subgroup analysis of acute cholecystitis during pregnancy [75]. Nonoperative management consisted of antibiotic therapy. The panel found a large magnitude of desirable effects and a small magnitude of undesirable effects for laparoscopic cholecystectomy. Ultimately, the evidence remained low quality with an overall low certainty. This led to a conditional recommendation for laparoscopic cholecystectomy for acute cholecystitis during pregnancy (*conditional recommendation, low certainty of evidence*).

*Cesarean section* estimated 190 fewer events per 1000 patients (95% CI 199 fewer to 178 fewer) based on 1 observational study with 6390 patients.

*IUGR* estimated 21 fewer events per 1000 patients (95% CI 23 fewer to 16 fewer) based on 1 observational studies with 6390 patients.

*Preeclampsia* estimated 61 fewer events per 1000 patients (95% CI 73 fewer to 46 fewer) based on 1 observational study with 6390 patients.

*Pregnancy loss* estimated 4 fewer events per 1000 patients (95% CI 6 fewer to 1 more) based on 1 observational study with 6390 patients.

*Preterm birth* estimated 63 fewer events per 1000 patients (95% CI 71 fewer to 54 fewer) based on 1 observational study with 6390 patients.

*Readmission* estimated 80 fewer events per 1000 patients (95% CI 93 fewer to 64 fewer) based on 1 observational study with 6390 patients.

*Sepsis* estimated 14 more events per 1000 patients (95% CI 5 more to 26 more) based on 1 observational study with 6390 patients.

#### Subgroup: gestational age/trimester

Operating during late gestation may lead to technical challenges including laparoscopic access and limited working space as well as increased potential for obstetric complications including preterm delivery. Therefore, the panel sought to complete a subgroup analysis by trimester. This evidence was very limited but there were two studies with similar designs that sought to compare surgical and medical management of biliary disease during the 3rd trimester [67, 69]. Both studies utilized large, statewide databases to compare the outcomes of laparoscopic cholecystectomy in women who underwent cholecystectomy in the 3 months before delivery to those who underwent cholecystectomy in the 3 months following delivery. The latter cohort was presumed to be representative of women who had biliary disease managed nonoperatively during their third trimester. Regarding outcomes the panel deemed critical, both studies found higher rates of preterm delivery and one of them also identified higher rates of eclampsia in the prepartum cholecystectomy group. These studies contribute significantly to our understanding of the management of biliary disease during pregnancy. However, there were concerns that a survivorship bias may be present among the cohort who underwent postpartum cholecystectomy; both papers excluded prepartum cholecystectomy patients who had a prior admission for biliary disease, thus removing a portion of the patients who failed nonoperative management from analysis in the postpartum cholecystectomy group. Their methodology also counted women who failed a trial of nonoperative management during the same admission prior to proceeding to cholecystectomy as part of the prepartum cholecystectomy group. Therefore, the prepartum cholecystectomy group may be representative of patients with more severe biliary disease who could not delay cholecystectomy. What these studies show is that if it is known that the patient can delay cholecystectomy until postpartum, they likely should. The problem is identifying which patients truly can wait.

A third paper investigating this question was not included in the meta-analysis as there were no absolute values to be utilized [79]. This paper identified a significantly increased risk of preterm delivery after cholecystectomy in the third trimester relative to the second trimester (AOR 7.2 95% CI 3.09–16.77). While this does not directly answer the question of whether surgical or medical management is the superior treatment for third trimester biliary disease, it does provide further evidence that patients with biliary disease in the third trimester of pregnancy are at an increased risk for preterm delivery. This begs the question whether this risk is ameliorated or worsened by proceeding to operative management.

Nonetheless, the panel suggests that for patients in the third trimester of pregnancy who present with biliary colic, nonoperative management or operative management may be considered (*conditional recommendation, expert opinion*). The decision requires careful consideration of disease severity, chronicity of symptoms, and acuity of presentation. The consequences of preterm delivery, which differ greatly between weeks 26 and 36 for example, should also be taken into consideration.

#### Conclusions

For pregnant patients, the panel suggests laparoscopic cholecystectomy rather than nonoperative treatment for biliary disease (*very low certainty evidence*) and for acute cholecystitis specifically (*low certainty evidence, conditional recommendations*). For biliary colic in the 3rd trimester, the panel suggests either operative or nonoperative management may be considered (*conditional recommendation, expert opinion*).

#### What others are saying

The American College of Gastroenterology (ACG) published guidelines on liver disease and pregnancy in 2016 and recommended, “symptomatic cholecystitis should be managed with early surgical intervention with laparoscopic cholecystectomy (strong recommendation, low level of evidence)” [80]. The basis for this recommendation was the high rate of recurrent symptoms and associated increased risk of spontaneous abortion or preterm labor.

As noted previously, the ACOG recommends treating pregnant patients in need of surgical therapy in the same manner as non-pregnant patients; if the operation is medically necessary, it should be done without delay [23].

### KQ4 should common bile duct exploration (CBDE) for symptomatic choledocholithiasis during pregnancy (any trimester) be used compared to ERCP?

The panel suggests ERCP rather than common bile duct exploration for the treatment of symptomatic choledocholithiasis during pregnancy (any trimester, *conditional recommendation, expert opinion due to low quality of evidence*).

#### Summary of the evidence

Our literature search identified a number of case series reporting on the use of ERCP in pregnant patients [81–89]. The largest was published by Inamdar et al. in 2016 using data from the National Inpatient Sample [83]. Their cohort of 907 patients had 0 maternal mortalities and less than 10 instances each of fetal loss or fetal complications. They did find a higher rate of post-ERCP pancreatitis in pregnant compared to non-pregnant patients (12.13% vs 5.15% *p* < 0.0001).

The existing literature on CBDE during pregnancy is primarily comprised of individual case reports and was therefore excluded from our literature search. In 2023, Lopez-Lopez et al. published a multi-center case series of 8 patients [90]. They reported two low-grade bile leaks managed without intervention but otherwise attributed no postoperative complications for mother or fetus to CBDE.

#### Decision criteria and additional considerations

The literature for this question is incredibly sparse. At present there is more evidence supporting the safety and efficacy of ERCP than CBDE. However, there are benefits to single-stage management with concomitant laparoscopic cholecystectomy, including just one episode of general anesthesia.

Expertise with CBDE is not widespread. In select centers where it is utilized regularly, laparoscopic CBDE can be considered at the surgeon’s discretion. ERCP on the other hand is likely to be a much more accessible option. Regardless of the technique used, timely clearance of the biliary ductal system is the most important consideration.

#### Conclusions

The panel suggests ERCP for the treatment of symptomatic choledocholithiasis during pregnancy. Both modalities appear to have good safety profiles based on the very limited available evidence. CBDE has the benefit of single-stage management when performed at the same time as cholecystectomy but there may be limited expertise with this technique. After the choledocholithiasis is treated, the patient would still be classified as suffering from symptomatic biliary disease during pregnancy and therefore the recommendations and considerations discussed in section “KQ3 should laparoscopic cholecystectomy versus nonoperative treatment be used for the management of biliary disease during pregnancy (any trimester)?” would apply.

#### What others are saying

ACG published guidelines on Liver Disease and Pregnancy in which they state, “ERCP can be performed when indicated for pregnant women presenting with biliary disease that strongly necessitates intervention such as biliary pancreatitis, symptomatic choledocholithiasis, and/or cholangitis. Minimizing fetal exposure to fluoroscopy is imperative (strong recommendation, low level of evidence)” [80]. They acknowledge the importance of minimizing fetal radiation exposure and the risk of post-ERCP pancreatitis but overall noted that biliary pancreatitis, symptomatic choledocholithiasis, and cholangitis all would likely have worse fetal outcomes without intervention.

### KQ5 should laparoscopic intestinal resection for inflammatory bowel disease during pregnancy (any trimester) be used compared to observation/deferred intervention?

The panel suggests that:

Indications for emergent surgical intervention should be the same in the pregnant and non-pregnant IBD patient (*conditional recommendation, expert opinion due to low quality of evidence*).

Pregnant patients requiring emergent surgical treatment of IBD, given the high maternal and fetal complication rates, should undergo an open surgical approach (*conditional recommendation, expert opinion due to low quality of evidence*).

Pregnant patients with active IBD flares should be managed in a multidisciplinary fashion at centers with IBD expertise (*conditional recommendation, expert opinion due to low quality of evidence*).

#### Summary of the evidence

The evidence relevant to this question is limited to small case series that are typically focused on the safety of the different classes of IBD medications in pregnancy [91–97]. The largest case series of surgical treatment during pregnancy was published by Germain et al. [94]. These authors reported on 15 patients with Crohn’s disease who underwent a variety of operations; there was one maternal death, two miscarriages, two medical abortions, and one neonatal death. While this does raise concerns over the poor maternal and fetal outcomes associated with surgical treatment of Crohn’s disease during pregnancy, this is also a very select cohort of patients with a flare so severe as to necessitate operative management.

Puri et al. published a retrospective comparison of 22 women with pan-colitis and 21 with ulcerative colitis up to the splenic flexure prior to pregnancy [97]. The women with pan-colitis suffered higher rates of spontaneous abortion (23% vs 0%), cesarean section for fetal distress (41% vs 10%), and low birth weight (50% vs 14%).

The evidence points to the need to optimize medical management before conception and to the safety of common medical treatments for IBD for their use during pregnancy. The present literature suggests the use of mesalamine, sulfasalazine, steroids, thiopurines, and biologics are relatively safe in pregnancy [98]. However, there are important nuances for the use of some of these medications by trimester and the data for the newer biologics are very limited; the complexity of this clinical decision-making underlines the need for treatment at an expert center with a multidisciplinary team to facilitate discussion of the risks and benefits of the treatment options in a given scenario.

#### Decision criteria and additional considerations

With regard to the surgical treatment of inflammatory bowel disease during pregnancy, there are two scenarios that require consideration: emergent and urgent.

Emergent conditions include the classic surgical emergencies, such as bowel perforation or toxic megacolon. For this scenario, the panel was able to make two conditional recommendations: (1) that the indications for emergent surgical intervention should be the same in pregnant and non-pregnant patients and (2) that these operations should be performed with an open surgical approach. The logic behind the latter recommendation is that the patients are often severely physiologically deranged and performing the operation in the most expedient operation is of the utmost importance.

Urgent conditions span a wide variety of diseases, including but not limited to medically refractory ulcerative colitis, fistulizing Crohn’s disease, and stricturing Crohn’s disease. The management of such conditions will depend not just on the condition and underlying disease process but also patient-specific factors such as pre-conception IBD status and trimester of pregnancy. The implications of bowel rest, total parenteral nutrition, and steroids or biologic usage also differ in pregnant patients. Treatment decisions in such cases must be made according to individual patient factors and after extensive multidisciplinary discussion. As such, specific recommendations for these scenarios are beyond the scope of this guideline. However, it is essential that these be managed by a multidisciplinary team including colorectal surgery, gastroenterology, obstetrics or maternal fetal medicine, and potentially interventional radiology depending on the clinical problem. When this expertise is not available, transfer to an experienced IBD center with the above specialties is strongly advised.

#### Conclusions

Due to poor evidence, the panel made three conditional recommendations based on expert opinion. Indications for emergent surgical intervention should be the same in the pregnant and non-pregnant patient. The GRADE approach limits us to a conditional recommendation due to paucity of evidence but there is no reason to delay an emergent operation in a pregnant patient.

Emergent operations for pregnant patients with IBD should be performed with an open surgical approach. Such patients are often so physiologically deranged that rapid, definitive treatment is the highest priority.

The management of IBD during pregnancy is a complex and relatively uncommon scenario; it therefore requires a multidisciplinary team for optimal management and transfer to an experienced IBD center should be strongly considered.

#### What others are saying

The European Crohn’s and Colitis Organization’s guidelines recommend surgery in pregnant women with IBD for the same indications as in non-pregnant patients (obstruction, perforation, abscess, hemorrhage, or active disease refractory to medical treatment) [98, 99]. They note specifically, “in the severely ill patient, continued illness is a greater risk to the fetus than surgical intervention,” which is in-line with ACOG’s recommendations not to delay or defer non-elective operations in the pregnant population [80].

## Discussion

### What’s new in this guideline

The recommendations made in the 2017 SAGES Guideline on laparoscopy during pregnancy are still applicable. This update provides ten new recommendations based on updated literature regarding the management of appendicitis, biliary disease, choledocholithiasis, and IBD during pregnancy.

All the recommendations were conditional and based on low to very low quality evidence. In fact, six of the nine recommendations were made based on expert opinion because the evidence base is so severely limited. This highlights the need for high-quality studies of these disease processes in the pregnant population.

### Implementation

The panel believes that it is feasible to successfully implement these recommendations into local practice and that the recommendations will be accepted by stakeholders. One potential limitation will be whether sufficient obstetric experience is available when patients present to smaller hospitals.

### Research recommendations

The evidence underlying these recommendations is very weak. The historical exclusion of pregnant patients from RCTs is discussed further in the health equity statement below. RCT evidence in pregnant women should be the gold standard, as it is in other patient populations. At the very least, large, well-designed, prospective cohort studies comparing operative to nonoperative management of these disease processes are needed. These studies must be structured in an organized and reproducible manner. Specifically, they must separate patients by both trimester/gestational age and disease severity (e.g., uncomplicated versus complicated appendicitis, biliary colic versus acute cholecystitis). Comparing outcomes across these groups limits the potential to make meaningful conclusions from the work. Studies also must report both maternal and fetal outcomes for each group as these are essential to the shared decision-making process. Involving obstetric colleagues in the study design will likely significantly improve the quality of the study; the panel noted that many studies in the systematic review reported the outcome of “preterm delivery,” but did not differentiate between early and late preterm delivery and indicated versus spontaneous preterm delivery. A preterm delivery at 26 weeks is drastically different from one at 35 weeks. Grouping all these outcomes together limits their utility. Some studies even reported preterm “labor,” rather than “delivery.” Additionally, outcomes should be analyzed based on an intention to treat basis to properly flag failed nonoperative management and its associated complications.

For situations which are encountered less frequently, such as surgical management of IBD or CBDE during pregnancy, prospective trials would significantly advance our understanding of these disease processes. Multi-center collaboration would likely be required to sufficiently power such studies.

### Health equity statement

The historical exclusion of pregnant women from RCTs limits physicians’ ability to advise them properly of the risks and benefits of treatment options. It is important that efforts be made to include them in trials as long as there are no reasonable safety concerns that would mandate their exclusion [100].

It is well documented that Black women in the United States experience worse pregnancy outcomes than their peers [101–106]. These effects persist even when controlling for variables like maternal age, BMI, socioeconomic status, educational level, and smoking. A recent publication utilizing the National Inpatient Sample found that delay of operative management of appendicitis during pregnancy was associated with higher rates of preterm abortion (OR 1.2), preterm delivery (OR 1.4), antepartum hemorrhage (OR 1.3), and amniotic infection (OR 1.4) [107]. This same study found that Black women were far more likely than other groups to undergo nonoperative management. A recent ACOG policy states “We have an obligation to work to overhaul currently unjust systems that perpetuate unacceptable racial inequities in health outcomes” [108].

### Updating these guidelines

After publication of these guidelines, the SAGES Living Guidelines Taskforce will plan to repeat the literature search at a 3-year interval to identify new evidence. If the updated literature search detects high-quality, new literature, a formal update of these guidelines will be performed. Particular attention will be given to future studies that address the research recommendations proposed in this guideline.

### Limitations of these guidelines

The primary limitation of these guidelines is the very low certainty of evidence on which they are based. The primary issue was limited comparability between groups undergoing operative and nonoperative management as the two groups tended to be quite different at baseline and these studies were prone to survivorship bias. The panel has made multiple research recommendations with the goal of strengthening the evidence base for these KQs.

### Supplementary Information

Below is the link to the electronic supplementary material.

Supplementary file1 (DOCX 13 kb)

Supplementary file2 (DOCX 47 kb)

Supplementary file3 (DOCX 176 kb)

Supplementary file4 (PDF 914 kb)

Supplementary file5 (ZIP 64 kb)

Supplementary file6 (ZIP 91 kb)

Supplementary file7 (ZIP 1876 kb)

## Abbreviations

ACG: The American College of Gastroenterology
ACOG: The American College of Obstetricians and Gynecologists
CBDE: Common bile duct exploration
CI: Confidence interval
EAES: The European Association for Endoscopic Surgery
ERCP: Endoscopic retrograde cholangiopancreatogram
EtD: Evidence to Decision
GRADE: Grading of Recommendations, Assessment, Development, and Evaluation
IBD: Inflammatory bowel disease
IUGR: Intrauterine growth restriction
KQ: Key question
LA: Laparoscopic appendectomy
NICU: Neonatal intensive care unit
NSQIP: National Surgical Quality Improvement Program
OA: Open appendectomy
OR: Odds ratio
PICO: Population, intervention, comparator, outcome
RCT: Randomized controlled trial
RIGHT: Reporting items for practice guidelines in healthcare
SAGES: The Society for American Gastrointestinal and Endoscopic Surgeons
