# Supplementary material for: SAGES guidelines for the use of laparoscopy during pregnancy
Source: Surg Endosc. 2024 May 3;38(6):2947–63. doi: 10.1007/s00464-024-10810-1 (PMC11133165; doi:10.1007/s00464-024-10810-1)
Supplement: Supplementary file 1 — Supplementary file1 (DOCX 13 kb) [file 464_2024_10810_MOESM1_ESM.docx]

Appendix A Author roles

Sunjay S. Kumar contributed to data collection, interpretation of results, and manuscript drafting.

Amelia T. Collings contributed to study design, data collection, and manuscript editing.

Claire Wunker contributed to data collection and manuscript editing.

Dimitrios I. Athanasiadis contributed to data collection and manuscript editing.

Colin G. DeLong contributed to data collection and manuscript editing.
Julie Hong contributed to data collection and manuscript editing.

Mohammed T. Ansari contributed to study design, interpretation of results, and manuscript editing.

Ahmed Abou-Setta contributed to study design, data analysis, and manuscript editing.

Emily Oliver* contributed to study design, data collection, and manuscript editing.

Vincenzo Berghella* contributed to study design and manuscript editing.

Vamsi Alli* contributed to study design, data collection, and manuscript editing.

Imran Hassan* contributed to study design, data collection, and manuscript editing.

Celeste Hollands* contributed to study design, data collection, and manuscript editing.

Patricia Sylla contributed to study design and manuscript editing.

Bethany J. Slater* contributed to study design, data collection, and manuscript editing.

Francesco Palazzo* contributed to study design, data collection, and manuscript drafting.

*Asterisk indicates voting member of expert panel.
