# Supplementary material for: SAGES guidelines for the use of laparoscopy during pregnancy
Source: Surg Endosc. 2024 May 3;38(6):2947–63. doi: 10.1007/s00464-024-10810-1 (PMC11133165; doi:10.1007/s00464-024-10810-1)
Supplement: Supplementary file 2 — Supplementary file2 (DOCX 47 kb) [file 464_2024_10810_MOESM2_ESM.docx]

# Guidelines for the Use of Laparoscopy during Pregnancy

# Literature Searches Strategies

| **Summary**  **Run Dates: August 24-27, 2020; February 21, 2021; June 14, 2021 (KQ3c only); December 13, 2021**  **Librarian: Holly Ann Burt** | | | | | |
| --- | --- | --- | --- | --- | --- |
| **OVERALL**  **SEARCH RESULTS** | Total items identified by database searches | | | 2092 |  |
|  | Total trials identified in searches | | | 194 |  |
|  | Total items located by handsearching trials, databases, and references | | | 47 |  |
|  | Total duplicates identified (across KQs, by HAB, in Endnote, in Covidence) | | | 1,025 |  |
|  | Total unique items screened | | | 920 |  |
| **Databases searched (Coverage)** | | | | | |
| **Systematic Reviews**  Cochrane Library*/ Cochrane (2009-2022) | | **Clinical Trials**  Clinicaltrials.gov/NCBI (2000-2022) | **Medicine**  CINAHL/EBSCO (1937-2022)  Embase/Elsevier (1947-2022)  [PubMed](https://www.ncbi.nlm.nih.gov/pubmed/10653237)/NCBI (1809/1966-2022) | | |
| Databases handsearched for articles published by the trials retrieved by Clinicaltrials.gov and Cochrane: ICTRP (International Clinical Trials Registry Platform)/WHO, Clinicaltrials.gov, PubMed, and Google Scholar | | | | | |
| * Cochrane: Replace [MH xxxxx] with the appropriate MeSH search term. | | | | | |
| Initial and final PICO questions included when different. | | | | | |

## KQ1

| 1. **Should laparoscopic appendectomy versus conservative treatment (abx) be used for acute appendicitis during pregnancy (any trimester)? // Should appendectomy (laparoscopic or open) versus nonoperative treatment (antibiotics) be used for acute appendicitis during pregnancy (any trimester)?** | | |
| --- | --- | --- |
| **Database** | **Final search strategies** | **Results** |
| PubMed  KQ1 | ((pregnancy[mh] OR pregnant[tw] OR pregnancy[tw]) AND ( appendicitis[tw] OR appendicitis[mh]) AND (((appendectomy[mh] OR appendectomy[tw] OR appendectomies[tw] OR appendicectomies[tw] OR appendicectomy[tw]) AND (laparoscopy[mh] OR laparoscopic[tw] OR laparoscopy[tw])) OR nonoperative[tw] OR nonoperatively[tw] OR medical treatment[tw] OR "drug therapy"[sh] OR "administration and dosage"[sh] OR antibiotic[tw] OR antibiotics[tw] OR observation[tiab] OR Anti-Bacterial Agents[mh] OR Anti-Bacterial Agents[pa] OR conservatively[tw] OR conservative[tw]) AND ("Clinical Study"[pt] OR "Comparative Study"[pt] OR "Epidemiologic studies" [mh] OR "Evaluation Study"[pt] OR "Meta-Analysis"[pt] OR "Multicenter Study"[pt] OR "Systematic Review"[pt] OR "Validation Study" [pt] OR randomized[tiab] OR analysis[tiab] OR study[tiab] OR studies [tiab] OR "Practice Guideline"[pt] OR "practice guidelines as topic"[mh] OR guideline[title] OR guidelines[title]) AND english[la]) NOT (Case reports[pt] OR "case report"[tw] OR ("animals"[MH:noexp] NOT "humans"[MH]) OR rat[tiab] OR rats[tiab] OR porcine[tiab] OR ("1905/01/01"[PDAT] : "2015/12/31"[PDAT])) | 49 |
| PubMed | Update run-above with: NOT ("1905/01/01"[PDAT] : "2020/07/31"[PDAT]) | 9 |
| PubMed KQ1 Final | ((pregnancy[mh] OR pregnant[tw] OR pregnancy[tw]) AND ( appendicitis[tw] OR appendicitis[mh]) AND (((appendectomy[mh] OR appendectomy[tw] OR appendectomies[tw] OR appendicectomies[tw] OR appendicectomy[tw]) AND (laparoscopy[mh] OR laparoscopic[tw] OR laparoscopy[tw])) OR nonoperative[tw] OR nonoperatively[tw] OR "medical treatment"[tw] OR "medical management"[tw] OR "drug therapy"[sh] OR "administration and dosage"[sh] OR antibiotic[tw] OR antibiotics[tw] OR observation[tiab] OR Anti-Bacterial Agents[mh] OR Anti-Bacterial Agents[pa] OR conservatively[tw] OR conservative[tw]) AND ("Clinical Study"[pt] OR "Comparative Study"[pt] OR "Epidemiologic studies" [mh] OR "Evaluation Study"[pt] OR "Multicenter Study"[pt] OR "Validation Study" [pt] OR randomized[tiab]) AND english[la]) NOT (Case reports[pt] OR "case report"[tw] OR ("animals"[MH:noexp] NOT "humans"[MH]) OR rat[tiab] OR rats[tiab] OR porcine[tiab] OR ("1905/01/01"[PDAT] : "1989/12/31"[PDAT])) | 102 |
| Embase  KQ1 | ('pregnancy'/exp OR pregnancy) AND ('appendicitis'/exp OR appendicitis) AND ((('laparoscopic surgery'/exp OR 'laparoscopic surgery' OR 'laparoscopy'/exp OR laparoscopy) AND ('appendectomy'/exp OR appendectomy)) OR 'laparoscopic appendectomy'/exp OR 'laparoscopic appendectomy' OR 'antibiotic agent'/exp OR 'antibiotic agent' OR 'conservative treatment'/exp OR 'conservative treatment' OR 'observation'/exp OR observation OR 'drug therapy'/exp OR 'drug therapy') AND [english]/lim AND [2016-2021]/py AND ('practice guideline'/exp OR 'practice guideline' OR [cochrane review]/lim OR [systematic review]/lim OR [meta analysis]/lim OR [clinical study]/lim) NOT ([conference abstract]/lim OR 'case report'/exp OR 'case report' OR 'case study'/exp OR 'case study' OR [animals]/lim) | 69 |
| Embase | Update run-above with: AND [2020-2022]/py | 19 |
| Embase KQ1 Final | ('pregnancy'/exp OR pregnancy) AND ('appendicitis'/exp OR appendicitis) AND ((('laparoscopic surgery'/exp OR 'laparoscopic surgery' OR 'laparoscopy'/exp OR laparoscopy) AND ('appendectomy'/exp OR appendectomy)) OR 'laparoscopic appendectomy'/exp OR 'laparoscopic appendectomy' OR 'antibiotic agent'/exp OR 'antibiotic agent' OR 'conservative treatment'/exp OR 'conservative treatment' OR 'observation'/exp OR observation OR 'drug therapy'/exp OR 'drug therapy') AND [english]/lim AND ('comparative study'/exp OR 'comparative study') NOT ([conference abstract]/lim OR 'case report'/exp OR 'case report' OR 'case study'/exp OR 'case study' OR [animals]/lim OR [1900-1989]/py) | 32 |
| Cochrane  KQ1 | ([MH Pregnant Women] OR (pregnant):ti,ab,kw OR (pregnancy):ti,ab.kw) AND (appendicitis):ti,ab,kw AND [MH Anti-Bacterial Agents] OR [MH Conservative Treatment] OR ("conservative treatment"):ti,ab,kw OR ("non surgical"):ti,ab,kw OR ("non operative"):ti,ab,kw OR ("antibiotics"):ti,ab,kw OR (((appendectomy):ti,ab,kw OR (appendicectomy):ti,ab,kw) AND (("laparoscopic"):ti,ab,kw OR [Laparoscopy]:MH));2016-2020 | 4 |
| Cochrane | Update run-above with: 07012020-123121 | 0 |
| KQ1 Final | Update run-above with: 1990-2021 | 20 |
| CINAHL KQ1 | (pregnancy or pregnant OR (MH "Pregnancy+")) AND appendicitis AND ( conservative treatment or conservative management or non-surgical or non-operative OR (MH "Antibiotics+") OR "antibiotics" OR ((MH "Surgery, Laparoscopic+") AND (MH "Appendectomy")) OR laparoscopic appendectomy) Limiters - Published Date: 20160101-20201231; English Language | 20 |
| CINAHL | Update run-above with: Published Date: 20200801-20210221 | 3 |
| CINAHL KQ1 Final | (pregnancy or pregnant OR (MH "Pregnancy+")) AND appendicitis AND ( conservative treatment or conservative management or non-surgical or non-operative OR (MH "Antibiotics+") OR "antibiotics" OR ((MH "Surgery, Laparoscopic+") AND (MH "Appendectomy")) OR "laparoscopic appendectomy") AND ((MH "Comparative Studies+") OR (MH "Clinical Trials+")) Limiters - Published Date: 19900101-202102211; English Language; Academic Journals | 7 |
| Clinical Trials Q1 | Pregnancy \| Appendicitis OR  Appendicitis AND Pregnancy | 1 |
| **KQ1**  **SEARCH RESULTS** | Total items identified by database searches | 335 |
|  | Additional items located by handsearching | 3 |
|  | Total trials identified in searches | 2 |
|  | Total duplicates identified (by HAB, in Endnote, in Covidence) | 149 |
|  | **Total items screened** | **187** |

## KQ2

| 1. **Should laparoscopic appendectomy versus open appendectomy be used for acute appendicitis during pregnancy (any trimester)?** | | |
| --- | --- | --- |
| **Database** | **Final search strategies** | **Results** |
| PubMed KQ2 | ((pregnancy[mh] OR pregnant[tw] OR pregnancy[tw]) AND ( appendicitis[tw] OR appendicitis[mh]) AND (appendectomy[mh] OR appendectomy[tw] OR appendectomies[tw] OR appendicectomies[tw] OR appendicectomy[tw]) AND ("Clinical Study"[pt] OR "Comparative Study"[pt] OR "Epidemiologic studies" [mh] OR "Evaluation Study"[pt] OR "Meta-Analysis"[pt] OR "Multicenter Study"[pt] OR "Systematic Review"[pt] OR "Validation Study" [pt] OR randomized[tiab] OR analysis[tiab] OR study[tiab] OR studies [tiab] OR "Practice Guideline"[pt] OR "practice guidelines as topic"[mh] OR guideline[title] OR guidelines[title]) AND english[la]) NOT (Case reports[pt] OR "case report"[tw] OR ("animals"[MH:noexp] NOT "humans"[MH]) OR rat[tiab] OR rats[tiab] OR porcine[tiab] OR ("1905/01/01"[PDAT] : "2015/12/31"[PDAT])) | 60 |
| PubMed | Update run-above with: NOT ("1905/01/01"[PDAT] : "2020/07/31"[PDAT]) | 11 |
| PubMed KQ1 Final | ((pregnancy[mh] OR pregnant[tw] OR pregnancy[tw]) AND ( appendicitis[tw] OR appendicitis[mh]) AND (appendectomy[mh] OR appendectomy[tw] OR appendectomies[tw] OR appendicectomies[tw] OR appendicectomy[tw]) AND ("Clinical Study"[pt] OR "Comparative Study"[pt] OR "Epidemiologic studies" [mh] OR "Evaluation Study"[pt] OR "Multicenter Study"[pt] OR "Validation Study" [pt] OR randomized[tiab]) AND english[la]) NOT (Case reports[pt] OR "case report"[tw] OR ("animals"[MH:noexp] NOT "humans"[MH]) OR rat[tiab] OR rats[tiab] OR porcine[tiab] OR ("1905/01/01"[PDAT] : "1989/12/31"[PDAT]) ) | 130 |
| Embase KQ2 | ('pregnancy'/exp OR pregnancy) AND ('appendicitis'/exp OR appendicitis) AND ('appendectomy'/exp OR appendectomy) AND [english]/lim AND [2016-2021]/py AND ('practice guideline'/exp OR 'practice guideline' OR [cochrane review]/lim OR [systematic review]/lim OR [meta analysis]/lim OR [clinical study]/lim) NOT ([conference abstract]/lim OR 'case report'/exp OR 'case report' OR 'case study'/exp OR 'case study' OR [animals]/lim) | 57 |
| Embase | Update run-above with: AND [2020-2022]/py | 18 |
| Embase KQ1 Final | ('pregnancy'/exp OR pregnancy) AND ('appendicitis'/exp OR appendicitis) AND ('appendectomy'/exp OR appendectomy) AND [english]/lim AND ('comparative study'/exp OR 'comparative study') NOT ([conference abstract]/lim OR 'case report'/exp OR 'case report' OR 'case study'/exp OR 'case study' OR [animals]/lim OR [1900-1989]/py) | 30 |
| Cochrane KQ2 | ((MH Pregnant Women) OR (pregnant):ti,ab,kw OR (pregnancy):ti,ab.kw) AND (appendicitis):ti,ab,kw AND ((appendectomy):ti,ab,kw OR (appendicectomy):ti,ab,kw); 2016-2020 | 4 |
| Cochrane | Update run-above with: 07012020-123121 | 0 |
| KQ2 Final | Update run-above with: 1990-2021 | 29 |
| CINAHL KQ2 | (appendicectomy OR appendectomy) AND appendicitis AND (pregnancy or pregnant OR (MH "Pregnancy+")) Limiters - Published Date: 20160101-20201231; English Language ' | 31 |
| CINAHL | Update run-above with: Published Date: 20200801-20210221 | 5 |
| CINAHL KQ1 Final | (appendicectomy OR appendectomy) AND appendicitis AND (pregnancy or pregnant OR (MH "Pregnancy+")) AND (MH "Comparative Studies+") OR (MH "Clinical Trials+") Limiters - Published Date: 19900101-20210221' Academic journals | 12 |
| Clinical Trials Q2 | appendectomy AND Pregnancy \| Appendicitis OR  appendectomy \| Appendicitis AND Pregnancy OR Pregnancy OR Pregnant \| appendectomy | 1 |
| **KQ2**  **SEARCH RESULTS** | Total items identified by database searches | 388 |
|  | Additional items located by handsearching | 2 |
|  | Total trials identified in searches | 2 |
|  | Total duplicates identified (by HAB, in Endnote, in Covidence) | 191 |
|  | **Total items screened** | **197** |

## KQ3

| 1. **Should laparoscopic cholecystectomy for biliary disease during pregnancy (any trimester) be used compared to observation? // Should laparoscopic cholecystectomy versus nonoperative treatment be used for the management of biliary disease during pregnancy (any trimester)?** | | |
| --- | --- | --- |
| NOTE: Three searches on this topic were run and screened; the final results were combined.  a & b run dates: 8/26/20, 2/21/21   1. Should laparoscopic cholecystectomy for acute cholecystitis during pregnancy (any trimester) be used compared to conservative treatment (abx)? 2. Should laparoscopic cholecystectomy for symptomatic cholelithiasis during pregnancy (any trimester) be used compared to observation/deferred intervention?   C. (Final PICO) run dates: 6/14/21, 12/13/21 | | |
| **Database** | **Final search strategies** | **Results** |
| PubMed KQ3a | ((pregnancy[mh] OR pregnant[tw] OR pregnancy[tw]) AND (Cholecystitis[mh] OR cholecystitis[tw]) AND ("medical treatment"[tw] OR ((cholecystectomy[tw] OR Cholecystectomy[mh]) AND (laparoscopic[tw] OR laparoscopy[tw] OR laparoscopy[mh])) OR nonoperative[tw] OR nonoperatively[tw] OR "drug therapy"[sh] OR "administration and dosage"[sh] OR antibiotic[tw] OR antibiotics[tw] OR observation[tiab] OR Anti-Bacterial Agents[mh] OR Anti-Bacterial Agents[pa] OR conservatively[tw] OR conservative[tw]) AND ("Clinical Study"[pt] OR "Comparative Study"[pt] OR "Epidemiologic studies" [mh] OR "Evaluation Study"[pt] OR "Meta-Analysis"[pt] OR "Multicenter Study"[pt] OR "Systematic Review"[pt] OR "Validation Study" [pt] OR randomized[tiab] OR analysis[tiab] OR study[tiab] OR studies [tiab] OR "Practice Guideline"[pt] OR "practice guidelines as topic"[mh] OR guideline[title] OR guidelines[title]) AND english[la]) NOT (Case reports[pt] OR "case report"[tw] OR ("animals"[MH:noexp] NOT "humans"[MH]) OR rat[tiab] OR rats[tiab] OR porcine[tiab] OR ("1905/01/01"[PDAT] : "2015/12/31"[PDAT])) | 12 |
| KQ3a | Update run-above with: NOT ("1905/01/01"[PDAT] : "2020/07/31"[PDAT]) | 3 |
| PubMed KQ3b | ((pregnancy[mh] OR pregnant[tw] OR pregnancy[tw]) AND (Cholelithiasis[mh] OR Cholelithiasis[tw] OR Cholecystolithiasis[tw]OR ((gallstone[tw] OR gallstones[tw]) AND (biliary[tw] OR gallbladder[tw])) OR "biliary colic"[tw]) AND ("Cholecystectomy, Laparoscopic"[mh] OR ((cholecystectomy[tw] OR cholecystectomies[tw] OR Cholecystectomy[mh]) AND (laparoscopic[tw] OR laparoscopy[tw] OR laparoscopy[mh])) OR "Time factors"[mh] OR Observation[tw] OR Deferred[tw] OR Deferring[tw] OR Delayed[tw] OR Delaying[tw] OR postponement [tw] OR postponed [tw] OR "medical treatment"[tw] OR conservatively[tw] OR conservative[tw] OR nonoperative[tw] OR nonoperatively[tw]) AND ("Clinical Study"[pt] OR "Comparative Study"[pt] OR "Epidemiologic studies" [mh] OR "Evaluation Study"[pt] OR "Meta-Analysis"[pt] OR "Multicenter Study"[pt] OR "Systematic Review"[pt] OR "Validation Study" [pt] OR randomized[tiab] OR analysis[tiab] OR study[tiab] OR studies [tiab] OR "Practice Guideline"[pt] OR "practice guidelines as topic"[mh] OR guideline[title] OR guidelines[title]) AND english[la]) NOT (Case reports[pt] OR "case report"[tw] OR ("animals"[MH:noexp] NOT "humans"[MH]) OR rat[tiab] OR rats[tiab] OR porcine[tiab] OR ("1905/01/01"[PDAT] : "2015/12/31"[PDAT])) | 14 |
| KQ3b | Update run-above with: NOT ("1905/01/01"[PDAT] : "2020/07/31"[PDAT]) | 1 |
| PubMed KQ3c | ((pregnancy[mh] OR pregnant[tw] OR pregnancy[tw]) AND (Cholelithiasis[mh] OR Cholelithiasis[tw] OR Cholecystolithiasis[tw] OR ((gallstone[tw] OR gallstones[tw]) AND (biliary[tw] OR gallbladder[tw])) OR Cholecystectomy[tw] OR "biliary colic"[tw] OR "Biliary Tract Diseases"[mh] OR "Cholecystectomy"[mh]) AND (cholecystectomy[tw] OR cholecystectomies[tw] OR "Time factors"[mh] OR Observation[tw] OR Deferred[tw] OR Deferring[tw] OR Delay[tw] OR Delayed[tw] OR Delaying[tw] OR postpone[tw] OR postponement [tw] OR postponed [tw] OR "medical treatment"[tw] OR conservatively[tw] OR conservative[tw] OR nonoperative[tw] OR nonoperatively[tw] OR non-operative[tw] OR non-operatively[tw] OR "drug therapy"[sh] OR "administration and dosage"[sh] OR antibiotic[tw] OR antibiotics[tw] OR "Anti-Bacterial Agents"[mh] OR "Anti-Bacterial Agents"[pa]) AND ("Clinical Study"[pt] OR "Comparative Study"[pt] OR "Epidemiologic studies" [mh] OR "Evaluation Study"[pt] OR "Meta-Analysis"[pt] OR "Multicenter Study"[pt] OR "Systematic Review"[pt] OR "Validation Study" [pt] OR randomized[tiab] OR analysis[tiab] OR study[tiab] OR studies [tiab] OR "Practice Guideline"[pt] OR "practice guidelines as topic"[mh] OR guideline[title] OR guidelines[title])) NOT (Case reports[pt] OR "case report"[tw] OR ("animals"[MH:noexp] NOT "humans"[MH]) OR rat[tiab] OR rats[tiab] OR porcine[tiab] OR ("1905/01/01"[PDAT] : "2015/12/31"[PDAT])) | 95 |
| PubMed KQ3c  Final | ((pregnancy[mh] OR pregnant[tw] OR pregnancy[tw]) AND (Cholelithiasis[mh] OR Cholelithiasis[tw] OR Cholecystolithiasis[tw] OR ((gallstone[tw] OR gallstones[tw]) AND (biliary[tw] OR gallbladder[tw])) OR Cholecystectomy[tw] OR "biliary colic"[tw] OR "Biliary Tract Diseases"[mh] OR "Cholecystectomy"[mh]) AND (cholecystectomy[tw] OR cholecystectomies[tw] OR "Time factors"[mh] OR Observation[tw] OR Deferred[tw] OR Deferring[tw] OR Delay[tw] OR Delayed[tw] OR Delaying[tw] OR postpone[tw] OR postponement [tw] OR postponed [tw] OR "medical treatment"[tw] OR conservatively[tw] OR conservative[tw] OR nonoperative[tw] OR nonoperatively[tw] OR non-operative[tw] OR non-operatively[tw] OR "drug therapy"[sh] OR "administration and dosage"[sh] OR antibiotic[tw] OR antibiotics[tw] OR "Anti-Bacterial Agents"[mh] OR "Anti-Bacterial Agents"[pa]) AND ("Clinical Study"[pt] OR "Comparative Study"[pt] OR "Epidemiologic studies" [mh] OR "Evaluation Study"[pt] OR "Multicenter Study"[pt] OR "Validation Study" [pt] OR randomized[tiab]) AND english[la]) NOT (Case reports[pt] OR "case report"[tw] OR ("animals"[MH:noexp] NOT "humans"[MH]) OR rat[tiab] OR rats[tiab] OR porcine[tiab] OR ("1905/01/01"[PDAT] : "1989/12/31"[PDAT])) | 250 |
| Embase  KQ3a | ('pregnancy'/exp OR pregnancy) AND ('cholecystitis'/exp OR cholecystitis) AND ((('laparoscopic surgery'/exp OR 'laparoscopic surgery' OR 'laparoscopy'/exp OR laparoscopy) AND ('cholecystectomy'/exp OR cholecystectomy)) OR 'laparoscopic cholecystectomy'/exp OR 'laparoscopic cholecystectomy' OR 'antibiotic agent'/exp OR 'antibiotic agent' OR 'conservative treatment'/exp OR 'conservative treatment' OR 'observation'/exp OR observation OR 'drug therapy'/exp OR 'drug therapy') AND [english]/lim AND [2016-2021]/py AND ('practice guideline'/exp OR 'practice guideline' OR [cochrane review]/lim OR [systematic review]/lim OR [meta analysis]/lim OR [clinical study]/lim) NOT ([conference abstract]/lim OR 'case report'/exp OR 'case report' OR 'case study'/exp OR 'case study' OR [animals]/lim) | 28 |
| KQ3a | Update run-above with: AND [2020-2022]/py | 11 |
| Embase  KQ3b | ((pregnancy[mh] OR pregnant[tw] OR pregnancy[tw]) AND (Cholelithiasis[mh] OR Cholelithiasis[tw] OR Cholecystolithiasis[tw]OR ((gallstone[tw] OR gallstones[tw]) AND (biliary[tw] OR gallbladder[tw])) OR "biliary colic"[tw]) AND ("Cholecystectomy, Laparoscopic"[mh] OR ((cholecystectomy[tw] OR cholecystectomies[tw] OR Cholecystectomy[mh]) AND (laparoscopic[tw] OR laparoscopy[tw] OR laparoscopy[mh])) OR "Time factors"[mh] OR Observation[tw] OR Deferred[tw] OR Deferring[tw] OR Delayed[tw] OR Delaying[tw] OR postponement [tw] OR postponed [tw] OR "medical treatment"[tw] OR conservatively[tw] OR conservative[tw] OR nonoperative[tw] OR nonoperatively[tw]) AND ("Clinical Study"[pt] OR "Comparative Study"[pt] OR "Epidemiologic studies" [mh] OR "Evaluation Study"[pt] OR "Meta-Analysis"[pt] OR "Multicenter Study"[pt] OR "Systematic Review"[pt] OR "Validation Study" [pt] OR randomized[tiab] OR analysis[tiab] OR study[tiab] OR studies [tiab] OR "Practice Guideline"[pt] OR "practice guidelines as topic"[mh] OR guideline[title] OR guidelines[title]) AND english[la]) NOT (Case reports[pt] OR "case report"[tw] OR ("animals"[MH:noexp] NOT "humans"[MH]) OR rat[tiab] OR rats[tiab] OR porcine[tiab] OR ("1905/01/01"[PDAT] : "2015/12/31"[PDAT])) | 14 |
| KQ3b | Update run-above with: NOT ("1905/01/01"[PDAT] : "2020/07/31"[PDAT]) | 1 |
| Embase  KQ3c | ('pregnancy'/exp OR pregnancy) AND ('crohn disease'/exp OR 'crohn disease' OR 'ulcerative colitis'/exp OR 'ulcerative colitis' OR 'inflammatory bowel disease'/exp OR 'inflammatory bowel disease') AND ('sphincterotomy'/exp OR sphincterotomy OR 'colon resection'/exp OR 'colon resection' OR 'colostomy'/exp OR colostomy OR 'hemorrhoidectomy'/exp OR hemorrhoidectomy OR 'rectum resection'/exp OR 'rectum resection' OR 'rectum abdominoperineal resection'/exp OR 'rectum abdominoperineal resection' OR 'ileocolectomy'/exp OR ileocolectomy OR 'intestine resection'/exp OR 'intestine resection' OR 'intestine resection'/exp OR 'intestine resection' OR 'proctopexy'/exp OR proctopexy OR 'proctocolectomy'/exp OR proctocolectomy OR 'polypectomy'/exp OR polypectomy OR 'ileal pouch-anal anastomosis'/exp OR 'ileal pouch-anal anastomosis' OR 'j pouch'/exp OR 'j pouch') AND [english]/lim AND [2016-2021]/py AND ('practice guideline'/exp OR 'practice guideline' OR [cochrane review]/lim OR [systematic review]/lim OR [meta analysis]/lim OR [clinical study]/lim) NOT ([conference abstract]/lim OR 'case report'/exp OR 'case report' OR 'case study'/exp OR 'case study' OR [animals]/lim) | 200 |
| Embase  KQ3c  Final | ('pregnancy'/exp OR pregnancy) AND ('cholelithiasis'/exp OR cholelithiasis OR 'biliary colic'/exp OR 'biliary colic' OR (biliary AND ('colic'/exp OR colic)) OR 'cholecystectomy'/exp OR 'cholecystectomy' OR 'biliary tract disease'/exp OR 'biliary tract disease') AND ( 'cholecystectomy'/exp OR cholecystectomy OR cholecystectomies OR deferred OR deferring OR delay OR delayed OR delaying OR postponement OR postponed OR 'observation'/exp OR observation OR 'conservative treatment'/exp OR 'conservative treatment' OR 'time factor'/exp OR 'time factor' OR 'antibiotic agent'/exp OR 'antibiotic agent' OR 'conservative treatment'/exp OR 'conservative treatment' OR 'observation'/exp OR observation OR 'drug therapy'/exp OR 'drug therapy') AND ('comparative study'/exp OR 'comparative study') NOT ([conference abstract]/lim OR 'case report'/exp OR 'case report' OR 'case study'/exp OR 'case study' OR [animals]/lim OR [1900-1989]/py) | 75 |
| Cochrane KQ3a | ([MH Pregnant Women] OR (pregnant):ti,ab,kw OR (pregnancy):ti,ab,kw) AND (cholecystitis):ti,ab,kw AND ([MH Anti-Bacterial Agents] OR [MH Conservative Treatment] OR ("conservative treatment"):ti,ab,kw OR ("non surgical"):ti,ab,kw OR ("non operative"):ti,ab,kw OR (antibiotics):ti,ab,kw OR ((cholecystectomy):ti,ab,kw AND (("laparoscopic"):ti,ab,kw OR [MH Laparoscopy]))) ; 2016-2020 | 7 |
| KQ3a | Update run-above with: 07012020-123121 | 1 |
| Cochrane  KQ3b | ([MH Pregnant Women] OR (pregnant):ti,ab,kw OR (pregnancy):ti,ab,kw) AND ((Cholecystolithiasis):ti,ab,kw OR (Cholelithiasis):ti,ab,kw OR ("Biliary colic"):ti,ab,kw ) AND ([MH Conservative Treatment] OR ("conservative treatment"):ti,ab,kw OR ("non surgical"):ti,ab,kw OR ("non operative"):ti,ab,kw OR [Time Factors]:MH OR ((cholecystectomy):ti,ab,kw AND (("laparoscopic"):ti,ab,kw OR [MH Laparoscopy])));2016-2020 | 30 |
| KQ3b | Update run-above with: 07012020-123121 | 0 |
| Cochrane KQ3c | ('Pregnant Women[MH] OR (pregnant):ti,ab,kw OR (pregnancy):ti,ab,kw) AND (["Inflammatory Bowel Diseases"]:MH OR ["Crohn Disease"]:MH OR ["Colitis, Ulcerative"]:MH OR (IBD):ti,ab,kw ) AND (("Abdominoperineal resection"):ti,ab,kw OR (Colectomy):ti,ab,kw OR ("Colonic Pouches"):ti,ab,kw OR ("Colon resection"):ti,ab,kw OR (Colostomy):ti,ab,kw OR (Hemorrhoidectomy):ti,ab,kw OR (Ileal Pouch):ti,ab,kw OR (Ileocolectomy):ti,ab,kw OR ("Intestinal resection"):ti,ab,kw OR ("J-Pouch"):ti,ab,kw OR (Proctectomy):ti,ab,kw OR (Proctocolectomy):ti,ab,kw OR (Polypectomy):ti,ab,kw OR (Proctopexy):ti,ab,kw OR (Rectopexy):ti,ab,kw OR (rectum resection):ti,ab,kw OR (sphincterotomy):ti,ab,kw OR [Conservative Treatment]: MH OR ("conservative treatment"):ti,ab,kw OR ("non surgical"):ti,ab,kw OR ("non operative"):ti,ab,kw OR ("deferred treatment"):ti,ab,kw OR [Time Factors]:MH );2016-2020 | 93 |
| Cochrane KQ3c  Final | ([MH Pregnant Woment] OR (pregnant):ti OR (pregnancy):ti OR [MH Pregnancy]) AND ( ((Cholecystolithiasis):ti,ab,kw OR (Cholelithiasis):ti,ab,kw OR ("Biliary colic"):ti,ab,kw OR [MH Biliary Tract Diseases] OR [MH Cholecystectomy, Laparoscopic] OR ("Laparoscopic Cholecystectomy"):ti,ab,kw) AND ((cholecystectomy):ti,ab,kw OR [MH cholecystectomy] OR (Observation):ti,ab,kw OR (Deferred):ti,ab,kw OR (Deferring):ti,ab,kw OR (Delay):ti,ab,kw OR (Delayed):ti,ab,kw OR (Delaying):ti,ab,kw OR (postpone):ti,ab,kw OR (postponement):ti,ab,kw OR (postponed):ti,ab,kw OR ("medical treatment"):ti,ab,kw OR [MH Conservative Treatment] OR (conservatively):ti,ab,kw OR ( conservative):ti,ab,kw OR (nonsurgical):ti,ab,kw OR (nonoperative):ti,ab,kw OR (nonoperatively):ti,ab,kw OR ("non-surgical"):ti,ab,kw OR ("non-operative"):ti,ab,kw OR [MH drug therapy] OR ("drug therapy"):ti,ab,kw OR [MH Anti-Bacterial Agents] OR (antibiotic):ti,ab,kw OR (antibiotics):ti,ab,kw OR [MH Time factors])) | 42 |
| CINAHL KQ3a | (pregnancy or pregnant OR (MH "Pregnancy+")) AND cholecystitis AND ( conservative treatment or conservative management or non-surgical or non-operative OR (MH "Antibiotics+") OR "antibiotics" OR ((MH "Surgery, Laparoscopic+") AND (MH "Cholecystectomy+")) OR Laparoscopic Cholecystectomy OR (MH "Cholecystectomy, Laparoscopic")) Limiters - Published Date: 20160101-20201231; English Language | 10 |
| KQ3a | Update run-above with: Published Date: 20200801-20210221 | 0 |
| CINAHL KQ3b | (pregnancy or pregnant OR (MH "Pregnancy+")) AND (Cholecystolithiasis OR (MH "Cholelithiasis") OR Cholelithiasis OR "Biliary colic") AND (conservative treatment OR conservative management OR non-surgical OR non-operative OR delay OR delayed OR deferred OR deferring OR postponed OR postponement OR (MH "Time Factors") OR ((MH "Surgery, Laparoscopic+") AND (MH "Cholecystectomy+")) OR Laparoscopic Cholecystectomy OR (MH "Cholecystectomy, Laparoscopic")); Limiters - Published Date: 20160101-20201231; English Language | 6 |
| KQ3b | Update run-above with: Published Date: 20200801-20210221 | 0 |
| CINAHL KQ3c | (pregnancy or pregnant OR (MH "Pregnancy+")) AND ((MH "Inflammatory Bowel Diseases+") OR (MH "Crohn Disease") OR (MH "Colitis, Ulcerative") OR "IBD") AND (Abdominoperineal resection OR Colectomy OR Colonic Pouches OR Colon resection OR Colostomy OR Hemorrhoidectomy OR Ileal Pouch OR Ileocolectomy OR Intestinal resection OR Intestinal surgery OR J-Pouch OR Proctectomy OR Proctocolectomy OR Polypectomy OR Proctopexy OR Rectopexy OR Rectum resection OR Sphincterotomy OR (MH "Time Factors") OR conservative treatment OR conservative management OR non-surgical OR non-operative OR delay OR delayed OR deferred OR deferring OR postponed OR postponement) Limiters - Published Date: 20160101-20201231; English Language | 48 |
| CINAHL KQ3c  Final | (pregnancy or pregnant OR (MH "Pregnancy+")) AND (Cholecystolithiasis OR (MH "Cholelithiasis") OR Cholelithiasis OR "Biliary colic" OR (MH "Biliary Tract Diseases+") OR (MH "Cholecystectomy") OR cholecystectomy) AND ((MH "Cholecystectomy+") OR Cholecystectomy OR conservative OR conservatively" OR "medical treatment" OR non-surgical OR non-operative OR nonoperative OR nonoperatively OR observation OR delay OR delayed OR deferred OR deferring OR postpone OR postponed OR postponement OR (MH "Time Factors") OR (MH "Antibiotics+") OR "antibiotics" OR (MH "Drug Therapy+")) / Limiters: Published Date: 19900101-20211231; | 10 |
| Clinical Trial KQa | pregnancy \| cholecystitis OR  cholecystitis AND pregnancy | 3 |
| Clinical Trial KQb | Cholelithiasis AND Pregnancy **OR** Pregnancy \| Cholelithiasis **OR**  Pregnancy \| Biliary colic **OR** Biliary colic AND Pregnancy | 3 |
| Clinical Trials KQ3c | pregnancy OR pregnant \| (cholelithiasis OR Biliary colic OR Cholelithiasis OR Choledocholithiasis) OR  (pregnancy OR pregnant) AND cholecystectomy | 13 |
| **KQ3**  **SEARCH RESULTS** | Total items identified by database searches | 976 |
|  | Additional items located by handsearching | 15 |
|  | Total trials identified in searches | 118 |
|  | Total duplicates identified (across strategies, by HAB, in Endnote, in Covidence) | 344 |
|  | **Total items screened** | **529** |

## KQ4

| 1. **Should common bile duct exploration (CBDE) for symptomatic choledocholithiasis during pregnancy (any trimester) be used compared to ERCP? // Should common bile duct exploration (CBDE) versus ERCP be used for symptomatic choledocholithiasis during pregnancy (any trimester)?** | | |
| --- | --- | --- |
| **Database** | **Final search strategies** | **Results** |
| PubMed KQ4 | ((pregnancy[mh] OR pregnant[tw] OR pregnancy[tw]) AND (Cholelithiasis[mh] OR choledocholithiasis[tw] OR ((gallstone[tw] OR gallstones[tw]) AND duct[tw]) OR "duct stones"[tw]) AND (Sphincterotomy[mh] OR Sphincterotomy[tw] OR Sphincterotomies[tw]OR "Cholangiopancreatography, Endoscopic Retrograde"[mh] OR ERCP[tw] OR cholangiopancreatography[tw] OR cholangiopancreatographies[tw] OR "Endoscopic Retrograde"[tw] OR LCBDE[tw] OR CBDE[tw] OR ECBD[tw] OR ("common bile duct"[tw] AND exploration[tw]) OR "stone retrieval"[tw]) AND ("Clinical Study"[pt] OR "Comparative Study"[pt] OR "Epidemiologic studies" [mh] OR "Evaluation Study"[pt] OR "Meta-Analysis"[pt] OR "Multicenter Study"[pt] OR "Systematic Review"[pt] OR "Validation Study" [pt] OR randomized[tiab] OR analysis[tiab] OR study[tiab] OR studies [tiab] OR "Practice Guideline"[pt] OR "practice guidelines as topic"[mh] OR guideline[title] OR guidelines[title]) AND english[la]) NOT (Case reports[pt] OR "case report"[tw] OR ("animals"[MH:noexp] NOT "humans"[MH]) OR rat[tiab] OR rats[tiab] OR porcine[tiab] OR ("1905/01/01"[PDAT] : "2015/12/31"[PDAT])) | 11 |
| PubMed | Update run-above with: NOT ("1905/01/01"[PDAT] : "2020/07/31"[PDAT]) | 3 |
| PubMed KQ4 Final | ((pregnancy[mh] OR pregnant[tw] OR pregnancy[tw]) AND (Cholelithiasis[mh] OR choledocholithiasis[tw] OR ((gallstone[tw] OR gallstones[tw]) AND duct[tw]) OR "duct stones"[tw]) AND (Sphincterotomy[mh] OR Sphincterotomy[tw] OR Sphincterotomies[tw] OR choledochotomy[tw] OR choledochotomies[tw] OR "Cholangiopancreatography, Endoscopic Retrograde"[mh] OR ERCP[tw] OR cholangiopancreatography[tw] OR cholangiopancreatographies[tw] OR "Endoscopic Retrograde"[tw] OR LCBDE[tw] OR CBDE[tw] OR ECBD[tw] OR ("common bile duct"[tw] AND exploration[tw]) OR "stone retrieval"[tw]) AND ("Clinical Study"[pt] OR "Comparative Study"[pt] OR "Epidemiologic studies" [mh] OR "Evaluation Study"[pt] OR "Multicenter Study"[pt] OR "Validation Study" [pt] OR randomized[tiab]) AND english[la]) NOT (Case reports[pt] OR "case report"[tw] OR ("animals"[MH:noexp] NOT "humans"[MH]) OR rat[tiab] OR rats[tiab] OR porcine[tiab] OR ("1905/01/01"[PDAT] : "1989/12/31"[PDAT])) | 36 |
| Embase  KQ4 | ('pregnancy'/exp OR pregnancy) AND ('cholelithiasis'/exp OR cholelithiasis) AND ('choledochotomy'/exp OR choledochotomy OR 'endoscopic retrograde cholangiopancreatography'/exp OR 'endoscopic retrograde cholangiopancreatography' OR 'biliary tract surgery'/exp OR 'biliary tract surgery' OR 'sphincterotomy'/exp OR sphincterotomy OR CBDE OR LCBDE OR ECBD OR ERCP) AND [english]/lim AND [2016-2021]/py AND ('practice guideline'/exp OR 'practice guideline' OR [cochrane review]/lim OR [systematic review]/lim OR [meta analysis]/lim OR [clinical study]/lim) NOT ([conference abstract]/lim OR 'case report'/exp OR 'case report' OR 'case study'/exp OR 'case study' OR [animals]/lim) | 16 |
| Embase | Update run-above with: AND [2020-2022]/py | 12 |
| Embase KQ4 Final | ('pregnancy'/exp OR pregnancy) AND ('cholelithiasis'/exp OR cholelithiasis) AND ('choledochotomy'/exp OR choledochotomy OR 'endoscopic retrograde cholangiopancreatography'/exp OR 'endoscopic retrograde cholangiopancreatography' OR 'biliary tract surgery'/exp OR 'biliary tract surgery' OR 'sphincterotomy'/exp OR sphincterotomy OR CBDE OR LCBDE OR ECBD OR ERCP) AND [english]/lim AND ('comparative study'/exp OR 'comparative study') NOT ([conference abstract]/lim OR 'case report'/exp OR 'case report' OR 'case study'/exp OR 'case study' OR [animals]/lim OR [1900-1989]/py) | 16 |
| Cochrane KQ4 | ((MH Pregnant Women) OR (pregnant):ti,ab,kw OR (pregnancy):ti,ab,kw) AND (Choledocholithiasis):ti,ab,kw AND ((choledochotomy):ti,ab,kw OR (sphincterotomy):ti,ab,kw OR ("Endoscopic Retrograde Cholangiopancreatography"):ti,ab,kw OR (CBDE):ti,ab,kw OR (LCBDE):ti,ab,kw OR (ECBD):ti,ab,kw OR (ERCP):ti,ab,kw OR (("common bile duct "):ti,ab,kw AND (exploration):ti,ab,kw ));2016-2020 | 3 |
| Cochrane | Update run-above with: 07012020-123121 | 0 |
| Cochrane  KQ4 Final | Update run-above with: 1990-2021 | 7 |
| CINAHL KQ4 | '(pregnancy or pregnant OR (MH "Pregnancy+"))AND Choledocholithiasis AND ((MH "Sphincterotomy+") OR Sphincterotomy OR (MH "Cholangiopancreatography, Endoscopic Retrograde") OR CBDE OR LCBDE OR ECBD OR ERCP) Limiters - Published Date: 20160101-20201231; English Language | 7 |
| CINAHL | Update run-above with: Published Date: 20200801-20210221 | 0 |
| CINAHL KQ4 Final | '(pregnancy or pregnant OR (MH "Pregnancy+")) AND Choledocholithiasis AND ((MH "Sphincterotomy+") OR Sphincterotomy OR (MH "Cholangiopancreatography, Endoscopic Retrograde") OR CBDE OR LCBDE OR ECBD OR ERCP) AND (MH "Comparative Studies+") OR (MH "Clinical Trials+"); Limiters - Published Date: 19900101-20210221; English Language | 0 |
| Clinical Trials Q4 | Choledocholithiasis AND Pregnancy OR  Pregnancy \| Choledocholithiasis | 0 |
| **KQ4**  **SEARCH RESULTS** | Total items identified by database searches | 111 |
|  | Additional items located by handsearching | 9 |
|  | Total trials identified in searches | 6 |
|  | Total duplicates identified (by HAB, in Endnote, in Covidence) | 38 |
|  | **Total items screened** | **76** |

## KQ5

| 1. **Should laparoscopic intestinal resection for intestinal disease during pregnancy (any trimester) be used compared to observation/deferred intervention? // Should laparoscopic intestinal resection versus observation/deferred intervention be used for inflammatory bowel disease during pregnancy (any trimester)?** | | |
| --- | --- | --- |
| **Database** | **Final search strategies** | **Results** |
| PubMed KQ5 | ((Pregnancy[mh] OR pregnant [tw] OR Pregnancy[tw]) AND ("Crohn’s disease" [tw] OR "Crohn disease" [tw] OR "Inflammatory Bowel Diseases"[mh] OR IBD[tiab] OR IBD [ot] OR "Ulcerative colitis"[tw] OR " Crohn’s colitis"[tw]) AND (Time factors[mh] OR Observation[tw] OR Deferred[tw] OR Deferring[tw] OR Delayed[tw] OR Delaying[tw] OR postponement [tw] OR postponed [tw] OR "medical treatment"[tw] OR "medical management"[tw] OR conservatively[tw] OR conservative[tw] OR nonoperative[tw] OR nonoperatively[tw] OR Colectomy [mh] OR Colectomy [tw] OR Colectomies [tw]OR Colostomy[mh] OR Colostomy[tw] OR Colostomies[tw] OR Hemorrhoidectomy [mh] OR Hemorrhoidectomy [tw] OR Hemorrhoidectomies[tw] OR Proctectomy [mh] OR Proctectomy [tw] OR Proctectomies [tw] OR Proctocolectomy [tw] OR Proctocolectomies [tw] OR "Abdominoperineal resection" [tw] OR "Abdominoperineal resections" [tw] OR "Rectal Prolapse/surgery"[mh] OR rectopexy[tw] OR rectopexies[tw] OR "Colonic Polyps/surgery"[mh] OR Polypectomy [tw] OR Polypectomies [tw] OR Sphincterotomy[mh] OR Sphincterotomy[tw] OR Sphincterotomies[tw] OR "Colonic Pouches"[mh] OR "Colonic Pouches"[tw] OR "Colonic Pouch"[tw] OR "Ileal Pouch"[tw] OR "Ileal Pouches"[tw] OR "J-Pouch"[tw] OR "J-Pouches"[tw] OR "Ileum/surgery" [mh] OR Ileocolectomy[tw] OR Ileocolectomies[tw] OR "intestinal surgery"[tw] OR "intestinal surgeries"[tw] OR "intestinal resection"[tw] OR "intestinal resections"[tw] OR "colon resection"[tw] OR "colon resections"[tw]) AND ("Clinical Study"[pt] OR "Comparative Study"[pt] OR "Epidemiologic studies" [mh] OR "Evaluation Study"[pt] OR "Meta-Analysis"[pt] OR "Multicenter Study"[pt] OR "Systematic Review"[pt] OR "Validation Study" [pt] OR randomized[tiab] OR analysis[tiab] OR study[tiab] OR studies [tiab] OR "Practice Guideline"[pt] OR "practice guidelines as topic"[mh] OR guideline[title] OR guidelines[title]) AND english[la]) NOT (Case reports[pt] OR "case report"[tw] OR ("animals"[MH:noexp] NOT "humans"[MH]) OR rat[tiab] OR rats[tiab] OR porcine[tiab] OR ("1905/01/01"[PDAT] : "2015/12/31"[PDAT])) | 43 |
| PubMed | Update run-above with: NOT ("1905/01/01"[PDAT] : "2020/07/31"[PDAT]) | 2 |
| PubMed KQ5 Final | ((Pregnancy[mh] OR pregnant [tw] OR Pregnancy[tw]) AND ("Crohn’s disease" [tw] OR "Crohn disease" [tw] OR "Inflammatory Bowel Diseases"[mh] OR IBD[tiab] OR IBD [ot] OR "Ulcerative colitis"[tw] OR " Crohn’s colitis"[tw]) AND (Time factors[mh] OR Observation[tw] OR Deferred[tw] OR Deferring[tw] OR Delayed[tw] OR Delaying[tw] OR postponement [tw] OR postponed [tw] OR "medical treatment"[tw] OR "medical management"[tw] OR conservatively[tw] OR conservative[tw] OR nonoperative[tw] OR nonoperatively[tw] OR Colectomy [mh] OR Colectomy [tw] OR Colectomies [tw]OR Colostomy[mh] OR Colostomy[tw] OR Colostomies[tw] OR Hemorrhoidectomy [mh] OR Hemorrhoidectomy [tw] OR Hemorrhoidectomies[tw] OR Proctectomy [mh] OR Proctectomy [tw] OR Proctectomies [tw] OR Proctocolectomy [tw] OR Proctocolectomies [tw] OR "Abdominoperineal resection" [tw] OR "Abdominoperineal resections" [tw] OR "Rectal Prolapse/surgery"[mh] OR rectopexy[tw] OR rectopexies[tw] OR "Colonic Polyps/surgery"[mh] OR Polypectomy [tw] OR Polypectomies [tw] OR Sphincterotomy[mh] OR Sphincterotomy[tw] OR Sphincterotomies[tw] OR "Colonic Pouches"[mh] OR "Colonic Pouches"[tw] OR "Colonic Pouch"[tw] OR "Ileal Pouch"[tw] OR "Ileal Pouches"[tw] OR "J-Pouch"[tw] OR "J-Pouches"[tw] OR "Ileum/surgery" [mh] OR Ileocolectomy[tw] OR Ileocolectomies[tw] OR "intestinal surgery"[tw] OR "intestinal surgeries"[tw] OR "intestinal resection"[tw] OR "intestinal resections"[tw] OR "colon resection"[tw] OR "colon resections"[tw]) AND ("Clinical Study"[pt] OR "Comparative Study"[pt] OR "Epidemiologic studies" [mh] OR "Evaluation Study"[pt] OR "Multicenter Study"[pt] OR "Validation Study" [pt] OR randomized[tiab]) AND english[la]) NOT (Case reports[pt] OR "case report"[tw] OR ("animals"[MH:noexp] NOT "humans"[MH]) OR rat[tiab] OR rats[tiab] OR porcine[tiab] OR (2016:2021[DP])) | 100 |
| Embase  KQ5 | ('pregnancy'/exp OR pregnancy) AND ('crohn disease'/exp OR 'crohn disease' OR 'ulcerative colitis'/exp OR 'ulcerative colitis' OR 'inflammatory bowel disease'/exp OR 'inflammatory bowel disease') AND ('sphincterotomy'/exp OR sphincterotomy OR 'colon resection'/exp OR 'colon resection' OR 'colostomy'/exp OR colostomy OR 'hemorrhoidectomy'/exp OR hemorrhoidectomy OR 'rectum resection'/exp OR 'rectum resection' OR 'rectum abdominoperineal resection'/exp OR 'rectum abdominoperineal resection' OR 'ileocolectomy'/exp OR ileocolectomy OR 'intestine resection'/exp OR 'intestine resection' OR 'intestine resection'/exp OR 'intestine resection' OR 'proctopexy'/exp OR proctopexy OR 'proctocolectomy'/exp OR proctocolectomy OR 'polypectomy'/exp OR polypectomy OR 'ileal pouch-anal anastomosis'/exp OR 'ileal pouch-anal anastomosis' OR 'j pouch'/exp OR 'j pouch') AND [english]/lim AND [2016-2021]/py AND ('practice guideline'/exp OR 'practice guideline' OR [cochrane review]/lim OR [systematic review]/lim OR [meta analysis]/lim OR [clinical study]/lim) NOT ([conference abstract]/lim OR 'case report'/exp OR 'case report' OR 'case study'/exp OR 'case study' OR [animals]/lim) | 24 |
| Embase | Update run-above with: AND [2020-2022]/py | 4 |
| Embase  KQ5 Final | ('pregnancy'/exp OR pregnancy) AND ('crohn disease'/exp OR 'crohn disease' OR 'ulcerative colitis'/exp OR 'ulcerative colitis' OR 'inflammatory bowel disease'/exp OR 'inflammatory bowel disease') AND ('sphincterotomy'/exp OR sphincterotomy OR 'colon resection'/exp OR 'colon resection' OR 'colostomy'/exp OR colostomy OR 'hemorrhoidectomy'/exp OR hemorrhoidectomy OR 'rectum resection'/exp OR 'rectum resection' OR 'rectum abdominoperineal resection'/exp OR 'rectum abdominoperineal resection' OR 'ileocolectomy'/exp OR ileocolectomy OR 'intestine resection'/exp OR 'intestine resection' OR 'intestine resection'/exp OR 'intestine resection' OR 'proctopexy'/exp OR proctopexy OR 'proctocolectomy'/exp OR proctocolectomy OR 'polypectomy'/exp OR polypectomy OR 'ileal pouch-anal anastomosis'/exp OR 'ileal pouch-anal anastomosis' OR 'j pouch'/exp OR 'j pouch') AND [english]/lim AND ('comparative study'/exp OR 'comparative study') NOT ([conference abstract]/lim OR 'case report'/exp OR 'case report' OR 'case study'/exp OR 'case study' OR [animals]/lim OR [1900-1989]/py) | 11 |
| Cochrane KQ5 | ([MH Pregnant Women] OR (pregnant):ti,ab,kw OR (pregnancy):ti,ab,kw) AND ([MH Inflammatory Bowel Diseases] OR [MH Crohn Disease] OR [MH Colitis, Ulcerative] OR (IBD):ti,ab,kw ) AND (("Abdominoperineal resection"):ti,ab,kw OR (Colectomy):ti,ab,kw OR ("Colonic Pouches"):ti,ab,kw OR ("Colon resection"):ti,ab,kw OR (Colostomy):ti,ab,kw OR (Hemorrhoidectomy):ti,ab,kw OR (Ileal Pouch):ti,ab,kw OR (Ileocolectomy):ti,ab,kw OR ("Intestinal resection"):ti,ab,kw OR ("J-Pouch"):ti,ab,kw OR (Proctectomy):ti,ab,kw OR (Proctocolectomy):ti,ab,kw OR (Polypectomy):ti,ab,kw OR (Proctopexy):ti,ab,kw OR (Rectopexy):ti,ab,kw OR (rectum resection):ti,ab,kw OR (sphincterotomy):ti,ab,kw OR [MH Conservative Treatment] OR ("conservative treatment"):ti,ab,kw OR ("non surgical"):ti,ab,kw OR ("non operative"):ti,ab,kw OR ("deferred treatment"):ti,ab,kw OR [MH Time Factors]);2016-2020 | 6 |
| Cochrane | Update run-above with: 07012020-123121 | 0 |
| Cochrane KQ5 Final | Update run-above with: 1990-2021 | 16 |
| CINAHL KQ5 | (pregnancy or pregnant OR (MH "Pregnancy+")) AND ((MH "Inflammatory Bowel Diseases+") OR (MH "Crohn Disease") OR (MH "Colitis, Ulcerative") OR "IBD") AND (Abdominoperineal resection OR Colectomy OR Colonic Pouches OR Colon resection OR Colostomy OR Hemorrhoidectomy OR Ileal Pouch OR Ileocolectomy OR Intestinal resection OR Intestinal surgery OR J-Pouch OR Proctectomy OR Proctocolectomy OR Polypectomy OR Proctopexy OR Rectopexy OR Rectum resection OR Sphincterotomy OR (MH "Time Factors") OR conservative treatment OR conservative management OR non-surgical OR non-operative OR delay OR delayed OR deferred OR deferring OR postponed OR postponement) Limiters - Published Date: 20160101-20201231; English Language | 11 |
| CINAHL | Update run-above with: Published Date: 20200801-20210221 | 2 |
| CINAHL KQ5 Final | (pregnancy or pregnant OR (MH "Pregnancy+")) AND ((MH "Inflammatory Bowel Diseases+") OR (MH "Crohn Disease") OR (MH "Colitis, Ulcerative") OR "IBD") AND (Abdominoperineal resection OR Colectomy OR Colonic Pouches OR Colon resection OR Colostomy OR Hemorrhoidectomy OR Ileal Pouch OR Ileocolectomy OR Intestinal resection OR Intestinal surgery OR J-Pouch OR Proctectomy OR Proctocolectomy OR Polypectomy OR Proctopexy OR Rectopexy OR Rectum resection OR Sphincterotomy OR (MH "Time Factors") OR conservative treatment OR conservative management OR non-surgical OR non-operative OR delay OR delayed OR deferred OR deferring OR postponed OR postponement) AND (MH "Comparative Studies+") OR (MH "Clinical Trials+"); Limiters - Published Date: 19900101-20210221; English Language | 3 |
| Clinical Trials Q5 | Pregnancy \| (Crohn Colitis OR Crohn Disease OR Inflammatory Bowel Diseases OR IBD) OR  (Crohn Colitis OR Crohn Disease OR Inflammatory Bowel Diseases OR IBD) AND Pregnancy | 60 |
| **KQ5**  **SEARCH RESULTS** | Total items identified by database searches | 282 |
|  | Additional items located by handsearching | 18 |
|  | Total trials identified in searches | 66 |
|  | Total duplicates identified (by HAB, in Endnote, in Covidence) | 82 |
|  | **Total items screened** | **152** |
