# Supplementary material for: SAGES guidelines for the use of laparoscopy during pregnancy
Source: Surg Endosc. 2024 May 3;38(6):2947–63. doi: 10.1007/s00464-024-10810-1 (PMC11133165; doi:10.1007/s00464-024-10810-1)
Supplement: Supplementary file 3 — Supplementary file3 (DOCX 176 kb) [file 464_2024_10810_MOESM3_ESM.docx]

**Guidelines for the Use of Laparoscopy during Pregnancy**

**PRISMA Flow Diagram**

**KQ1**

Additional records identified through handsearching trials and citation searching

ClinicalTrials.gov/NLM (n = 9)

ICTRP/WHO (n = 3)

PubMed/NLM (n=16)

Citation searching (n = 19)

(n = 47 including KQ1 = 3)

Records identified through database searching

ClinicalTrials.gov/NLM (n = 81)

Cochrane Library/Cochrane (n = 262)

CINHAL/EBSCO (n = 175)

Embase/Elsevier (n = 643)

PubMed/NCBI (n = 931)

(n = 2,092) including KQ1 (n = 335)

## Literature Search

Records removed

Duplicates identified during handsearching (n = 23)

Duplicates identified by Endnote (n = 886)

Duplicates identified manually in Endnote (n = 55)

Duplicates identified by Covidence (n = 57)

Duplicates identified manually in Covidence (n = 4)

Trials (n = 194)

(n = 1,219) including KQ1 (n = 149)

Unique records

(n = 920 including KQ1 = 187)

Records not identified as KQ1
(n = 733)

Records excluded
(n = 162)

## Screening

KQ1 Unique records screened
(n = 187)

Full-text articles excluded

(n = 19)

8 Wrong patient population

4 Wrong intervention

3 Wrong comparator

2 Hand search complete

1 Wrong outcomes

1 Wrong study design

Full-text articles assessed for eligibility
(n = 25)

## Eligibility

Studies included in appraisal/ guideline/recommendations
(n = 6)

## Included

Studies included in the quantitative synthesis

(n = 4)

See Literature Searches Summary document for search details.

**Guidelines for the Use of Laparoscopy during Pregnancy**

**PRISMA Flow Diagram**

**KQ2**

Additional records identified through handsearching trials and citation searching

ClinicalTrials.gov/NLM (n = 9)

ICTRP/WHO (n = 3)

PubMed/NLM (n=16)

Citation searching (n = 19)

(n = 47 including KQ2 = 2)

Records identified through database searching

ClinicalTrials.gov/NLM (n = 81)

Cochrane Library/Cochrane (n = 262)

CINHAL/EBSCO (n = 175)

Embase/Elsevier (n = 643)

PubMed/NCBI (n = 931)

(n = 2,092) including KQ2 (n = 388)

## Literature Search

Records removed

Duplicates identified during handsearching (n = 23)

Duplicates identified by Endnote (n = 886)

Duplicates identified manually in Endnote (n = 55)

Duplicates identified by Covidence (n = 57)

Duplicates identified manually in Covidence (n = 4)

Trials (n = 194)

(n = 1,219) including KQ2 (n = 193)

Unique records

(n = 920 including KQ2 = 197)

Records not identified as KQ2
(n = 723)

## Screening

Records excluded
(n = 127)

KQ2 Unique records screened
(n = 197)

Full-text articles excluded

(n = 41)

8 Wrong comparator

7 Citations searched

7 Wrong outcomes

7 Wrong patient population

5 Wrong study design

4 <15pts single arm

3 Wrong intervention

Full-text articles assessed for eligibility
(n = 70)

## Eligibility

Studies included in appraisal/ guideline/recommendations
(n = 29)

## Included

Studies included in the quantitative synthesis

(n = 28)

See Literature Searches Summary document for search details.

**Guidelines for the Use of Laparoscopy during Pregnancy**

**PRISMA Flow Diagram**

**KQ3**

Records identified through database searching

ClinicalTrials.gov/NLM (n = 81)

Cochrane Library/Cochrane (n = 262)

CINHAL/EBSCO (n = 175)

Embase/Elsevier (n = 643)

PubMed/NCBI (n = 931)

(n = 2,092) including KQ3 (n = 976)

(n_a_ = 75 / n_b_ = 75 / n_c_ = 826)

Additional records identified through handsearching trials and citation searching

ClinicalTrials.gov/NLM (n = 9)

ICTRP/WHO (n = 3)

PubMed/NLM (n=16)

Citation searching (n = 19)

(n = 47 including KQ3 = 15)

(n_a_ = 8 / n_b_ = 4 / n_c_ = 3)

## Literature Search

Records removed

Duplicates identified during handsearching (n = 23)

Duplicates identified by Endnote (n = 886)

Duplicates identified manually in Endnote (n = 55)

Duplicates identified by Covidence (n = 57)

Duplicates identified manually in Covidence (n = 4)

Trials (n = 194)

(n = 1,219) including KQ3 (n = 492)

(n_a_ = 31 / n_b_ = 42 / n_c_ = 300; n_a_ and n_b_ are a subset of n_c_)

Unique records

(n = 920 including KQ3 = 529)

Records not identified as KQ3
(n = 391)

Records excluded
(n = 484)

## Screening

KQ3 Unique records screened
(n = 529)

Full-text articles assessed for eligibility
(n = 45)

Full-text articles excluded

7 Wrong patient population

5 SR – Citations searched

4 Wrong study design

3 Wrong outcomes

2 Wrong intervention

1 Wrong comparator

1 Wrong setting

## Eligibility

Studies included in appraisal/ guideline/recommendations
(n = 22)

## Included

Studies included in the quantitative synthesis

(n = 16)

See Literature Searches Summary document for search details.

**Guidelines for the Use of Laparoscopy during Pregnancy**

**PRISMA Flow Diagram**

**KQ4**

Additional records identified through handsearching trials and citation searching

ClinicalTrials.gov/NLM (n = 9)

ICTRP/WHO (n = 3)

PubMed/NLM (n=16)

Citation searching (n = 19)

(n = 47 including KQ4 = 9)

Records identified through database searching

ClinicalTrials.gov/NLM (n = 81)

Cochrane Library/Cochrane (n = 262)

CINHAL/EBSCO (n = 175)

Embase/Elsevier (n = 643)

PubMed/NCBI (n = 931)

(n = 2,092) including KQ4 (n = 111)

## Literature Search

Records removed

Duplicates identified during handsearching (n = 23)

Duplicates identified by Endnote (n = 886)

Duplicates identified manually in Endnote (n = 55)

Duplicates identified by Covidence (n = 57)

Duplicates identified manually in Covidence (n = 4)

Trials (n = 194)

(n = 1,219) including KQ4 (n = 44)

Unique records

(n = 920 including KQ4 = 76)

Records not identified as KQ4
(n = 884)

Records excluded
(n = 40)

## Screening

KQ4 Unique records screened
(n = 76)

Full-text articles excluded

(n = 27)

7 Wrong patient population

5 Wrong intervention

4 Wrong study design

3 Citations searched

3 Wrong setting

2 Wrong comparator

1 Review

1 Non-English review

1 Wrong indication

Full-text articles assessed for eligibility
(n = 36)

## Eligibility

Studies included in appraisal/ guideline/recommendations
(n = 9)

## Included

See Literature Searches Summary document for search details.

**Guidelines for the Use of Laparoscopy during Pregnancy**

**PRISMA Flow Diagram**

**KQ5**

Additional records identified through handsearching trials and citation searching

ClinicalTrials.gov/NLM (n = 9)

ICTRP/WHO (n = 3)

PubMed/NLM (n=16)

Citation searching (n = 19)

(n = 47 including KQ5 = 18)

Records identified through database searching

ClinicalTrials.gov/NLM (n = 81)

Cochrane Library/Cochrane (n = 262)

CINHAL/EBSCO (n = 175)

Embase/Elsevier (n = 643)

PubMed/NCBI (n = 931)

(n = 2,092) including KQ5 (n = 282)

## Literature Search

Records removed

Duplicates identified during handsearching (n = 23)

Duplicates identified by Endnote (n = 886)

Duplicates identified manually in Endnote (n = 55)

Duplicates identified by Covidence (n = 57)

Duplicates identified manually in Covidence (n = 4)

Trials (n = 194)

(n = 1,219) including KQ5 (n = 148)

Unique records

(n = 920 including KQ5 = 152)

Records not identified as KQ5
(n = 768)

Records excluded
(n = 124)

## Screening

KQ5 Unique records screened
(n = 152)

Full-text articles assessed for eligibility
(n = 28)

Full-text articles excluded

(n = 21)

9 Citations searched

6 Wrong intervention

3 Wrong patient population

2 Wrong outcomes

1 Wrong comparator

## Eligibility

Studies included in appraisal/ guideline/recommendations
(n = 7)

## Included

See Literature Searches Summary document for search details.
