# Supplementary material for: SAGES guidelines for the use of laparoscopy during pregnancy
Source: Surg Endosc. 2024 May 3;38(6):2947–63. doi: 10.1007/s00464-024-10810-1 (PMC11133165; doi:10.1007/s00464-024-10810-1)
Supplement: Supplementary file 4 — Supplementary file4 (PDF 914 kb) [file 464_2024_10810_MOESM4_ESM.pdf]

1 KQ1 - Appendectomy vs nonoperative treatment

1.1 C-Section

	Appendectomy		Consverative Tx			Odds Ratio	Risk of Bi
Study or Subgroup	Events	Total	Events	Total	Weight	M-H, Random, 95% CI	A B C D E
Liu 2020	4	20	9	34	100.0%	0.69 [0.18, 2.64]	
**Total (95% CI)**		**20**		**34**	**100.0%**	**0.69 [0.18, 2.64]**	
Total events	4		9				
Heterogeneity: Not applicable							
Test for overall effect: Z = 0.54 (P = 0.59)							

Odds Ratio
M-H, Random, 95% CI

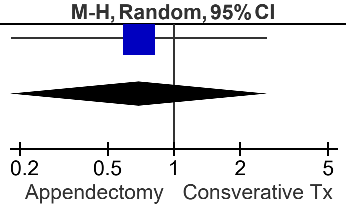

Risk of bias legend
(**A**) Random sequence generation (selection bias)
(**B**) Allocation concealment (selection bias)
(**C**) Blinding of participants and personnel (performance bias)
(**D**) Blinding of outcome assessment (detection bias)
(**E**) Incomplete outcome data (attrition bias)
(**F**) Selective reporting (reporting bias)
(**G**) Other bias

1.2 Delivery during admission

	Appendectomy		Consverative Tx			Odds Ratio	Risk of Bi
Study or Subgroup	Events	Total	Events	Total	Weight	M-H, Random, 95% CI	A B C D E
Joo 2017	0	3	0	17		Not estimable	
**Total (95% CI)**		**3**		**17**		**Not estimable**	
Total events	0		0				
Heterogeneity: Not applicable							
Test for overall effect: Not applicable							

Odds Ratio
M-H, Random, 95% CI

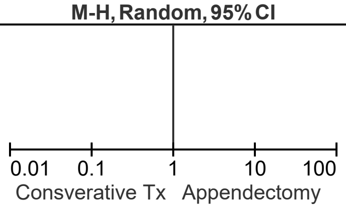

Risk of bias legend
(**A**) Random sequence generation (selection bias)
(**B**) Allocation concealment (selection bias)
(**C**) Blinding of participants and personnel (performance bias)
(**D**) Blinding of outcome assessment (detection bias)
(**E**) Incomplete outcome data (attrition bias)
(**F**) Selective reporting (reporting bias)
(**G**) Other bias

1.3 Preg loss - all

Study or Subgroup	Appendectomy Events	Appendectomy Total	Consverative Tx Events	Consverative Tx Total	Weight	Odds Ratio M-H, Random, 95% CI	Odds Ratio M-H, Random, 95% CI	Risk of Bia A B C D E
Joo 2017	0	3	0	17		Not estimable		
Liu 2020	1	20	1	34	21.2%	1.74 [0.10, 29.39]		
Nakashima 2021	3	56	5	113	78.8%	1.22 [0.28, 5.31]		
**Total (95% CI)**		**79**		**164**	**100.0%**	**1.32 [0.36, 4.85]**		
Total events	4		6					

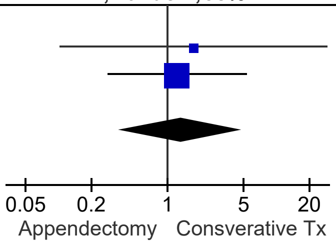

Heterogeneity: Tau² = 0.00; Chi² = 0.05, df = 1 (P = 0.83); I² = 0%
Test for overall effect: Z = 0.41 (P = 0.68)

Risk of bias legend
(**A**) Random sequence generation (selection bias)
(**B**) Allocation concealment (selection bias)
(**C**) Blinding of participants and personnel (performance bias)
(**D**) Blinding of outcome assessment (detection bias)
(**E**) Incomplete outcome data (attrition bias)
(**F**) Selective reporting (reporting bias)
(**G**) Other bias

1.4 Preg loss - <20w

Study or Subgroup	Appendectomy Events	Appendectomy Total	Consverative Tx Events	Consverative Tx Total	Weight	Odds Ratio M-H, Random, 95% CI	Odds Ratio M-H, Random, 95% CI	Risk of Bia A B C D E
Joo 2017	0	3	0	17		Not estimable		
**Total (95% CI)**		**3**		**17**		**Not estimable**		
Total events	0		0					

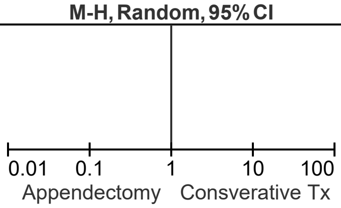

Heterogeneity: Not applicable
Test for overall effect: Not applicable

Risk of bias legend
(**A**) Random sequence generation (selection bias)
(**B**) Allocation concealment (selection bias)
(**C**) Blinding of participants and personnel (performance bias)
(**D**) Blinding of outcome assessment (detection bias)
(**E**) Incomplete outcome data (attrition bias)
(**F**) Selective reporting (reporting bias)
(**G**) Other bias

1.5 Preg loss - >20w

Study or Subgroup	Appendectomy Events	Appendectomy Total	Consverative Tx Events	Consverative Tx Total	Weight	Odds Ratio M-H, Random, 95% CI	Odds Ratio M-H, Random, 95% CI	Risk of Bia A B C D E
Joo 2017	0	3	0	17		Not estimable		
**Total (95% CI)**		**3**		**17**		**Not estimable**		
Total events	0		0					
Heterogeneity: Not applicable								
Test for overall effect: Not applicable								

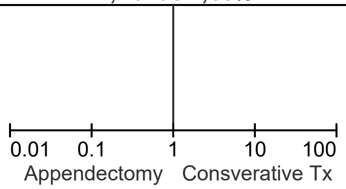

Risk of bias legend
(**A**) Random sequence generation (selection bias)
(**B**) Allocation concealment (selection bias)
(**C**) Blinding of participants and personnel (performance bias)
(**D**) Blinding of outcome assessment (detection bias)
(**E**) Incomplete outcome data (attrition bias)
(**F**) Selective reporting (reporting bias)
(**G**) Other bias

1.6 Preterm delivery

Study or Subgroup	Appendectomy Events	Appendectomy Total	Consverative Tx Events	Consverative Tx Total	Weight	Odds Ratio M-H, Random, 95% CI	Odds Ratio M-H, Random, 95% CI	Risk of Bi A B C D E
Joo 2017	0	3	0	17		Not estimable		
Liu 2020	2	20	3	34	100.0%	1.15 [0.18, 7.53]		
**Total (95% CI)**		**23**		**51**	**100.0%**	**1.15 [0.18, 7.53]**		
Total events	2		3					
Heterogeneity: Not applicable								
Test for overall effect: Z = 0.14 (P = 0.89)								

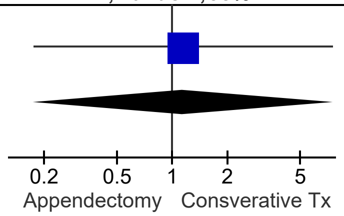

Risk of bias legend
(**A**) Random sequence generation (selection bias)
(**B**) Allocation concealment (selection bias)
(**C**) Blinding of participants and personnel (performance bias)
(**D**) Blinding of outcome assessment (detection bias)
(**E**) Incomplete outcome data (attrition bias)
(**F**) Selective reporting (reporting bias)
(**G**) Other bias

1.7 Readmission

Study or Subgroup	Appendectomy Events	Appendectomy Total	Consverative Tx Events	Consverative Tx Total	Weight	Odds Ratio M-H, Random, 95% CI	Odds Ratio M-H, Random, 95% CI	Risk of Bi A B C D E
Liu 2020	0	20	3	34	100.0%	0.22 [0.01, 4.48]		
**Total (95% CI)**		**20**		**34**	**100.0%**	**0.22 [0.01, 4.48]**		
Total events	0		3					

Heterogeneity: Not applicable
Test for overall effect: Z = 0.99 (P = 0.32)

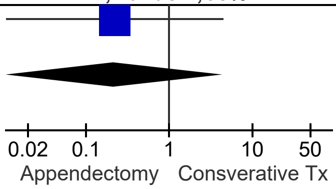

Risk of bias legend
(**A**) Random sequence generation (selection bias)
(**B**) Allocation concealment (selection bias)
(**C**) Blinding of participants and personnel (performance bias)
(**D**) Blinding of outcome assessment (detection bias)
(**E**) Incomplete outcome data (attrition bias)
(**F**) Selective reporting (reporting bias)
(**G**) Other bias

1.8 Sepsis

Study or Subgroup	Appendectomy Events	Appendectomy Total	Consverative Tx Events	Consverative Tx Total	Weight	Odds Ratio M-H, Random, 95% CI	Odds Ratio M-H, Random, 95% CI	Risk of Bi A B C D E
Abbasi 2014	10	6701	4	413	100.0%	0.15 [0.05, 0.49]		
**Total (95% CI)**		**6701**		**413**	**100.0%**	**0.15 [0.05, 0.49]**		
Total events	10		4					

Heterogeneity: Not applicable
Test for overall effect: Z = 3.16 (P = 0.002)

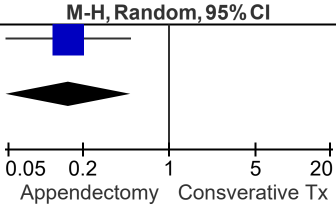

Risk of bias legend
(**A**) Random sequence generation (selection bias)
(**B**) Allocation concealment (selection bias)
(**C**) Blinding of participants and personnel (performance bias)
(**D**) Blinding of outcome assessment (detection bias)
(**E**) Incomplete outcome data (attrition bias)
(**F**) Selective reporting (reporting bias)
(**G**) Other bias

2.1 C-Section

Study or Subgroup	Laparoscopy Events	Laparoscopy Total	Open Events	Open Total	Weight	Odds Ratio M-H, Random, 95% CI
Amin 2018	13	18	9	13	1.5%	1.16 [0.24, 5.53]
Cai 2020	5	12	20	36	2.1%	0.57 [0.15, 2.14]
Carver 2005	2	17	3	11	0.9%	0.36 [0.05, 2.59]
Cheng 2015	52	128	258	653	24.2%	1.05 [0.71, 1.54]
Chung 2013	6	22	14	39	2.8%	0.67 [0.21, 2.10]
Karaman 2016	4	12	11	36	1.9%	1.14 [0.28, 4.58]
Kirshtein 2009	6	23	3	19	1.5%	1.88 [0.40, 8.82]
Peled 2014	7	26	18	59	3.4%	0.84 [0.30, 2.35]
Sadot 2010	10	40	3	16	1.7%	1.44 [0.34, 6.13]
Tumati 2020	231	547	181	459	56.4%	1.12 [0.87, 1.45]
Yoo 2016	13	24	18	56	3.8%	2.49 [0.94, 6.64]
**Total (95% CI)**		**869**		**1397**	**100.0%**	**1.10 [0.91, 1.33]**
Total events	349		538			

Heterogeneity: Tau² = 0.00; Chi² = 6.55, df = 10 (P = 0.77); I² = 0%
Test for overall effect: Z = 0.96 (P = 0.34)

Odds Ratio M-H, Random, 95% CI

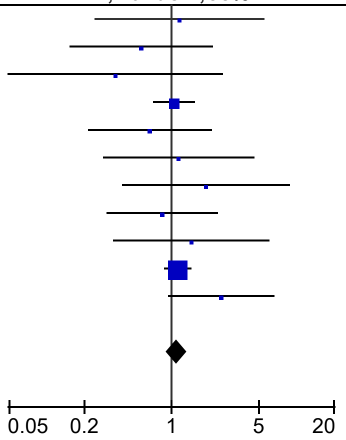

Risk of Bias
A B C D E F G

Risk of bias legend
(**A**) Random sequence generation (selection bias)
(**B**) Allocation concealment (selection bias)
(**C**) Blinding of participants and personnel (performance bias)
(**D**) Blinding of outcome assessment (detection bias)
(**E**) Incomplete outcome data (attrition bias)
(**F**) Selective reporting (reporting bias)
(**G**) Other bias

2.2 Delivery

Study or Subgroup	Laparoscopy Events	Laparoscopy Total	Open Events	Open Total	Weight	Odds Ratio M-H, Random, 95% CI
Aggenbach 2015	0	5	1	16	100.0%	0.94 [0.03, 26.65]
Amin 2018	0	18	0	13		Not estimable
**Total (95% CI)**		**23**		**29**	**100.0%**	**0.94 [0.03, 26.65]**
Total events	0		1			

Heterogeneity: Not applicable
Test for overall effect: Z = 0.04 (P = 0.97)

Odds Ratio M-H, Random, 95% CI

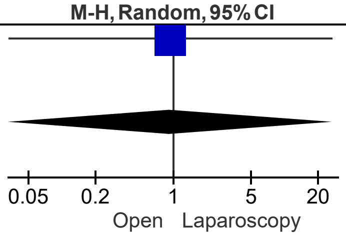

Risk of Bias
A B C D E F G

Risk of bias legend
(**A**) Random sequence generation (selection bias)
(**B**) Allocation concealment (selection bias)
(**C**) Blinding of participants and personnel (performance bias)
(**D**) Blinding of outcome assessment (detection bias)
(**E**) Incomplete outcome data (attrition bias)
(**F**) Selective reporting (reporting bias)
(**G**) Other bias

2.3 Neonatal death

Study or Subgroup	Laparoscopy Events	Laparoscopy Total	Open Events	Open Total	Weight	Odds Ratio M-H, Random, 95% CI
Amin 2018	0	18	0	13		Not estimable
Kapan 2013	0	7	0	10		Not estimable
Khan 2012	0	52	0	66		Not estimable
Laustsen 2016	0	19	0	25		Not estimable
Lyass 2001	0	11	0	11		Not estimable
Peled 2014	0	26	0	59		Not estimable
**Total (95% CI)**		**133**		**184**		**Not estimable**
Total events	0		0			

Heterogeneity: Not applicable
Test for overall effect: Not applicable

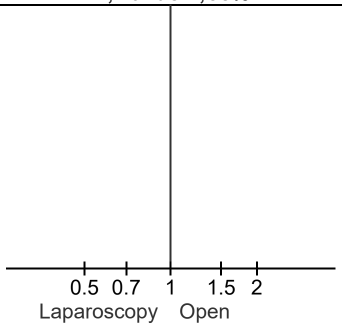

Risk of bias legend
(**A**) Random sequence generation (selection bias)
(**B**) Allocation concealment (selection bias)
(**C**) Blinding of participants and personnel (performance bias)
(**D**) Blinding of outcome assessment (detection bias)
(**E**) Incomplete outcome data (attrition bias)
(**F**) Selective reporting (reporting bias)
(**G**) Other bias

2.4 NICU

Study or Subgroup	Laparoscopy Events	Laparoscopy Total	Open Events	Open Total	Weight	Odds Ratio M-H, Random, 95% CI
Amin 2018	1	18	0	13	100.0%	2.31 [0.09, 61.41]
**Total (95% CI)**		**18**		**13**	**100.0%**	**2.31 [0.09, 61.41]**
Total events	1		0			

Heterogeneity: Not applicable
Test for overall effect: Z = 0.50 (P = 0.62)

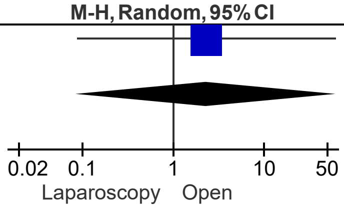

Risk of bias legend
(**A**) Random sequence generation (selection bias)
(**B**) Allocation concealment (selection bias)
(**C**) Blinding of participants and personnel (performance bias)
(**D**) Blinding of outcome assessment (detection bias)
(**E**) Incomplete outcome data (attrition bias)
(**F**) Selective reporting (reporting bias)
(**G**) Other bias

2.5 Preg loss - all

Study or Subgroup	Laparoscopy Events	Laparoscopy Total	Open Events	Open Total	Weight	Odds Ratio M-H, Random, 95% CI	Risk of Bias A B C D E F G
Aggenbach 2015	0	5	0	16		Not estimable	
Alkatary 2017	0	9	0	12		Not estimable	
Amin 2018	0	18	0	13		Not estimable	
Arer 2016	0	16	0	4		Not estimable	
Cai 2020	1	12	1	36	1.4%	3.18 [0.18, 55.19]	
Carver 2005	2	17	0	11	1.1%	3.71 [0.16, 84.92]	
Cheng 2015	7	128	37	653	16.0%	0.96 [0.42, 2.21]	
Chung 2013	0	22	0	39		Not estimable	
Eom 2012	0	15	0	28		Not estimable	
Gök 2018	0	18	1	39	1.0%	0.69 [0.03, 17.86]	
Kapan 2013	0	7	0	10		Not estimable	
Karaman 2016	1	12	1	36	1.4%	3.18 [0.18, 55.19]	
Khan 2012	0	52	0	66		Not estimable	
Kirshtein 2009	1	23	1	19	1.4%	0.82 [0.05, 14.02]	
Kozan 2020	3	14	0	7	1.1%	4.57 [0.21, 101.61]	
Laustsen 2016	0	19	0	25		Not estimable	
Lyass 2001	0	11	0	11		Not estimable	
Maimaiti 2017	0	7	1	19	1.0%	0.82 [0.03, 22.54]	
McGory 2007	31	454	88	2679	62.0%	2.16 [1.41, 3.29]	
Nakashima 2021	2	14	1	42	1.8%	6.83 [0.57, 82.02]	
Peled 2014	1	26	0	59	1.1%	7.00 [0.28, 177.68]	
Rollins 2004	0	28	0	2		Not estimable	
Sadot 2010	1	41	0	16	1.0%	1.22 [0.05, 31.57]	
Segev 2016	2	50	2	42	2.7%	0.83 [0.11, 6.18]	
Tumati 2020	3	547	0	441	1.3%	5.68 [0.29, 110.17]	
Winter 2017	7	125	0	93	1.3%	11.84 [0.67, 209.90]	
Yoo 2017	3	24	4	56	4.4%	1.86 [0.38, 9.02]	
**Total (95% CI)**		**1714**		**4474**	**100.0%**	**1.93 [1.39, 2.70]**	
Total events	65		137				

Heterogeneity: Tau² = 0.00; Chi² = 9.06, df = 15 (P = 0.87); I² = 0%

Test for overall effect: Z = 3.89 (P < 0.0001)

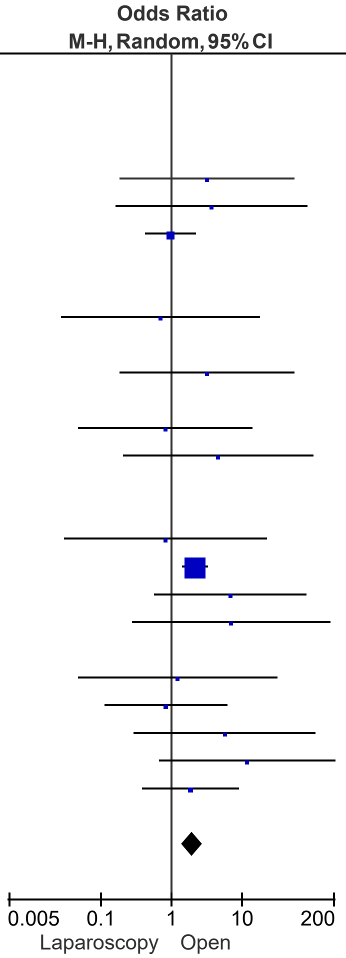

Risk of bias legend

(**A**) Random sequence generation (selection bias)

(**B**) Allocation concealment (selection bias)

(**C**) Blinding of participants and personnel (performance bias)

(**D**) Blinding of outcome assessment (detection bias)

(**E**) Incomplete outcome data (attrition bias)

(**F**) Selective reporting (reporting bias)

(**G**) Other bias

2.6 Preg loss - <20w

Study or Subgroup	Laparoscopy Events	Laparoscopy Total	Open Events	Open Total	Weight	Odds Ratio M-H, Random, 95% CI	Risk of Bias A B C D E F G
Aggenbach 2015	0	5	0	16		Not estimable	
Alkatary 2017	0	9	0	12		Not estimable	
Carver 2005	2	17	0	11	16.1%	3.71 [0.16, 84.92]	
Chung 2013	0	22	0	39		Not estimable	
Kapan 2013	0	7	0	10		Not estimable	
Karaman 2016	1	12	0	36	14.8%	9.52 [0.36, 250.16]	
Kirshtein 2009	1	23	1	19	19.5%	0.82 [0.05, 14.02]	
Kozan 2020	3	14	0	7	16.4%	4.57 [0.21, 101.61]	
Lyass 2001	0	11	0	11		Not estimable	
Maimaiti 2017	0	7	1	19	14.4%	0.82 [0.03, 22.54]	
Winter 2017	6	125	0	93	18.9%	10.17 [0.57, 182.86]	
**Total (95% CI)**		**252**		**273**	**100.0%**	**3.20 [0.91, 11.22]**	
Total events	13		2				

Heterogeneity: Tau² = 0.00; Chi² = 2.68, df = 5 (P = 0.75); I² = 0%
Test for overall effect: Z = 1.82 (P = 0.07)

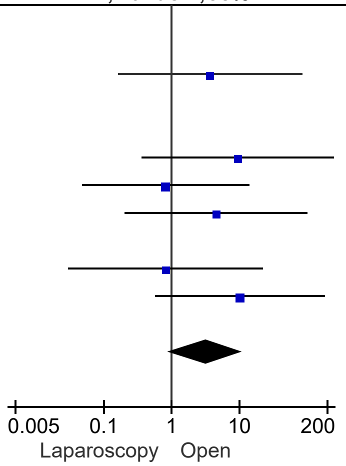

Risk of bias legend
(**A**) Random sequence generation (selection bias)
(**B**) Allocation concealment (selection bias)
(**C**) Blinding of participants and personnel (performance bias)
(**D**) Blinding of outcome assessment (detection bias)
(**E**) Incomplete outcome data (attrition bias)
(**F**) Selective reporting (reporting bias)
(**G**) Other bias

2.7 Preg loss - >20w

	Laparoscopy		Open			Odds Ratio
**Study or Subgroup**	**Events**	**Total**	**Events**	**Total**	**Weight**	**M-H, Random, 95% CI**
Aggenbach 2015	0	5	0	16		Not estimable
Alkatary 2017	0	9	0	12		Not estimable
Chung 2013	0	22	0	39		Not estimable
Kapan 2013	0	7	0	10		Not estimable
Karaman 2016	0	12	1	36	49.2%	0.95 [0.04, 24.78]
Kozan 2020	0	14	0	7		Not estimable
Lyass 2001	0	11	0	11		Not estimable
Winter 2017	1	125	0	93	50.8%	2.25 [0.09, 55.93]
**Total (95% CI)**		**205**		**224**	**100.0%**	**1.47 [0.15, 14.52]**
Total events	1		1			

Heterogeneity: Tau² = 0.00; Chi² = 0.14, df = 1 (P = 0.71); I² = 0%

Test for overall effect: Z = 0.33 (P = 0.74)

Odds Ratio
M-H, Random, 95% CI

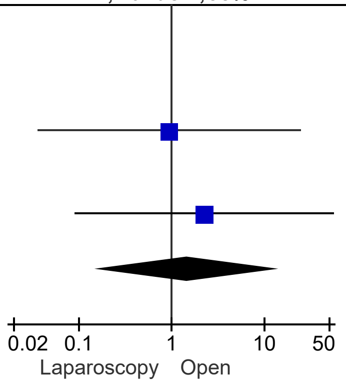

Risk of Bias
A B C D E F G

Risk of bias legend
(**A**) Random sequence generation (selection bias)
(**B**) Allocation concealment (selection bias)
(**C**) Blinding of participants and personnel (performance bias)
(**D**) Blinding of outcome assessment (detection bias)
(**E**) Incomplete outcome data (attrition bias)
(**F**) Selective reporting (reporting bias)
(**G**) Other bias

2.8 Preterm delivery

Study or Subgroup	Laparoscopy Events	Laparoscopy Total	Open Events	Open Total	Weight	Odds Ratio M-H, Random, 95% CI
Alkatary 2017	0	9	1	12	1.7%	0.40 [0.01, 11.09]
Amin 2018	1	18	0	13	1.7%	2.31 [0.09, 61.41]
Cai 2020	1	12	1	36	2.2%	3.18 [0.18, 55.19]
Cheng 2015	7	128	74	653	10.9%	0.45 [0.20, 1.01]
Chung 2013	2	22	4	39	4.6%	0.88 [0.15, 5.21]
Eom 2012	0	15	3	28	1.9%	0.24 [0.01, 4.86]
Gök 2018	1	18	2	39	2.8%	1.09 [0.09, 12.84]
Kapan 2013	0	7	0	10		Not estimable
Karaman 2016	3	12	9	36	5.8%	1.00 [0.22, 4.52]
Khan 2012	4	52	6	66	6.8%	0.83 [0.22, 3.12]
Kozan 2020	2	14	1	7	2.6%	1.00 [0.07, 13.37]
Laustsen 2016	3	19	2	25	4.2%	2.16 [0.32, 14.41]
Lyass 2001	0	11	0	11		Not estimable
Maimaiti 2017	2	7	1	19	2.5%	7.20 [0.54, 96.64]
McGory 2007	1	454	216	2679	4.0%	0.03 [0.00, 0.18]
Peled 2014	5	26	14	59	8.0%	0.77 [0.24, 2.40]
Sadot 2010	12	41	3	16	6.2%	1.79 [0.43, 7.45]
Segev 2016	5	50	3	42	5.9%	1.44 [0.32, 6.44]
Tumati 2020	51	547	42	441	14.5%	0.98 [0.64, 1.50]
Winter 2017	8	117	8	93	9.0%	0.78 [0.28, 2.16]
Yoo 2018	2	24	4	56	4.7%	1.18 [0.20, 6.93]
**Total (95% CI)**		**1603**		**4380**	**100.0%**	**0.86 [0.55, 1.35]**
Total events	110		394			

Heterogeneity: Tau² = 0.32; Chi² = 30.10, df = 18 (P = 0.04); I² = 40%
Test for overall effect: Z = 0.66 (P = 0.51)

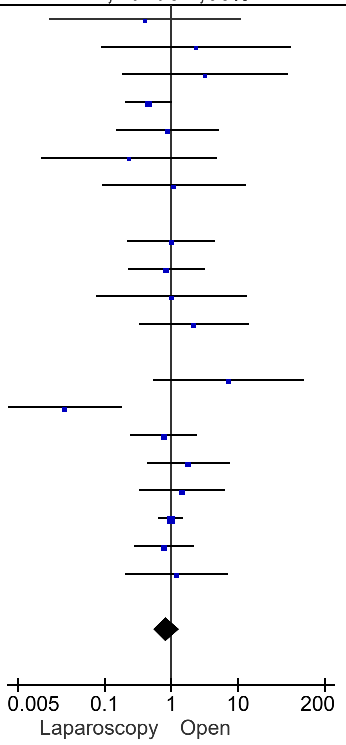

Risk of Bias
A B C D E F G

Risk of bias legend
(**A**) Random sequence generation (selection bias)
(**B**) Allocation concealment (selection bias)
(**C**) Blinding of participants and personnel (performance bias)
(**D**) Blinding of outcome assessment (detection bias)
(**E**) Incomplete outcome data (attrition bias)
(**F**) Selective reporting (reporting bias)
(**G**) Other bias

2.9 Readmission

Study or Subgroup	Laparoscopy Events	Laparoscopy Total	Open Events	Open Total	Weight	Odds Ratio M-H, Random, 95% CI
Amin 2018	1	18	0	13	3.9%	2.31 [0.09, 61.41]
Gök 2018	1	18	2	39	6.9%	1.09 [0.09, 12.84]
Tumati 2020	16	547	18	459	89.2%	0.74 [0.37, 1.46]
**Total (95% CI)**		**583**		**511**	**100.0%**	**0.79 [0.41, 1.51]**
Total events	18		20			

Heterogeneity: Tau² = 0.00; Chi² = 0.52, df = 2 (P = 0.77); I² = 0%
Test for overall effect: Z = 0.70 (P = 0.48)

Odds Ratio M-H, Random, 95% CI

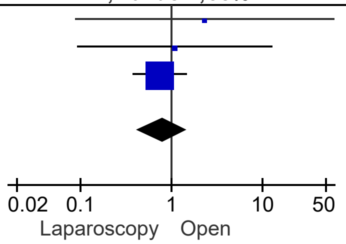

Risk of Bias: A B C D E F G

Risk of bias legend
(**A**) Random sequence generation (selection bias)
(**B**) Allocation concealment (selection bias)
(**C**) Blinding of participants and personnel (performance bias)
(**D**) Blinding of outcome assessment (detection bias)
(**E**) Incomplete outcome data (attrition bias)
(**F**) Selective reporting (reporting bias)
(**G**) Other bias

2.10 Sepsis

Study or Subgroup	Laparoscopy Events	Laparoscopy Total	Open Events	Open Total	Weight	Odds Ratio M-H, Random, 95% CI
Cox 2016	7	894	5	441	87.4%	0.69 [0.22, 2.18]
Tumati 2020	0	547	2	459	12.6%	0.17 [0.01, 3.49]
**Total (95% CI)**		**1441**		**900**	**100.0%**	**0.58 [0.20, 1.69]**
Total events	7		7			

Heterogeneity: Tau² = 0.00; Chi² = 0.74, df = 1 (P = 0.39); I² = 0%
Test for overall effect: Z = 1.00 (P = 0.32)

Odds Ratio M-H, Random, 95% CI

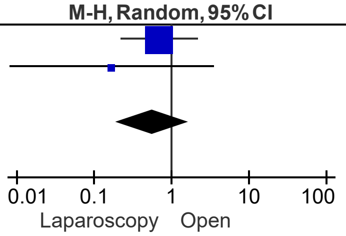

Risk of Bias: A B C D E F G

Risk of bias legend
(**A**) Random sequence generation (selection bias)
(**B**) Allocation concealment (selection bias)
(**C**) Blinding of participants and personnel (performance bias)
(**D**) Blinding of outcome assessment (detection bias)
(**E**) Incomplete outcome data (attrition bias)
(**F**) Selective reporting (reporting bias)
(**G**) Other bias

3 KQ10 - Cholecystectomy vs. nonoperative Tx of biliary disease

3.1 Bile leak

	Cholecystectomy		Medical Tx			Odds Ratio	Odds Ratio
Study or Subgroup	Events	Total	Events	Total	Weight	M-H, Random, 95% CI	M-H, Random, 95% CI
ChiappettaPorras 2009	0	54	0	53		Not estimable	
Davis 1995	0	19	0	23		Not estimable	
Fong 2019	3	403	165	17490	52.9%	0.79 [0.25, 2.48]	
Hong 2020	0	82	129	5040	26.0%	0.23 [0.01, 3.73]	
Othman 2012	0	31	0	50		Not estimable	
Veerappan 2013	1	10	0	46	21.1%	14.68 [0.55, 388.61]	
**Total (95% CI)**		**599**		**22702**	**100.0%**	**1.06 [0.17, 6.53]**	
Total events	4		294				

Heterogeneity: $Tau^2 = 1.29$; $Chi^2 = 3.82$, df = 2 (P = 0.15); $I^2 = 48\%$
Test for overall effect: Z = 0.06 (P = 0.95)

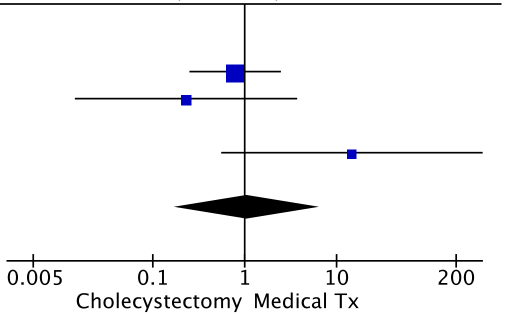

(**A**) Random sequence generation (selection bias)
(**B**) Allocation concealment (selection bias)
(**C**) Blinding of participants and personnel (performance bias)
(**D**) Blinding of outcome assessment (detection bias)
(**E**) Incomplete outcome data (attrition bias)
(**F**) Selective reporting (reporting bias)
(**G**) Other bias

3.2 C-Section

Study or Subgroup	Cholecystectomy Events	Cholecystectomy Total	Medical Tx Events	Medical Tx Total	Weight	Odds Ratio M-H, Random, 95% CI
**3.2.1 Cholecystitis only**						
Rios-Diaz 2020	147	2440	984	3950	17.6%	0.19 [0.16, 0.23]
**Subtotal (95% CI)**		**2440**		**3950**	**17.6%**	**0.19 [0.16, 0.23]**
Total events	147		984			
Heterogeneity: Not applicable						
Test for overall effect: Z = 17.73 (P < 0.00001)						
**3.2.2 Others**						
Davis 1995	1	19	0	23	5.2%	3.81 [0.15, 99.08]
Fong 2019	173	403	6997	17490	17.5%	1.13 [0.92, 1.38]
Hedström 2017	0	34	4	62	6.0%	0.19 [0.01, 3.61]
Hong 2020	41	82	2221	5040	17.0%	1.27 [0.82, 1.96]
Ibiebele 2017	79	239	481	1643	17.4%	1.19 [0.89, 1.59]
Jorge 2015	0	4	6	49	5.7%	0.74 [0.04, 15.48]
Kolbeinsson 2016	1	15	0	45	5.2%	9.41 [0.36, 243.94]
Lu 2004	1	13	6	65	8.4%	0.82 [0.09, 7.44]
**Subtotal (95% CI)**		**809**		**24417**	**82.4%**	**1.16 [1.00, 1.35]**
Total events	296		9715			
Heterogeneity: $Tau^2 = 0.00; Chi^2 = 4.01, df = 7 (P = 0.78); I^2 = 0\%$						
Test for overall effect: Z = 1.93 (P = 0.05)						
**Total (95% CI)**		**3249**		**28367**	**100.0%**	**0.87 [0.36, 2.10]**
Total events	443		10699			
Heterogeneity: $Tau^2 = 1.16; Chi^2 = 228.61, df = 8 (P < 0.00001); I^2 = 97\%$						
Test for overall effect: Z = 0.32 (P = 0.75)						
Test for subgroup differences: $Chi^2 = 219.57, df = 1 (P < 0.00001), I^2 = 99.5\%$						

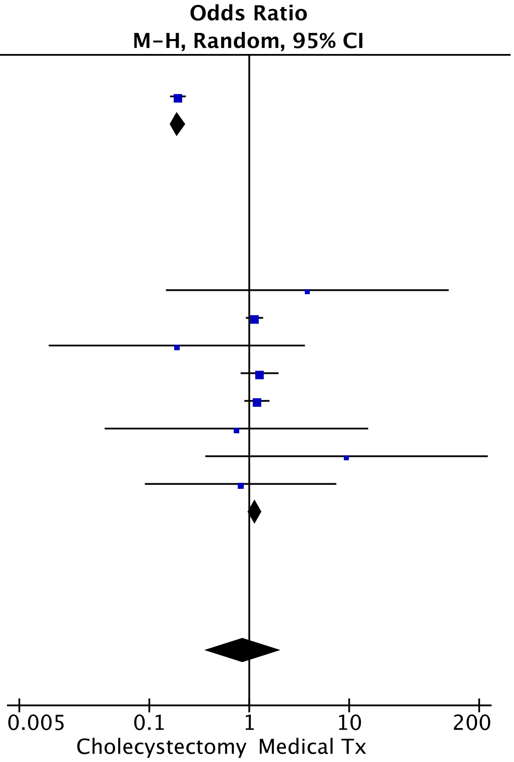

(**A**) Random sequence generation (selection bias)
(**B**) Allocation concealment (selection bias)
(**C**) Blinding of participants and personnel (performance bias)
(**D**) Blinding of outcome assessment (detection bias)
(**E**) Incomplete outcome data (attrition bias)
(**F**) Selective reporting (reporting bias)
(**G**) Other bias

3.3 Delivery during admission

Study or Subgroup	Cholecystectomy Events	Cholecystectomy Total	Medical Tx Events	Medical Tx Total	Weight	Odds Ratio M-H, Random, 95% CI	Odds Ratio M-H, Random, 95% CI	Risk of Bias A B C D E F
Davis 1995	5	19	7	23	57.3%	0.82 [0.21, 3.16]		
Kolbeinsson 2016	1	15	3	45	19.1%	1.00 [0.10, 10.41]		
Lu 2004	1	13	20	65	23.6%	0.19 [0.02, 1.54]		
**Total (95% CI)**		**47**		**133**	**100.0%**	**0.60 [0.22, 1.67]**		
Total events	7		30					

Heterogeneity: Tau² = 0.00; Chi² = 1.62, df = 2 (P = 0.44); I² = 0%
Test for overall effect: Z = 0.98 (P = 0.33)

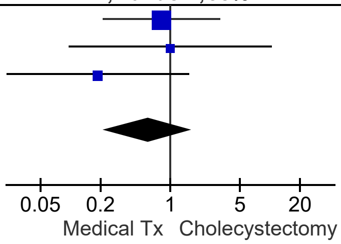

Risk of bias legend
(**A**) Random sequence generation (selection bias)
(**B**) Allocation concealment (selection bias)
(**C**) Blinding of participants and personnel (performance bias)
(**D**) Blinding of outcome assessment (detection bias)
(**E**) Incomplete outcome data (attrition bias)
(**F**) Selective reporting (reporting bias)
(**G**) Other bias

3.4 IUGR

Study or Subgroup	Cholecystectomy Events	Cholecystectomy Total	Medical Tx Events	Medical Tx Total	Weight	Odds Ratio M-H, Random, 95% CI
**3.4.1 Cholecystitis only**						
Rios-Diaz 2020	14	2440	104	3950	39.1%	0.21 [0.12, 0.37]
**Subtotal (95% CI)**		**2440**		**3950**	**39.1%**	**0.21 [0.12, 0.37]**
Total events	14		104			
Heterogeneity: Not applicable						
Test for overall effect: Z = 5.40 (P < 0.00001)						
**3.4.2 Others**						
Davis 1995	0	19	0	23		Not estimable
Hedström 2017	4	34	1	62	29.3%	8.13 [0.87, 75.98]
İlhan 2016	2	15	3	44	31.7%	2.10 [0.32, 13.99]
**Subtotal (95% CI)**		**68**		**129**	**60.9%**	**3.70 [0.87, 15.71]**
Total events	6		4			
Heterogeneity: Tau² = 0.00; Chi² = 0.83, df = 1 (P = 0.36); I² = 0%						
Test for overall effect: Z = 1.78 (P = 0.08)						
**Total (95% CI)**		**2508**		**4079**	**100.0%**	**1.28 [0.12, 13.29]**
Total events	20		108			
Heterogeneity: Tau² = 3.57; Chi² = 13.84, df = 2 (P = 0.0010); I² = 86%						
Test for overall effect: Z = 0.21 (P = 0.84)						
Test for subgroup differences: Chi² = 13.02, df = 1 (P = 0.0003), I² = 92.3%						

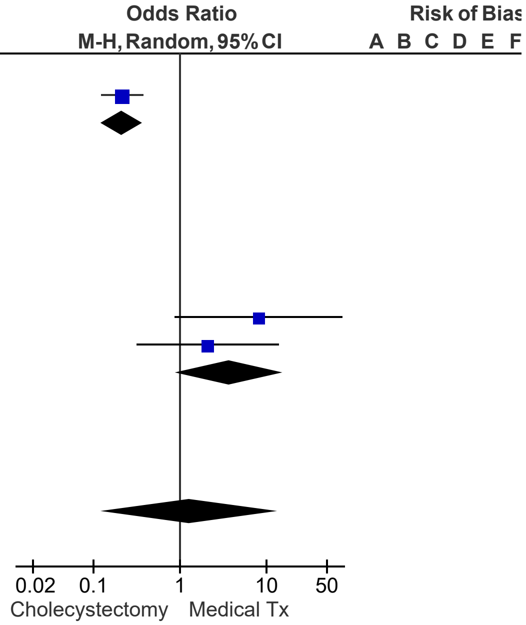

Risk of bias legend
(**A**) Random sequence generation (selection bias)
(**B**) Allocation concealment (selection bias)
(**C**) Blinding of participants and personnel (performance bias)
(**D**) Blinding of outcome assessment (detection bias)
(**E**) Incomplete outcome data (attrition bias)
(**F**) Selective reporting (reporting bias)
(**G**) Other bias

3.5 Neonatal death

Study or Subgroup	Cholecystectomy Events	Cholecystectomy Total	Medical Tx Events	Medical Tx Total	Weight	Odds Ratio M-H, Random, 95% CI
ChiappettaPorras 2009	0	54	0	53		Not estimable
Davis 1995	0	19	0	23		Not estimable
Lu 2004	0	13	2	65	100.0%	0.94 [0.04, 20.73]
**Total (95% CI)**		**86**		**141**	**100.0%**	**0.94 [0.04, 20.73]**
Total events	0		2			

Heterogeneity: Not applicable
Test for overall effect: Z = 0.04 (P = 0.97)

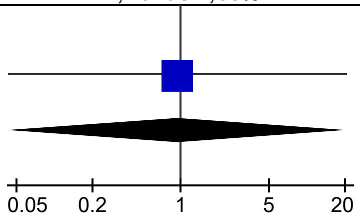

Risk of bias legend
(**A**) Random sequence generation (selection bias)
(**B**) Allocation concealment (selection bias)
(**C**) Blinding of participants and personnel (performance bias)
(**D**) Blinding of outcome assessment (detection bias)
(**E**) Incomplete outcome data (attrition bias)
(**F**) Selective reporting (reporting bias)
(**G**) Other bias

3.6 NICU

Study or Subgroup	Cholecystectomy Events	Cholecystectomy Total	Medical Tx Events	Medical Tx Total	Weight	Odds Ratio M-H, Random, 95% CI
Davis 1995	0	19	1	23	43.9%	0.38 [0.01, 9.99]
Lu 2004	0	13	15	65	56.1%	0.12 [0.01, 2.15]
**Total (95% CI)**		**32**		**88**	**100.0%**	**0.20 [0.02, 1.74]**
Total events	0		16			

Heterogeneity: $Tau^2 = 0.00$; $Chi^2 = 0.29$, df = 1 (P = 0.59); $I^2 = 0\%$
Test for overall effect: Z = 1.46 (P = 0.14)

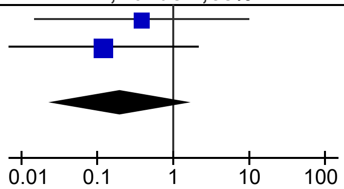

Risk of bias legend
(**A**) Random sequence generation (selection bias)
(**B**) Allocation concealment (selection bias)
(**C**) Blinding of participants and personnel (performance bias)
(**D**) Blinding of outcome assessment (detection bias)
(**E**) Incomplete outcome data (attrition bias)
(**F**) Selective reporting (reporting bias)
(**G**) Other bias

3.7 Pre-eclampsia

Study or Subgroup	Cholecystectomy Events	Cholecystectomy Total	Medical Tx Events	Medical Tx Total	Weight	Odds Ratio M-H, Random, 95% CI
**3.7.1 Cholecystitis only**						
Rios-Diaz 2020	224	2440	603	3950	41.6%	0.56 [0.48, 0.66]
**Subtotal (95% CI)**		**2440**		**3950**	**41.6%**	**0.56 [0.48, 0.66]**
Total events	224		603			
Heterogeneity: Not applicable						
Test for overall effect: Z = 6.97 (P < 0.00001)						
**3.7.2 Others**						
Davis 1995	0	19	0	23		Not estimable
Fong 2019	9	403	184	17490	38.2%	2.15 [1.09, 4.23]
Hong 2020	1	82	3	5040	20.2%	20.73 [2.13, 201.40]
**Subtotal (95% CI)**		**504**		**22553**	**58.4%**	**5.12 [0.58, 45.37]**
Total events	10		187			
Heterogeneity: $Tau^2 = 1.89$; $Chi^2 = 3.58$, df = 1 (P = 0.06); $I^2 = 72\%$						
Test for overall effect: Z = 1.47 (P = 0.14)						
**Total (95% CI)**		**2944**		**26503**	**100.0%**	**1.94 [0.47, 8.04]**
Total events	234		790			
Heterogeneity: $Tau^2 = 1.26$; $Chi^2 = 23.69$, df = 2 (P < 0.00001); $I^2 = 92\%$						
Test for overall effect: Z = 0.92 (P = 0.36)						
Test for subgroup differences: $Chi^2 = 3.92$, df = 1 (P = 0.05), $I^2 = 74.5\%$						

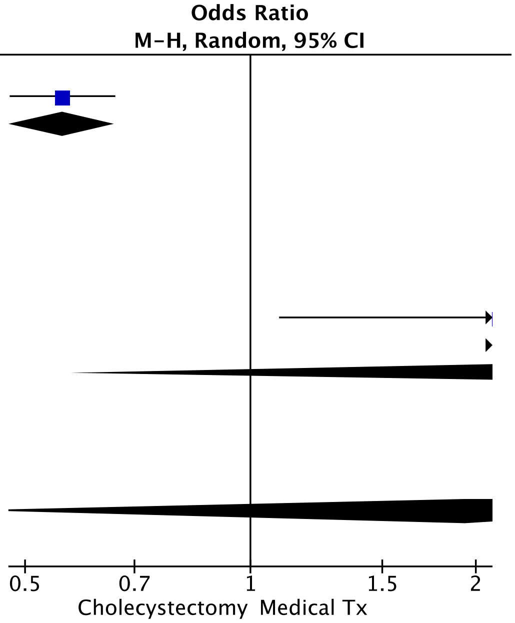

(**A**) Random sequence generation (selection bias)
(**B**) Allocation concealment (selection bias)
(**C**) Blinding of participants and personnel (performance bias)
(**D**) Blinding of outcome assessment (detection bias)
(**E**) Incomplete outcome data (attrition bias)
(**F**) Selective reporting (reporting bias)
(**G**) Other bias

3.8 Preg loss - all

Study or Subgroup	Cholecystectomy Events	Cholecystectomy Total	Medical Tx Events	Medical Tx Total	Weight	Odds Ratio M-H, Random, 95% CI	Risk of bias A B C D
**3.8.1 Cholecystitis only**							
Rios-Diaz 2020	14	2440	37	3950	90.3%	0.61 [0.33, 1.13]	
**Subtotal (95% CI)**		**2440**		**3950**	**90.3%**	**0.61 [0.33, 1.13]**	
Total events	14		37				
Heterogeneity: Not applicable							
Test for overall effect: Z = 1.57 (P = 0.12)							
**3.8.2 Others**							
Barut 2019	0	6	0	14		Not estimable	
ChiappettaPorras 2009	0	54	0	53		Not estimable	
Davis 1995	0	19	0	23		Not estimable	
İlhan 2016	0	15	1	44	3.2%	0.94 [0.04, 24.19]	
Kolbeinsson 2016	1	15	0	45	3.2%	9.41 [0.36, 243.94]	
Lu 2004	0	13	1	65	3.2%	1.59 [0.06, 41.23]	
**Subtotal (95% CI)**		**122**		**244**	**9.7%**	**2.41 [0.37, 15.77]**	
Total events	1		2				
Heterogeneity: Tau² = 0.00; Chi² = 1.06, df = 2 (P = 0.59); I² = 0%							
Test for overall effect: Z = 0.92 (P = 0.36)							
**Total (95% CI)**		**2562**		**4194**	**100.0%**	**0.70 [0.39, 1.25]**	
Total events	15		39				
Heterogeneity: Tau² = 0.00; Chi² = 2.92, df = 3 (P = 0.40); I² = 0%							
Test for overall effect: Z = 1.20 (P = 0.23)							
Test for subgroup differences: Chi² = 1.85, df = 1 (P = 0.17), I² = 46.1%							

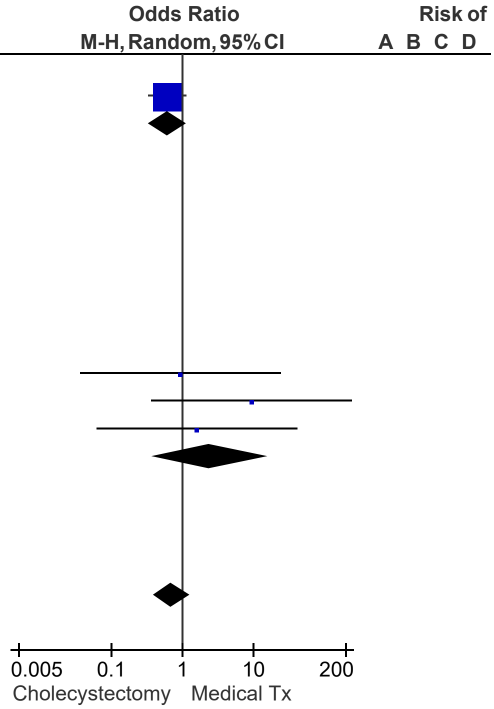

Risk of bias legend
(**A**) Random sequence generation (selection bias)
(**B**) Allocation concealment (selection bias)
(**C**) Blinding of participants and personnel (performance bias)
(**D**) Blinding of outcome assessment (detection bias)
(**E**) Incomplete outcome data (attrition bias)
(**F**) Selective reporting (reporting bias)
(**G**) Other bias

3.9 Preg loss - <20

Study or Subgroup	Cholecystectomy Events	Cholecystectomy Total	Medical Tx Events	Medical Tx Total	Weight	Odds Ratio M-H, Random, 95% CI	Odds Ratio M-H, Random, 95% CI	Risk of bias A B C D
ChiappettaPorras 2009	0	54	0	53		Not estimable		
Hedström 2017	2	34	1	62	64.0%	3.81 [0.33, 43.67]		
İlhan 2016	0	15	1	44	36.0%	0.94 [0.04, 24.19]		
Lu 2004	0	13	0	65		Not estimable		
**Total (95% CI)**		**116**		**224**	**100.0%**	**2.30 [0.33, 16.18]**		
Total events	2		2					

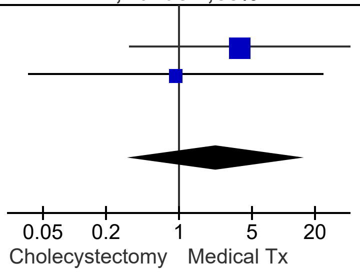

Heterogeneity: Tau² = 0.00; Chi² = 0.46, df = 1 (P = 0.50); I² = 0%
Test for overall effect: Z = 0.84 (P = 0.40)

Risk of bias legend
(**A**) Random sequence generation (selection bias)
(**B**) Allocation concealment (selection bias)
(**C**) Blinding of participants and personnel (performance bias)
(**D**) Blinding of outcome assessment (detection bias)
(**E**) Incomplete outcome data (attrition bias)
(**F**) Selective reporting (reporting bias)
(**G**) Other bias

3.10 Preg loss - >20

Study or Subgroup	Cholecystectomy Events	Cholecystectomy Total	Medical Tx Events	Medical Tx Total	Weight	Odds Ratio M-H, Random, 95% CI	Odds Ratio M-H, Random, 95% CI	Risk of bias A B C D
ChiappettaPorras 2009	0	54	0	53		Not estimable		
Davis 1995	0	19	0	23		Not estimable		
Kolbeinsson 2016	1	15	0	45	50.0%	9.41 [0.36, 243.94]		
Lu 2004	0	13	1	65	50.0%	1.59 [0.06, 41.23]		
**Total (95% CI)**		**101**		**186**	**100.0%**	**3.87 [0.39, 38.66]**		
Total events	1		1					

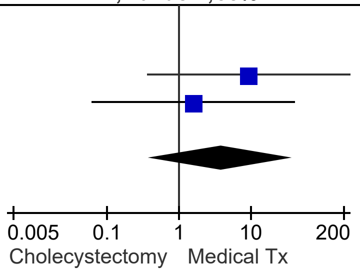

Heterogeneity: Tau² = 0.00; Chi² = 0.57, df = 1 (P = 0.45); I² = 0%
Test for overall effect: Z = 1.15 (P = 0.25)

Risk of bias legend
(**A**) Random sequence generation (selection bias)
(**B**) Allocation concealment (selection bias)
(**C**) Blinding of participants and personnel (performance bias)
(**D**) Blinding of outcome assessment (detection bias)
(**E**) Incomplete outcome data (attrition bias)
(**F**) Selective reporting (reporting bias)
(**G**) Other bias

3.11 Preterm

Study or Subgroup	Cholecystectomy Events	Cholecystectomy Total	Medical Tx Events	Medical Tx Total	Weight	Odds Ratio M-H, Random, 95% CI
**3.11.1 Cholecystitis only**						
Rios-Diaz 2020	91	2440	397	3950	13.8%	0.35 [0.27, 0.44]
**Subtotal (95% CI)**		**2440**		**3950**	**13.8%**	**0.35 [0.27, 0.44]**
Total events	91		397			
Heterogeneity: Not applicable						
Test for overall effect: Z = 8.88 (P < 0.00001)						
**3.11.2 Others**						
Barut 2019	0	6	4	14	5.2%	0.18 [0.01, 3.91]
Bowie 2020	148	746	866	6816	13.8%	1.70 [1.40, 2.06]
Davis 1995	5	19	2	23	8.9%	3.75 [0.64, 22.10]
Fong 2019	87	403	1684	17490	13.7%	2.58 [2.03, 3.29]
Hong 2020	15	82	22	5040	12.8%	51.07 [25.38, 102.75]
Ibiebele 2017	18	239	137	1643	13.3%	0.90 [0.54, 1.49]
İlhan 2016	2	15	2	44	8.0%	3.23 [0.41, 25.26]
Kolbeinsson 2016	0	15	1	45	4.9%	0.96 [0.04, 24.74]
Lu 2004	0	13	11	65	5.6%	0.18 [0.01, 3.17]
**Subtotal (95% CI)**		**1538**		**31180**	**86.2%**	**2.50 [1.15, 5.41]**
Total events	275		2729			
Heterogeneity: $Tau^2 = 0.85; Chi^2 = 106.64, df = 8 (P < 0.00001); I^2 = 92\%$						
Test for overall effect: Z = 2.33 (P = 0.02)						
**Total (95% CI)**		**3978**		**35130**	**100.0%**	**1.77 [0.73, 4.30]**
Total events	366		3126			
Heterogeneity: $Tau^2 = 1.48; Chi^2 = 288.27, df = 9 (P < 0.00001); I^2 = 97\%$						
Test for overall effect: Z = 1.25 (P = 0.21)						
Test for subgroup differences: $Chi^2 = 23.05, df = 1 (P < 0.00001), I^2 = 95.7\%$						

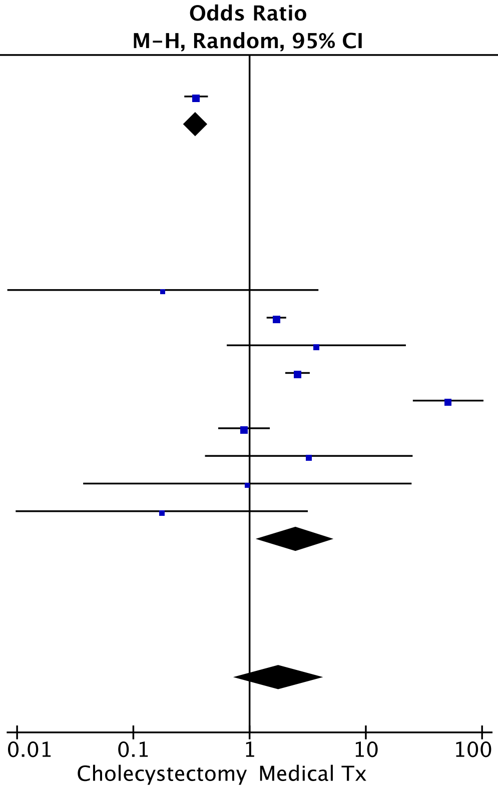

(**A**) Random sequence generation (selection bias)
(**B**) Allocation concealment (selection bias)
(**C**) Blinding of participants and personnel (performance bias)
(**D**) Blinding of outcome assessment (detection bias)
(**E**) Incomplete outcome data (attrition bias)
(**F**) Selective reporting (reporting bias)
(**G**) Other bias

3.13 Readmit

Study or Subgroup	Cholecystectomy Events	Cholecystectomy Total	Medical Tx Events	Medical Tx Total	Weight	Odds Ratio M-H, Random, 95% CI
**3.13.1 Cholecystitis only**						
Rios-Diaz 2020	262	2440	739	3950	21.3%	0.52 [0.45, 0.61]
**Subtotal (95% CI)**		**2440**		**3950**	**21.3%**	**0.52 [0.45, 0.61]**
Total events	262		739			
Heterogeneity: Not applicable						
Test for overall effect: Z = 8.42 (P < 0.00001)						
**3.13.2 Others**						
Barut 2019	0	6	11	14	6.3%	0.02 [0.00, 0.53]
Davis 1995	0	19	8	23	6.8%	0.05 [0.00, 0.87]
Fong 2019	38	403	676	17490	20.8%	2.59 [1.84, 3.65]
Hedström 2017	1	27	24	70	10.4%	0.07 [0.01, 0.58]
Hong 2020	2	82	156	5040	14.3%	0.78 [0.19, 3.21]
Ibiebele 2017	18	239	370	1643	20.2%	0.28 [0.17, 0.46]
**Subtotal (95% CI)**		**776**		**24280**	**78.7%**	**0.27 [0.06, 1.25]**
Total events	59		1245			
Heterogeneity: $Tau^2 = 2.89$; $Chi^2 = 82.32$, df = 5 ($P < 0.00001$); $I^2 = 94\%$						
Test for overall effect: Z = 1.67 (P = 0.09)						
**Total (95% CI)**		**3216**		**28230**	**100.0%**	**0.39 [0.15, 0.98]**
Total events	321		1984			
Heterogeneity: $Tau^2 = 1.04$; $Chi^2 = 95.66$, df = 6 ($P < 0.00001$); $I^2 = 94\%$						
Test for overall effect: Z = 2.00 (P = 0.05)						
Test for subgroup differences: $Chi^2 = 0.72$, df = 1 ($P = 0.40$), $I^2 = 0\%$						

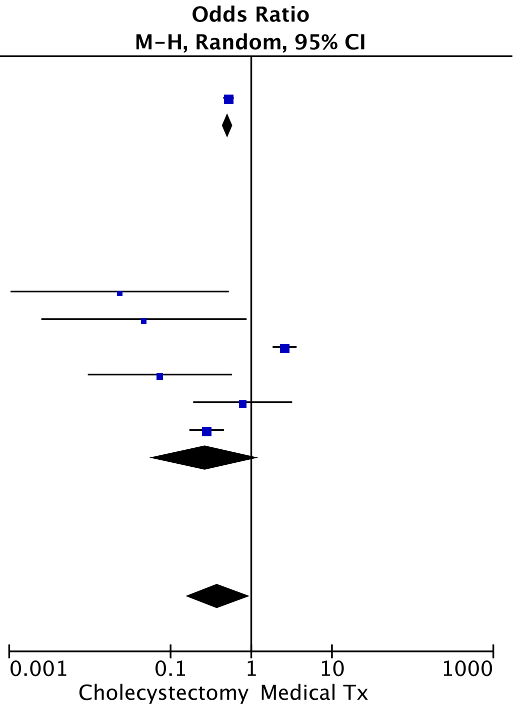

(**A**) Random sequence generation (selection bias)
(**B**) Allocation concealment (selection bias)
(**C**) Blinding of participants and personnel (performance bias)
(**D**) Blinding of outcome assessment (detection bias)
(**E**) Incomplete outcome data (attrition bias)
(**F**) Selective reporting (reporting bias)
(**G**) Other bias

3.14 Sepsis

Study or Subgroup	Cholecystectomy Events	Cholecystectomy Total	Medical Tx Events	Medical Tx Total	Weight	Odds Ratio M-H, Random, 95% CI	Risk of Bias A	B	C	D	E	F
**3.14.1 Cholecystitis only**												
Rios-Diaz 2020	77	2440	69	3950	80.4%	1.83 [1.32, 2.55]						
**Subtotal (95% CI)**		**2440**		**3950**	**80.4%**	**1.83 [1.32, 2.55]**						
Total events	77		69									
Heterogeneity: Not applicable												
Test for overall effect: Z = 3.61 (P = 0.0003)												
**3.14.2 Others**												
Davis 1995	0	19	0	23		Not estimable						
Juo 2018	8	374	17	871	19.6%	1.10 [0.47, 2.57]						
**Subtotal (95% CI)**		**393**		**894**	**19.6%**	**1.10 [0.47, 2.57]**						
Total events	8		17									
Heterogeneity: Not applicable												
Test for overall effect: Z = 0.22 (P = 0.83)												
**Total (95% CI)**		**2833**		**4844**	**100.0%**	**1.66 [1.11, 2.47]**						
Total events	85		86									

Heterogeneity: $Tau^2 = 0.02$; $Chi^2 = 1.22$, df = 1 (P = 0.27); $I^2 = 18\%$
Test for overall effect: Z = 2.48 (P = 0.01)
Test for subgroup differences: $Chi^2 = 1.22$, df = 1 (P = 0.27), $I^2 = 17.8\%$

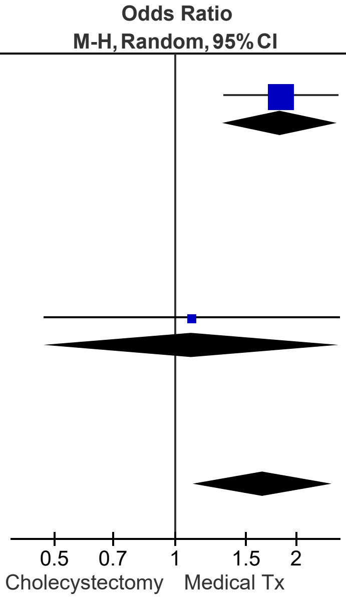

Risk of bias legend
(**A**) Random sequence generation (selection bias)
(**B**) Allocation concealment (selection bias)
(**C**) Blinding of participants and personnel (performance bias)
(**D**) Blinding of outcome assessment (detection bias)
(**E**) Incomplete outcome data (attrition bias)
(**F**) Selective reporting (reporting bias)
(**G**) Other bias
