# Supplementary material for: SAGES guidelines for the use of laparoscopy during pregnancy
Source: Surg Endosc. 2024 May 3;38(6):2947–63. doi: 10.1007/s00464-024-10810-1 (PMC11133165; doi:10.1007/s00464-024-10810-1)
Supplement: Supplementary file 5 — Supplementary file5 (ZIP 64 kb) [file 464_2024_10810_MOESM5_ESM.zip › 464_2024_10810_MOESM5_ESM/Appendix E KQ1 Evidence Table.pdf]

Author(s):
Question: KQ1 Appendectomy compared to medical management for appendicitis during pregnancy (any trimester)
Setting:
Bibliography: . [Intervention] for [health problem]. Cochrane Database of Systematic Reviews [Year], Issue [Issue].

Certainty assessment							№ of patients		Effect		Certainty	Importance
№ of studies	Study design	Risk of bias	Inconsistency	Indirectness	Imprecision	Other considerations	KQ1 Appendectomy	medical management	Relative (95% CI)	Absolute (95% CI)		
**C-Section**												
1	observational studies	serious[a]	not serious	not serious	very serious[b]	none	4/20 (20.0%)	9/34 (26.5%)	**OR 0.69** (0.18 to 2.64)	**66 fewer per 1,000** (from 204 fewer to 223 more)	⊕◯◯◯ Very low	
**Delivery**												
1	observational studies	serious[c]	not serious	not serious	very serious[b]	none	0/3 (0.0%)	0/17 (0.0%)	not estimable		⊕◯◯◯ Very low	
**Pregnancy loss (total, any gestation)**												
3	observational studies	serious[c]	not serious	not serious	very serious[b]	none	4/79 (5.1%)	6/164 (3.7%)	**OR 1.32** (0.36 to 4.85)	**11 more per 1,000** (from 23 fewer to 119 more)	⊕◯◯◯ Very low	
**Preterm Birth**												
2	observational studies	serious[c]	not serious	not serious	very serious[b]	none	2/23 (8.7%)	3/51 (5.9%)	**OR 1.15** (0.18 to 7.53)	**8 more per 1,000** (from 48 fewer to 261 more)	⊕◯◯◯ Very low	
**Readmission**												
1	observational studies	serious[a]	not serious	not serious	very serious[b]	none	0/20 (0.0%)	3/34 (8.8%)	**OR 0.22** (0.01 to 4.48)	**67 fewer per 1,000** (from 87 fewer to 214 more)	⊕◯◯◯ Very low	
**Sepsis**												
1	observational studies	serious[c]	not serious	not serious	very serious[b]	none	10/6701 (0.1%)	4/413 (1.0%)	**OR 0.15** (0.05 to 0.49)	**8 fewer per 1,000** (from 9 fewer to 5 fewer)	⊕◯◯◯ Very low	

CI: confidence interval; **OR:** odds ratio

Explanations

a. This study had an unclear risk of bias on the Newcastle-Ottawa Scale due to uncertainty around the selection of patients and its retrospective nature.
b. This outcome had a very small sample size and an even smaller event size which increases its fragility.
c. This study had a high risk of bias on the Newcastle-Ottawa Scale due to concerns around the comparability of groups and reporting of outcomes.
