# Supplementary material for: SAGES guidelines for the use of laparoscopy during pregnancy
Source: Surg Endosc. 2024 May 3;38(6):2947–63. doi: 10.1007/s00464-024-10810-1 (PMC11133165; doi:10.1007/s00464-024-10810-1)
Supplement: Supplementary file 5 — Supplementary file5 (ZIP 64 kb) [file 464_2024_10810_MOESM5_ESM.zip › 464_2024_10810_MOESM5_ESM/Appendix E KQ2 Evidence Table.docx]

**Author(s):**

**Question:** KQ2 Laparoscopic appendectomy compared to open appendectomy for appendicitis during pregnancy (any trimester)

**Setting:**

**Bibliography:** . [Intervention] for [health problem]. Cochrane Database of Systematic Reviews [Year], Issue [Issue].

| **Certainty assessment** | | | | | | | **№ of patients** | | **Effect** | | **Certainty** | **Importance** |
| --- | --- | --- | --- | --- | --- | --- | --- | --- | --- | --- | --- | --- |
| **№ of studies** | **Study design** | **Risk of bias** | **Inconsistency** | **Indirectness** | **Imprecision** | **Other considerations** | **KQ2 Laparoscopic appendectomy** | **open appendectomy** | **Relative (95% CI)** | **Absolute (95% CI)** |  |  |
| **C-Section** | | | | | | | | | | | | |
| 11 | observational studies | serious^a^ | not serious | not serious | not serious | none | 349/869 (40.2%) | 538/1397 (38.5%) | **OR 1.10** (0.91 to 1.33) | **23 more per 1,000** (from 22 fewer to 69 more) | ⨁◯◯◯ Very low |  |
| **Delivery** | | | | | | | | | | | | |
| 2 | observational studies | serious^b^ | not serious | not serious | not serious | none | 0/23 (0.0%) | 1/29 (3.4%) | **OR 0.94** (0.03 to 26.65) | **2 fewer per 1,000** (from 33 fewer to 453 more) | ⨁◯◯◯ Very low |  |
| **Neonatal death** | | | | | | | | | | | | |
| 6 | observational studies | serious^a,b^ | not serious | not serious | not serious | none | 0/133 (0.0%) | 0/184 (0.0%) | not pooled | see comment | ⨁◯◯◯ Very low |  |
| **NICU** | | | | | | | | | | | | |
| 1 | observational studies | serious^c^ | not serious | not serious | not serious | none | 1/18 (5.6%) | 0/13 (0.0%) | **OR 2.31** (0.09 to 61.41) | **0 fewer per 1,000** (from 0 fewer to 0 fewer) | ⨁◯◯◯ Very low |  |
| **Preg loss - all** | | | | | | | | | | | | |
| 27 | observational studies | serious^a^ | not serious | not serious | not serious | none | 65/1714 (3.8%) | 137/4474 (3.1%) | **OR 1.93** (1.39 to 2.70) | **27 more per 1,000** (from 11 more to 48 more) | ⨁◯◯◯ Very low |  |
| **Preg loss - <20** | | | | | | | | | | | | |
| 11 | observational studies | serious^a^ | not serious | not serious | not serious | none | 13/252 (5.2%) | 2/273 (0.7%) | **OR 3.20** (0.91 to 11.22) | **16 more per 1,000** (from 1 fewer to 69 more) | ⨁◯◯◯ Very low |  |
| **Preg loss - >20** | | | | | | | | | | | | |
| 8 | observational studies | serious^a^ | not serious | not serious | not serious | none | 1/205 (0.5%) | 1/224 (0.4%) | **OR 1.47** (0.15 to 14.52) | **2 more per 1,000** (from 4 fewer to 57 more) | ⨁◯◯◯ Very low |  |
| **Preterm** | | | | | | | | | | | | |
| 21 | observational studies | serious^a^ | not serious | not serious | not serious | none | 110/1603 (6.9%) | 394/4380 (9.0%) | **OR 0.86** (0.55 to 1.35) | **12 fewer per 1,000** (from 38 fewer to 28 more) | ⨁◯◯◯ Very low |  |
| **Readmit** | | | | | | | | | | | | |
| 3 | observational studies | serious^d^ | not serious | not serious | not serious | none | 18/583 (3.1%) | 20/511 (3.9%) | **OR 0.79** (0.41 to 1.51) | **8 fewer per 1,000** (from 23 fewer to 19 more) | ⨁◯◯◯ Very low |  |
| **Sepsis** | | | | | | | | | | | | |
| 2 | observational studies | serious^a^ | not serious | not serious | not serious | none | 7/1441 (0.5%) | 7/900 (0.8%) | **OR 0.58** (0.20 to 1.69) | **3 fewer per 1,000** (from 6 fewer to 5 more) | ⨁◯◯◯ Very low |  |

**CI:** confidence interval; **OR:** odds ratio

#### Explanations

a. Included studies with a high risk of bias on the Newcastle-Ottawa scale due to comparability of the groups.

b. Included studies with an unclear risk of bias on the Newcastle-Ottawa scale due to potential biases in the selection of patients and comparability of groups.

c. Included studies with an unclear risk of bias on the Newcastle-Ottawa scale due to potential biases in the selection of patients.

d. Included studies with an unclear risk of bias on the Newcastle-Ottawa scale due to potential biases in the comparability of groups.
