# Supplementary material for: SAGES guidelines for the use of laparoscopy during pregnancy
Source: Surg Endosc. 2024 May 3;38(6):2947–63. doi: 10.1007/s00464-024-10810-1 (PMC11133165; doi:10.1007/s00464-024-10810-1)
Supplement: Supplementary file 5 — Supplementary file5 (ZIP 64 kb) [file 464_2024_10810_MOESM5_ESM.zip › 464_2024_10810_MOESM5_ESM/Appendix E KQ3 Evidence Table.docx]

**Author(s):**

**Question:** Cholecystectomy compared to Medical Treatment for biliary disease in pregnancy

**Setting:**

**Bibliography:** . [Intervention] for [health problem]. Cochrane Database of Systematic Reviews [Year], Issue [Issue].

| **Certainty assessment** | | | | | | | **№ of patients** | | **Effect** | | **Certainty** | **Importance** |
| --- | --- | --- | --- | --- | --- | --- | --- | --- | --- | --- | --- | --- |
| **№ of studies** | **Study design** | **Risk of bias** | **Inconsistency** | **Indirectness** | **Imprecision** | **Other considerations** | **Cholecystectomy** | **Medical Treatment** | **Relative (95% CI)** | **Absolute (95% CI)** |  |  |
| **Bile leak** | | | | | | | | | | | | |
| 6 | observational studies | serious^a^ | not serious | not serious | serious^b^ | none | 4/599 (0.7%) | 294/22702 (1.3%) | **OR 1.06** (0.17 to 6.53) | **1 more per 1,000** (from 11 fewer to 66 more) | ⨁◯◯◯ Very low |  |
| **C-Section** | | | | | | | | | | | | |
| 9 | observational studies | serious^a^ | not serious | not serious | serious^b^ | none | 443/3249 (13.6%) | 10699/28367 (37.7%) | **OR 0.87** (0.36 to 2.10) | **32 fewer per 1,000** (from 198 fewer to 183 more) | ⨁◯◯◯ Very low |  |
| **C-Section - Cholecystitis only** | | | | | | | | | | | | |
| 1 | observational studies | not serious | not serious | not serious | not serious | none | 147/2440 (6.0%) | 984/3950 (24.9%) | **OR 0.19** (0.16 to 0.23) | **190 fewer per 1,000** (from 199 fewer to 178 fewer) | ⨁⨁◯◯ Low |  |
| **Delivery during admission** | | | | | | | | | | | | |
| 3 | observational studies | serious^a^ | not serious | not serious | serious^b^ | none | 7/47 (14.9%) | 30/133 (22.6%) | **OR 0.60** (0.22 to 1.67) | **77 fewer per 1,000** (from 165 fewer to 102 more) | ⨁◯◯◯ Very low |  |
| **IUGR** | | | | | | | | | | | | |
| 4 | observational studies | serious^a^ | serious^c^ | not serious | serious^b^ | none | 20/2508 (0.8%) | 108/4079 (2.6%) | **OR 1.28** (0.12 to 13.29) | **7 more per 1,000** (from 23 fewer to 239 more) | ⨁◯◯◯ Very low |  |
| **IUGR - Cholecystitis only** | | | | | | | | | | | | |
| 1 | observational studies | not serious | not serious | not serious | not serious | none | 14/2440 (0.6%) | 104/3950 (2.6%) | **OR 0.21** (0.12 to 0.37) | **21 fewer per 1,000** (from 23 fewer to 16 fewer) | ⨁⨁◯◯ Low |  |
| **Neonatal death** | | | | | | | | | | | | |
| 3 | observational studies | serious^a^ | not serious | not serious | serious^b^ | none | 0/86 (0.0%) | 2/141 (1.4%) | **OR 0.94** (0.04 to 20.73) | **1 fewer per 1,000** (from 14 fewer to 216 more) | ⨁◯◯◯ Very low |  |
| **NICU** | | | | | | | | | | | | |
| 2 | observational studies | serious^a^ | not serious | not serious | serious^b^ | none | 0/32 (0.0%) | 16/88 (18.2%) | **OR 0.20** (0.02 to 1.74) | **139 fewer per 1,000** (from 177 fewer to 97 more) | ⨁◯◯◯ Very low |  |
| **Pre-eclampsia** | | | | | | | | | | | | |
| 4 | observational studies | serious^a^ | serious^c^ | not serious | not serious | none | 234/2944 (7.9%) | 790/26503 (3.0%) | **OR 1.94** (0.47 to 8.04) | **26 more per 1,000** (from 16 fewer to 168 more) | ⨁◯◯◯ Very low |  |
| **Pre-eclampsia - Cholecystitis only** | | | | | | | | | | | | |
| 1 | observational studies | not serious | not serious | not serious | not serious | none | 224/2440 (9.2%) | 603/3950 (15.3%) | **OR 0.56** (0.48 to 0.66) | **61 fewer per 1,000** (from 73 fewer to 46 fewer) | ⨁⨁◯◯ Low |  |
| **Preg loss - all** | | | | | | | | | | | | |
| 7 | observational studies | serious^a^ | not serious | not serious | serious^b^ | none | 15/2562 (0.6%) | 39/4194 (0.9%) | **OR 0.70** (0.39 to 1.25) | **3 fewer per 1,000** (from 6 fewer to 2 more) | ⨁◯◯◯ Very low |  |
| **Preg loss - all - Cholecystitis only** | | | | | | | | | | | | |
| 1 | observational studies | not serious | not serious | not serious | serious^b^ | none | 14/2440 (0.6%) | 37/3950 (0.9%) | **OR 0.61** (0.33 to 1.13) | **4 fewer per 1,000** (from 6 fewer to 1 more) | ⨁◯◯◯ Very low |  |
| **Preg loss - <20** | | | | | | | | | | | | |
| 4 | observational studies | serious^a^ | not serious | not serious | serious^b^ | none | 2/116 (1.7%) | 2/224 (0.9%) | **OR 2.30** (0.33 to 16.18) | **11 more per 1,000** (from 6 fewer to 118 more) | ⨁◯◯◯ Very low |  |
| **Preg loss - >20** | | | | | | | | | | | | |
| 4 | observational studies | serious^a^ | not serious | not serious | serious^b^ | none | 1/101 (1.0%) | 1/186 (0.5%) | **OR 3.87** (0.39 to 38.66) | **15 more per 1,000** (from 3 fewer to 167 more) | ⨁◯◯◯ Very low |  |
| **Preterm** | | | | | | | | | | | | |
| 10 | observational studies | serious^a^ | serious^c^ | not serious | serious^b^ | none | 366/3978 (9.2%) | 3126/35130 (8.9%) | **OR 1.77** (0.73 to 4.30) | **58 more per 1,000** (from 22 fewer to 207 more) | ⨁◯◯◯ Very low |  |
| **Preterm - Cholecystitis only** | | | | | | | | | | | | |
| 1 | observational studies | not serious | not serious | not serious | not serious | none | 91/2440 (3.7%) | 397/3950 (10.1%) | **OR 0.35** (0.27 to 0.44) | **63 fewer per 1,000** (from 71 fewer to 54 fewer) | ⨁⨁◯◯ Low |  |
| **Readmit** | | | | | | | | | | | | |
| 7 | observational studies | serious^a^ | not serious | not serious | not serious | none | 321/3216 (10.0%) | 1984/28230 (7.0%) | **OR 0.39** (0.15 to 0.98) | **42 fewer per 1,000** (from 59 fewer to 1 fewer) | ⨁◯◯◯ Very low |  |
| **Readmit - Cholecystitis only** | | | | | | | | | | | | |
| 1 | observational studies | not serious | not serious | not serious | not serious | none | 262/2440 (10.7%) | 739/3950 (18.7%) | **OR 0.52** (0.45 to 0.61) | **80 fewer per 1,000** (from 93 fewer to 64 fewer) | ⨁⨁◯◯ Low |  |
| **Sepsis** | | | | | | | | | | | | |
| 3 | observational studies | serious^a^ | not serious | not serious | serious^b^ | none | 85/2833 (3.0%) | 86/4844 (1.8%) | **OR 1.66** (1.11 to 2.47) | **11 more per 1,000** (from 2 more to 25 more) | ⨁◯◯◯ Very low |  |
| **Sepsis - Cholecystitis only** | | | | | | | | | | | | |
| 1 | observational studies | not serious | not serious | not serious | not serious | none | 77/2440 (3.2%) | 69/3950 (1.7%) | **OR 1.83** (1.32 to 2.55) | **14 more per 1,000** (from 5 more to 26 more) | ⨁⨁◯◯ Low |  |

**CI:** confidence interval; **OR:** odds ratio

#### Explanations

a. Some of the included studies which contributed significantly to the overall effect size were deemed to be at a high risk of bias on the Newcastle-Ottawa scale due to comparability.

b. There was a wide range of effects that crosses several clinically relevant thresholds.

c. There was serious inconsistency between some of the included studies, with non-overlapping confidence intervals.
