# Supplementary material for: SAGES guidelines for the use of laparoscopy during pregnancy
Source: Surg Endosc. 2024 May 3;38(6):2947–63. doi: 10.1007/s00464-024-10810-1 (PMC11133165; doi:10.1007/s00464-024-10810-1)
Supplement: Supplementary file 6 — Supplementary file6 (ZIP 91 kb) [file 464_2024_10810_MOESM6_ESM.zip › 464_2024_10810_MOESM6_ESM/Appendix F KQ1 Evidence to decision table.docx]

| Key question 1 | |
| --- | --- |
| **Should appendectomy vs. medical management be used for appendicitis during pregnancy (any trimester)?** | |
| **Population:** | appendicitis during pregnancy (any trimester) |
| **Intervention:** | KQ1 Appendectomy |
| **Comparison:** | medical management |
| **Main outcomes:** | C-Section; Delivery; Pregnancy loss (total, any gestation); Preterm Birth; Readmission; Sepsis; |
| **Setting:** |  |
| **Perspective:** |  |
| **Background:** |  |
| **Conflict of interests:** |  |

# Assessment

| Problem Is the problem a priority? | | |
| --- | --- | --- |
| Judgement | Research evidence | Additional considerations |
| ○ No ○ Probably no ○ Probably yes •**Yes** ○ Varies ○ Don't know |  |  |
| Desirable Effects How substantial are the desirable anticipated effects? | | |
| Judgement | Research evidence | Additional considerations |
| ○ Trivial • **Small** ○ Moderate ○ Large ○ Varies ○ Don't know | \| **Outcomes** \| **№ of participants (studies) Follow-up** \| **Certainty of the evidence (GRADE)** \| **Relative effect (95% CI)** \| **Anticipated absolute effects^*^ (95% CI)** \| \| \| --- \| --- \| --- \| --- \| --- \| --- \| \| **Risk with medical management** \| **Risk difference with KQ1 Appendectomy** \| \| C-Section \| 54 (1 observational study) \| ⨁◯◯◯ Very low^a,b^ \| **OR 0.69** (0.18 to 2.64) \| Study population \| \| \| 265 per 1,000 \| **66 fewer per 1,000** (204 fewer to 223 more) \| \| Readmission \| 54 (1 observational study) \| ⨁◯◯◯ Very low^a,b^ \| **OR 0.22** (0.01 to 4.48) \| Study population \| \| \| 88 per 1,000 \| **67 fewer per 1,000** (87 fewer to 214 more) \| \| Sepsis \| 7114 (1 observational study) \| ⨁◯◯◯ Very low^b,c^ \| **OR 0.15** (0.05 to 0.49) \| Study population \| \| \| 10 per 1,000 \| **8 fewer per 1,000** (9 fewer to 5 fewer) \|  1. This study had an unclear risk of bias on the Newcastle-Ottawa Scale due to uncertainty around the selection of patients and its retrospective nature. 2. This outcome had a very small sample size and an even smaller event size which increases its fragility. 3. This study had a high risk of bias on the Newcastle-Ottawa Scale due to concerns around the comparability of groups and reporting of outcomes. | Other limitations – international papers with variable baseline rates of c-section  Small 6/7, moderate 1/7 |
| Undesirable Effects How substantial are the undesirable anticipated effects? | | |
| Judgement | Research evidence | Additional considerations |
| ○ Large ○ Moderate ○ Small • **Trivial** ○ Varies ○ Don't know | \| **Outcomes** \| **№ of participants (studies) Follow-up** \| **Certainty of the evidence (GRADE)** \| **Relative effect (95% CI)** \| **Anticipated absolute effects^*^ (95% CI)** \| \| \| --- \| --- \| --- \| --- \| --- \| --- \| \| **Risk with medical management** \| **Risk difference with KQ1 Appendectomy** \| \| Pregnancy loss (total, any gestation) \| 243 (3 observational studies) \| ⨁◯◯◯ Very low^a,b^ \| **OR 1.32** (0.36 to 4.85) \| Study population \| \| \| 37 per 1,000 \| **11 more per 1,000** (23 fewer to 119 more) \| \| Preterm Birth \| 74 (2 observational studies) \| ⨁◯◯◯ Very low^a,b^ \| **OR 1.15** (0.18 to 7.53) \| Study population \| \| \| 59 per 1,000 \| **8 more per 1,000** (48 fewer to 261 more) \|  1. This study had a high risk of bias on the Newcastle-Ottawa Scale due to concerns around the comparability of groups and reporting of outcomes. 2. This outcome had a very small sample size and an even smaller event size which increases its fragility. | Also note the inherent selection bias to these papers (Nakashima surgical mgmt. group with much higher rate of complicated appendicits, Joo surgical management group already failed medical management)  Trivial 7/7 |
| Certainty of evidence What is the overall certainty of the evidence of effects? | | |
| Judgement | Research evidence | Additional considerations |
| •**Very low** ○ Low ○ Moderate ○ High ○ No included studies |  |  |
| Values Is there important uncertainty about or variability in how much people value the main outcomes? | | |
| Judgement | Research evidence | Additional considerations |
| ○ Important uncertainty or variability ○ Possibly important uncertainty or variability • **Probably no important uncertainty or variability** ○ No important uncertainty or variability |  |  |
| Balance of effects Does the balance between desirable and undesirable effects favor the intervention or the comparison? | | |
| Judgement | Research evidence | Additional considerations |
| ○ Favors the comparison ○ Probably favors the comparison ○ Does not favor either the intervention or the comparison • **Probably favors the intervention** ○ Favors the intervention ○ Varies ○ Don't know |  | Probably favors intervention 6/7  Does not favor either the intervention or the comparison 1/7  Low quality data with some biases against the surgically managed group but still fairly comparable outcomes |
| Acceptability Is the intervention acceptable to key stakeholders? | | |
| Judgement | Research evidence | Additional considerations |
| ○ No ○ Probably no • **Probably yes** ○ Yes ○ Varies ○ Don't know |  | Probably yes 6/6 |
| Feasibility Is the intervention feasible to implement? | | |
| Judgement | Research evidence | Additional considerations |
| ○ No ○ Probably no •**Probably yes** ○ Yes ○ Varies ○ Don't know |  | Depending on availability of Obstetrics support probably yes 6/7  Yes 1/7 |

# Summary of judgements

|  | **Judgement** | | | | | | |
| --- | --- | --- | --- | --- | --- | --- | --- |
| **Problem** | No | Probably no | Probably yes | **Yes** |  | Varies | Don't know |
| **Desirable Effects** | Trivial | **Small** | Moderate | Large |  | Varies | Don't know |
| **Undesirable Effects** | Large | Moderate | Small | **Trivial** |  | Varies | Don't know |
| **Certainty of evidence** | **Very** **low** | Low | Moderate | High |  |  | No included studies |
| **Values** | Important uncertainty or variability | Possibly important uncertainty or variability | **Probably no important uncertainty or variability** | No important uncertainty or variability |  |  |  |
| **Balance of effects** | Favors the comparison | Probably favors the comparison | Does not favor either the intervention or the comparison | **Probably favors the intervention** | Favors the intervention | Varies | Don't know |
| **Acceptability** | No | Probably no | **Probably** **yes** | Yes |  | Varies | Don't know |
| **Feasibility** | No | Probably no | **Probably** **yes** | Yes |  | Varies | Don't know |

# Type of recommendation

| Strong recommendation against the intervention | Conditional recommendation against the intervention | Conditional recommendation for either the intervention or the comparison | **Conditional recommendation for the intervention** | Strong recommendation for the intervention |
| --- | --- | --- | --- | --- |
| ○ | ○ | ○ | • | ○ |

# Conclusions

| Recommendation |
| --- |
| The panel suggests that appendectomy rather than nonoperative treatment be used for acute appendicitis during pregnancy (*conditional recommendation, very low certainty of evidence*). |
|  |

| Justification |
| --- |
|  |

| Subgroup considerations |
| --- |
| Trimester considerations – greater safety concerns depending on trimester?  1^st^ trimester – preg loss? Baseline rate of miscarriage ~25%. Teratogenic effects of anesthesia? No great data.  3^rd^ trimester – inc risk of preterm delivery and uterine injury? Particularly risk on entry.  Variability and need for steroids / monitoring  Complicated vs uncomplicated appendicitis |

| Implementation considerations |
| --- |
| Neuroaxial anesthesia rather than general?  Considerations re need for intraoperative monitoring |

| Monitoring and evaluation |
| --- |
|  |

| Research priorities |
| --- |
| CODA trial – can non op mgmt. work in the pregnant pop? They are also at greater risk for more severe disease.  complicated vs uncomplicated appendicitis  Breakdown demographics by trimester  Ideally RCTs, at least prospective studies |

# References Summary
