# Supplementary material for: SAGES guidelines for the use of laparoscopy during pregnancy
Source: Surg Endosc. 2024 May 3;38(6):2947–63. doi: 10.1007/s00464-024-10810-1 (PMC11133165; doi:10.1007/s00464-024-10810-1)
Supplement: Supplementary file 6 — Supplementary file6 (ZIP 91 kb) [file 464_2024_10810_MOESM6_ESM.zip › 464_2024_10810_MOESM6_ESM/Appendix F KQ2 Evidence to decision table.docx]

| Key Question 2 | |
| --- | --- |
| **Should Laparoscopic appendectomy vs. open appendectomy be used for appendicitis during pregnancy (any trimester)?** | |
| **Population:** | appendicitis during pregnancy (any trimester) |
| **Intervention:** | KQ2 Laparoscopic appendectomy |
| **Comparison:** | open appendectomy |
| **Main outcomes:** | C-Section; Delivery; Neonatal death; NICU; Preg loss - all; Preg loss - <20; Preg loss - >20; Preterm; Readmit; Sepsis; |
| **Setting:** |  |
| **Perspective:** |  |
| **Background:** |  |
| **Conflict of interests:** |  |

# Assessment

| Problem Is the problem a priority? | | |
| --- | --- | --- |
| Judgement | Research evidence | Additional considerations |
| ○ No ○ Probably no ○ Probably yes **•** **Yes** ○ Varies ○ Don't know |  |  |
| Desirable Effects How substantial are the desirable anticipated effects? | | |
| Judgement | Research evidence | Additional considerations |
| ○ Trivial • **Small** ○ Moderate ○ Large ○ Varies ○ Don't know | \| **Outcomes** \| **№ of participants (studies) Follow-up** \| **Certainty of the evidence (GRADE)** \| **Relative effect (95% CI)** \| **Anticipated absolute effects^*^ (95% CI)** \| \| \| --- \| --- \| --- \| --- \| --- \| --- \| \| **Risk with open appendectomy** \| **Risk difference with KQ2 Laparoscopic appendectomy** \| \| Delivery \| 52 (2 observational studies) \| ⨁◯◯◯ Very low^a^ \| **OR 0.94** (0.03 to 26.65) \| Study population \| \| \| 34 per 1,000 \| **2 fewer per 1,000** (33 fewer to 453 more) \| \| Preterm \| 5983 (21 observational studies) \| ⨁◯◯◯ Very low^b^ \| **OR 0.86** (0.55 to 1.35) \| Study population \| \| \| 90 per 1,000 \| **12 fewer per 1,000** (38 fewer to 28 more) \| \| Readmit \| 1094 (3 observational studies) \| ⨁◯◯◯ Very low^c^ \| **OR 0.79** (0.41 to 1.51) \| Study population \| \| \| 39 per 1,000 \| **8 fewer per 1,000** (23 fewer to 19 more) \| \| Sepsis \| 2341 (2 observational studies) \| ⨁◯◯◯ Very low^b^ \| **OR 0.58** (0.20 to 1.69) \| Study population \| \| \| 8 per 1,000 \| **3 fewer per 1,000** (6 fewer to 5 more) \|  1. Included studies with an unclear risk of bias on the Newcastle-Ottawa scale due to potential biases in the selection of patients and comparability of groups. 2. Included studies with a high risk of bias on the Newcastle-Ottawa scale due to comparability of the groups. 3. Included studies with an unclear risk of bias on the Newcastle-Ottawa scale due to potential biases in the comparability of groups. | small 100% |
| Undesirable Effects How substantial are the undesirable anticipated effects? | | |
| Judgement | Research evidence | Additional considerations |
| ○ Large ○ Moderate ○ Small • **Trivial** ○ Varies ○ Don't know | \| **Outcomes** \| **№ of participants (studies) Follow-up** \| **Certainty of the evidence (GRADE)** \| **Relative effect (95% CI)** \| **Anticipated absolute effects^*^ (95% CI)** \| \| \| --- \| --- \| --- \| --- \| --- \| --- \| \| **Risk with open appendectomy** \| **Risk difference with KQ2 Laparoscopic appendectomy** \| \| C-Section \| 2266 (11 observational studies) \| ⨁◯◯◯ Very low^a^ \| **OR 1.10** (0.91 to 1.33) \| Study population \| \| \| 385 per 1,000 \| **23 more per 1,000** (22 fewer to 69 more) \| \| NICU \| 31 (1 observational study) \| ⨁◯◯◯ Very low^b^ \| **OR 2.31** (0.09 to 61.41) \| Study population \| \| \| 0 per 1,000 \| **0 fewer per 1,000** (0 fewer to 0 fewer) \| \| Preg loss - all \| 6188 (27 observational studies) \| ⨁◯◯◯ Very low^a^ \| **OR 1.93** (1.39 to 2.70) \| Study population \| \| \| 31 per 1,000 \| **27 more per 1,000** (11 more to 48 more) \| \| Preg loss - <20 \| 525 (11 observational studies) \| ⨁◯◯◯ Very low^a^ \| **OR 3.20** (0.91 to 11.22) \| Study population \| \| \| 7 per 1,000 \| **16 more per 1,000** (1 fewer to 69 more) \| \| Preg loss - >20 \| 429 (8 observational studies) \| ⨁◯◯◯ Very low^a^ \| **OR 1.47** (0.15 to 14.52) \| Study population \| \| \| 4 per 1,000 \| **2 more per 1,000** (4 fewer to 57 more) \|  1. Included studies with a high risk of bias on the Newcastle-Ottawa scale due to comparability of the groups. 2. Included studies with an unclear risk of bias on the Newcastle-Ottawa scale due to potential biases in the selection of patients. | trivial 100% |
| Certainty of evidence What is the overall certainty of the evidence of effects? | | |
| Judgement | Research evidence | Additional considerations |
| • **Very low** ○ Low ○ Moderate ○ High ○ No included studies |  |  |
| Values Is there important uncertainty about or variability in how much people value the main outcomes? | | |
| Judgement | Research evidence | Additional considerations |
| ○ Important uncertainty or variability ○ Possibly important uncertainty or variability **• Probably no important uncertainty or variability** ○ No important uncertainty or variability |  | Probably no important uncertainty or variability 100% |
| Balance of effects Does the balance between desirable and undesirable effects favor the intervention or the comparison? | | |
| Judgement | Research evidence | Additional considerations |
| ○ Favors the comparison ○ Probably favors the comparison • **Does not favor either the intervention or the comparison** ○ Probably favors the intervention ○ Favors the intervention ○ Varies ○ Don't know |  | Does not favor either the intervention or the comparison 80%  Probably favors the intervention 20% |
| Acceptability Is the intervention acceptable to key stakeholders? | | |
| Judgement | Research evidence | Additional considerations |
| ○ No ○ Probably no ○ Probably yes • **Yes** ○ Varies ○ Don't know |  | Yes 100% |
| Feasibility Is the intervention feasible to implement? | | |
| Judgement | Research evidence | Additional considerations |
| ○ No ○ Probably no ○ Probably yes • **Yes** ○ Varies ○ Don't know |  | Yes 100% |

# Summary of judgements

|  | **Judgement** | | | | | | |
| --- | --- | --- | --- | --- | --- | --- | --- |
| **Problem** | No | Probably no | Probably yes | **Yes** |  | Varies | Don't know |
| **Desirable Effects** | Trivial | **Small** | Moderate | Large |  | Varies | Don't know |
| **Undesirable Effects** | Large | Moderate | Small | **Trivial** |  | Varies | Don't know |
| **Certainty of evidence** | **Very low** | Low | Moderate | High |  |  | No included studies |
| **Values** | Important uncertainty or variability | Possibly important uncertainty or variability | **Probably no important uncertainty or variability** | No important uncertainty or variability |  |  |  |
| **Balance of effects** | Favors the comparison | Probably favors the comparison | **Does not favor either the intervention or the comparison** | Probably favors the intervention | Favors the intervention | Varies | Don't know |
| **Acceptability** | No | Probably no | Probably yes | **Yes** |  | Varies | Don't know |
| **Feasibility** | No | Probably no | Probably yes | **Yes** |  | Varies | Don't know |

# Type of recommendation

| Strong recommendation against the intervention | Conditional recommendation against the intervention | Conditional recommendation for either the intervention or the comparison | Conditional recommendation for the intervention | Strong recommendation for the intervention |
| --- | --- | --- | --- | --- |
| ○ | ○ | ○ | ○ | ○ |

# Conclusions

| Recommendation |
| --- |
|  |
|  |

| Justification |
| --- |
| In light of a very limited and low quality evidence base, the panel agreed on basing the final recommendation on expert opinion. |

| Subgroup considerations |
| --- |
| By trimester, how complex the appendicitis is/severity of disease, how sick/stable the patient is, prior surgical history With multiple gestations, uterine size may be greater and cause increased difficulty with the laparoscopic approach |

| Implementation considerations |
| --- |
| Decubitus positioning, |

| Monitoring and evaluation |
| --- |
| Conversion rates.  Tracking maternal/fetal outcomes including past delivery |

| Research priorities |
| --- |
| RCT lap vs open appendectomy in third trimester.  Evaluating the utility of intraoperative fetal monitoring by gestational age.  Multi-institutional collaborations or utilization of databases eg NSQIP that have more granular clinical data could be used to evaluate appendicitis in the pregnant population. |
