# Supplementary material for: SAGES guidelines for the use of laparoscopy during pregnancy
Source: Surg Endosc. 2024 May 3;38(6):2947–63. doi: 10.1007/s00464-024-10810-1 (PMC11133165; doi:10.1007/s00464-024-10810-1)
Supplement: Supplementary file 6 — Supplementary file6 (ZIP 91 kb) [file 464_2024_10810_MOESM6_ESM.zip › 464_2024_10810_MOESM6_ESM/Appendix F KQ3 Evidence to decision table.docx]

| Key Question 3 | |
| --- | --- |
| **Should Cholecystectomy vs. Medical Treatment be used for biliary disease in pregnancy?** | |
| **Population:** | biliary disease in pregnancy |
| **Intervention:** | Cholecystectomy |
| **Comparison:** | Medical Treatment |
| **Main outcomes:** | Bile leak; C-Section; C-Section - Cholecystitis only; C-Section - Others; Delivery during admission; IUGR; IUGR - Cholecystitis only; IUGR - Others; Neonatal death; NICU; Pre-eclampsia; Pre-eclampsia - Cholecystitis only; Pre-eclampsia - Others; Preg loss - all; Preg loss - all - Cholecystitis only; Preg loss - all - Others; Preg loss - <20; Preg loss - >20; Preterm; Preterm - Cholecystitis only; Preterm - Others; Readmit; Readmit - Cholecystitis only; Readmit - Others; Sepsis; Sepsis - Cholecystitis only; Sepsis - Others; |
| **Setting:** |  |
| **Perspective:** |  |
| **Background:** |  |
| **Conflict of interests:** |  |

# Assessment

| Problem Is the problem a priority? | | |
| --- | --- | --- |
| Judgement | Research evidence | Additional considerations |
| ○ No ○ Probably no ○ Probably yes • **Yes** ○ Varies ○ Don't know |  |  |
| Desirable Effects How substantial are the desirable anticipated effects? | | |
| Judgement | Research evidence | Additional considerations |
| ○ Trivial ○ Small ○ Moderate ○ Large ○ Varies ○ Don't know | \| **Outcomes** \| **№ of participants (studies) Follow-up** \| **Certainty of the evidence (GRADE)** \| **Relative effect (95% CI)** \| **Anticipated absolute effects^*^ (95% CI)** \| \| \| --- \| --- \| --- \| --- \| --- \| --- \| \| **Risk with Medical Treatment** \| **Risk difference with Cholecystectomy** \| \| C-Section \| 31616 (9 observational studies) \| ⨁◯◯◯ Very low^a,b^ \| **OR 0.87** (0.36 to 2.10) \| Study population \| \| \| 377 per 1,000 \| **32 fewer per 1,000** (198 fewer to 183 more) \| \| C-Section - Cholecystitis only \| 6390 (1 observational study) \| ⨁⨁◯◯ Low \| **OR 0.19** (0.16 to 0.23) \| Study population \| \| \| 249 per 1,000 \| **190 fewer per 1,000** (199 fewer to 178 fewer) \| \| Delivery during admission \| 180 (3 observational studies) \| ⨁◯◯◯ Very low^a,b^ \| **OR 0.60** (0.22 to 1.67) \| Study population \| \| \| 226 per 1,000 \| **77 fewer per 1,000** (165 fewer to 102 more) \| \| IUGR - Cholecystitis only \| 6390 (1 observational study) \| ⨁⨁◯◯ Low \| **OR 0.21** (0.12 to 0.37) \| Study population \| \| \| 26 per 1,000 \| **21 fewer per 1,000** (23 fewer to 16 fewer) \| \| Neonatal death \| 227 (3 observational studies) \| ⨁◯◯◯ Very low^a,b^ \| **OR 0.94** (0.04 to 20.73) \| Study population \| \| \| 14 per 1,000 \| **1 fewer per 1,000** (14 fewer to 216 more) \| \| NICU \| 120 (2 observational studies) \| ⨁◯◯◯ Very low^a,b^ \| **OR 0.20** (0.02 to 1.74) \| Study population \| \| \| 182 per 1,000 \| **139 fewer per 1,000** (177 fewer to 97 more) \| \| Pre-eclampsia - Cholecystitis only \| 6390 (1 observational study) \| ⨁⨁◯◯ Low \| **OR 0.56** (0.48 to 0.66) \| Study population \| \| \| 153 per 1,000 \| **61 fewer per 1,000** (73 fewer to 46 fewer) \| \| Pregnancy loss - all \| 6756 (7 observational studies) \| ⨁◯◯◯ Very low^a,b^ \| **OR 0.70** (0.39 to 1.25) \| Study population \| \| \| 9 per 1,000 \| **3 fewer per 1,000** (6 fewer to 2 more) \| \| Preg loss - all - Cholecystitis only \| 6390 (1 observational study) \| ⨁◯◯◯ Very low^b^ \| **OR 0.61** (0.33 to 1.13) \| Study population \| \| \| 9 per 1,000 \| **4 fewer per 1,000** (6 fewer to 1 more) \| \| Preterm - Cholecystitis only \| 6390 (1 observational study) \| ⨁⨁◯◯ Low \| **OR 0.35** (0.27 to 0.44) \| Study population \| \| \| 101 per 1,000 \| **63 fewer per 1,000** (71 fewer to 54 fewer) \| \| Readmit \| 31446 (7 observational studies) \| ⨁◯◯◯ Very low^a^ \| **OR 0.39** (0.15 to 0.98) \| Study population \| \| \| 70 per 1,000 \| **42 fewer per 1,000** (59 fewer to 1 fewer) \| \| Readmit - Cholecystitis only \| 6390 (1 observational study) \| ⨁⨁◯◯ Low \| **OR 0.52** (0.45 to 0.61) \| Study population \| \| \| 187 per 1,000 \| **80 fewer per 1,000** (93 fewer to 64 fewer) \|  1. Some of the included studies which contributed significantly to the overall effect size were deemed to be at a high risk of bias on the Newcastle-Ottawa scale due to comparability. 2. There was a wide range of effects that crosses several clinically relevant thresholds. 3. There was serious inconsistency between some of the included studies, with non-overlapping confidence intervals. | Overall 80% moderate, 20% small  Cholecystitis only 80% large, 20% moderate  Preterm birth, c-section noted to be especially important in this vote. |
| Undesirable Effects How substantial are the undesirable anticipated effects? | | |
| Judgement | Research evidence | Additional considerations |
| ○ Large ○ Moderate ○ Small ○ Trivial ○ Varies ○ Don't know | \| **Outcomes** \| **№ of participants (studies) Follow-up** \| **Certainty of the evidence (GRADE)** \| **Relative effect (95% CI)** \| **Anticipated absolute effects^*^ (95% CI)** \| \| \| --- \| --- \| --- \| --- \| --- \| --- \| \| **Risk with Medical Treatment** \| **Risk difference with Cholecystectomy** \| \| Bile leak \| 23301 (6 observational studies) \| ⨁◯◯◯ Very low^a,b^ \| **OR 1.06** (0.17 to 6.53) \| Study population \| \| \| 13 per 1,000 \| **1 more per 1,000** (11 fewer to 66 more) \| \| IUGR \| 6587 (4 observational studies) \| ⨁◯◯◯ Very low^a,b,c^ \| **OR 1.28** (0.12 to 13.29) \| Study population \| \| \| 26 per 1,000 \| **7 more per 1,000** (23 fewer to 239 more) \| \| Pre-eclampsia \| 29447 (4 observational studies) \| ⨁◯◯◯ Very low^a,c^ \| **OR 1.94** (0.47 to 8.04) \| Study population \| \| \| 30 per 1,000 \| **26 more per 1,000** (16 fewer to 168 more) \| \| Pregnancy loss - <20 \| 340 (4 observational studies) \| ⨁◯◯◯ Very low^a,b^ \| **OR 2.30** (0.33 to 16.18) \| Study population \| \| \| 9 per 1,000 \| **11 more per 1,000** (6 fewer to 118 more) \| \| Pregnancy loss - >20 \| 287 (4 observational studies) \| ⨁◯◯◯ Very low^a,b^ \| **OR 3.87** (0.39 to 38.66) \| Study population \| \| \| 5 per 1,000 \| **15 more per 1,000** (3 fewer to 167 more) \| \| Preterm \| 39108 (10 observational studies) \| ⨁◯◯◯ Very low^a,b,c^ \| **OR 1.77** (0.73 to 4.30) \| Study population \| \| \| 89 per 1,000 \| **58 more per 1,000** (22 fewer to 207 more) \| \| Sepsis \| 7677 (3 observational studies) \| ⨁◯◯◯ Very low^a,b^ \| **OR 1.66** (1.11 to 2.47) \| Study population \| \| \| 18 per 1,000 \| **11 more per 1,000** (2 more to 25 more) \| \| Sepsis - Cholecystitis only \| 6390 (1 observational study) \| ⨁⨁◯◯ Low \| **OR 1.83** (1.32 to 2.55) \| Study population \| \| \| 17 per 1,000 \| **14 more per 1,000** (5 more to 26 more) \|  1. Some of the included studies which contributed significantly to the overall effect size were deemed to be at a high risk of bias on the Newcastle-Ottawa scale due to comparability. 2. There was a wide range of effects that crosses several clinically relevant thresholds. 3. There was serious inconsistency between some of the included studies, with non-overlapping confidence intervals. | Overall 100% small  Cholecystitis only 100% small |
| Certainty of evidence What is the overall certainty of the evidence of effects? | | |
| Judgement | Research evidence | Additional considerations |
| ○ Very low ○ Low ○ Moderate ○ High ○ No included studies |  | Overall 100% very low  Cholecystitis only 100% low |
| Values Is there important uncertainty about or variability in how much people value the main outcomes? | | |
| Judgement | Research evidence | Additional considerations |
| ○ Important uncertainty or variability ○ Possibly important uncertainty or variability ○ Probably no important uncertainty or variability ○ No important uncertainty or variability |  | Overall 100% Probably no important uncertainty or variability  Cholecystitis only 100% Probably no important uncertainty or variability |
| Balance of effects Does the balance between desirable and undesirable effects favor the intervention or the comparison? | | |
| Judgement | Research evidence | Additional considerations |
| ○ Favors the comparison ○ Probably favors the comparison ○ Does not favor either the intervention or the comparison ○ Probably favors the intervention ○ Favors the intervention ○ Varies ○ Don't know |  | Overall 100% Probably favors the intervention  Cholecystitis only 80% Favors the intervention, 20% probably favors the intervention |
| Acceptability Is the intervention acceptable to key stakeholders? | | |
| Judgement | Research evidence | Additional considerations |
| ○ No ○ Probably no ○ Probably yes ○ Yes ○ Varies ○ Don't know |  | Overall 100% probably yes  Cholecystitis only 100% probably yes |
| Feasibility Is the intervention feasible to implement? | | |
| Judgement | Research evidence | Additional considerations |
| ○ No ○ Probably no ○ Probably yes ○ Yes ○ Varies ○ Don't know |  | Overall 100% Yes  Cholecystitis only 100% yes |

# Summary of judgements

|  | **Judgement** | | | | | | |
| --- | --- | --- | --- | --- | --- | --- | --- |
| **Problem** | No | Probably no | Probably yes | Yes |  | Varies | Don't know |
| **Desirable Effects** | Trivial | Small | Moderate | Large |  | Varies | Don't know |
| **Undesirable Effects** | Large | Moderate | Small | Trivial |  | Varies | Don't know |
| **Certainty of evidence** | Very low | Low | Moderate | High |  |  | No included studies |
| **Values** | Important uncertainty or variability | Possibly important uncertainty or variability | Probably no important uncertainty or variability | No important uncertainty or variability |  |  |  |
| **Balance of effects** | Favors the comparison | Probably favors the comparison | Does not favor either the intervention or the comparison | Probably favors the intervention | Favors the intervention | Varies | Don't know |
| **Acceptability** | No | Probably no | Probably yes | Yes |  | Varies | Don't know |
| **Feasibility** | No | Probably no | Probably yes | Yes |  | Varies | Don't know |

# Type of recommendation

| Strong recommendation against the intervention | Conditional recommendation against the intervention | Conditional recommendation for either the intervention or the comparison | **Conditional recommendation for the intervention** | Strong recommendation for the intervention |
| --- | --- | --- | --- | --- |
| ○ | ○ | ○ | • | ○ |

# Conclusions

| Recommendation |
| --- |
| Overall and cholecystitis only – conditional recommendation for the intervention. |
|  |

| Justification |
| --- |
|  |

| Subgroup considerations |
| --- |
| 3^rd^ trimester medical treatment may have a role in patients with biliary colic. |

| Implementation considerations |
| --- |
|  |

| Monitoring and evaluation |
| --- |
|  |

| Research priorities |
| --- |
| Impact of trimester on maternal/fetal outcomes.  Underlying disease severity (sepsis) and how intervention changes outcomes. |

# References Summary
